# Supplementary figures and images for: Constitutively active receptor ADGRA3 signaling induces adipose thermogenesis (part 1 of 2)
Source: eLife. 2024 Dec 24;13:RP100205. doi: 10.7554/eLife.100205 (PMC11668527; doi:10.7554/eLife.100205)

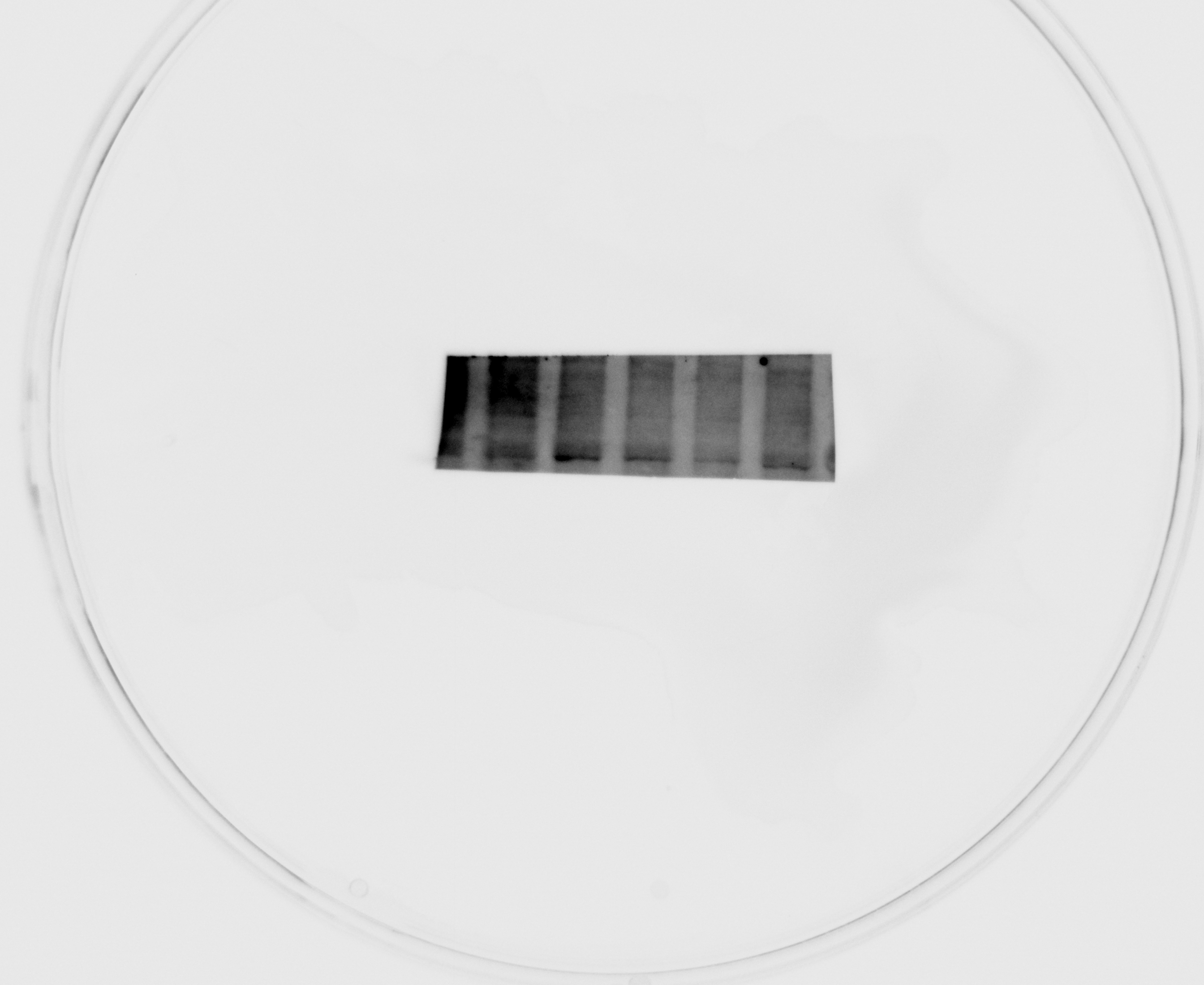

Supplement: Figure 2—source data 1. [file elife-100205-fig2-data1.zip › Figure 2-Source Data 1-Raw uncropped blots/Figure 2B/ADGRA3.tif]

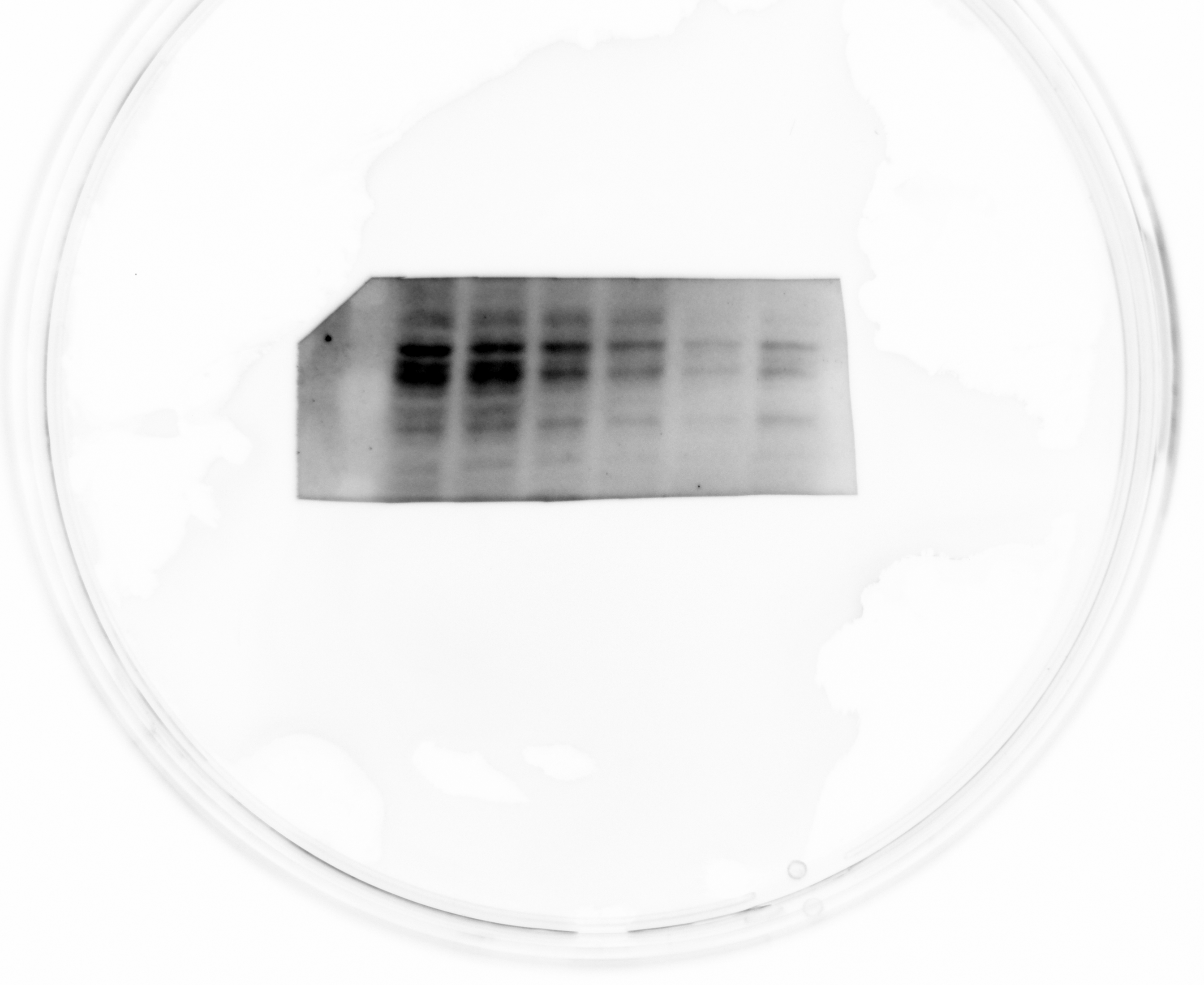

Supplement: Figure 2—source data 1. [file elife-100205-fig2-data1.zip › Figure 2-Source Data 1-Raw uncropped blots/Figure 2B/UCP1.tif]

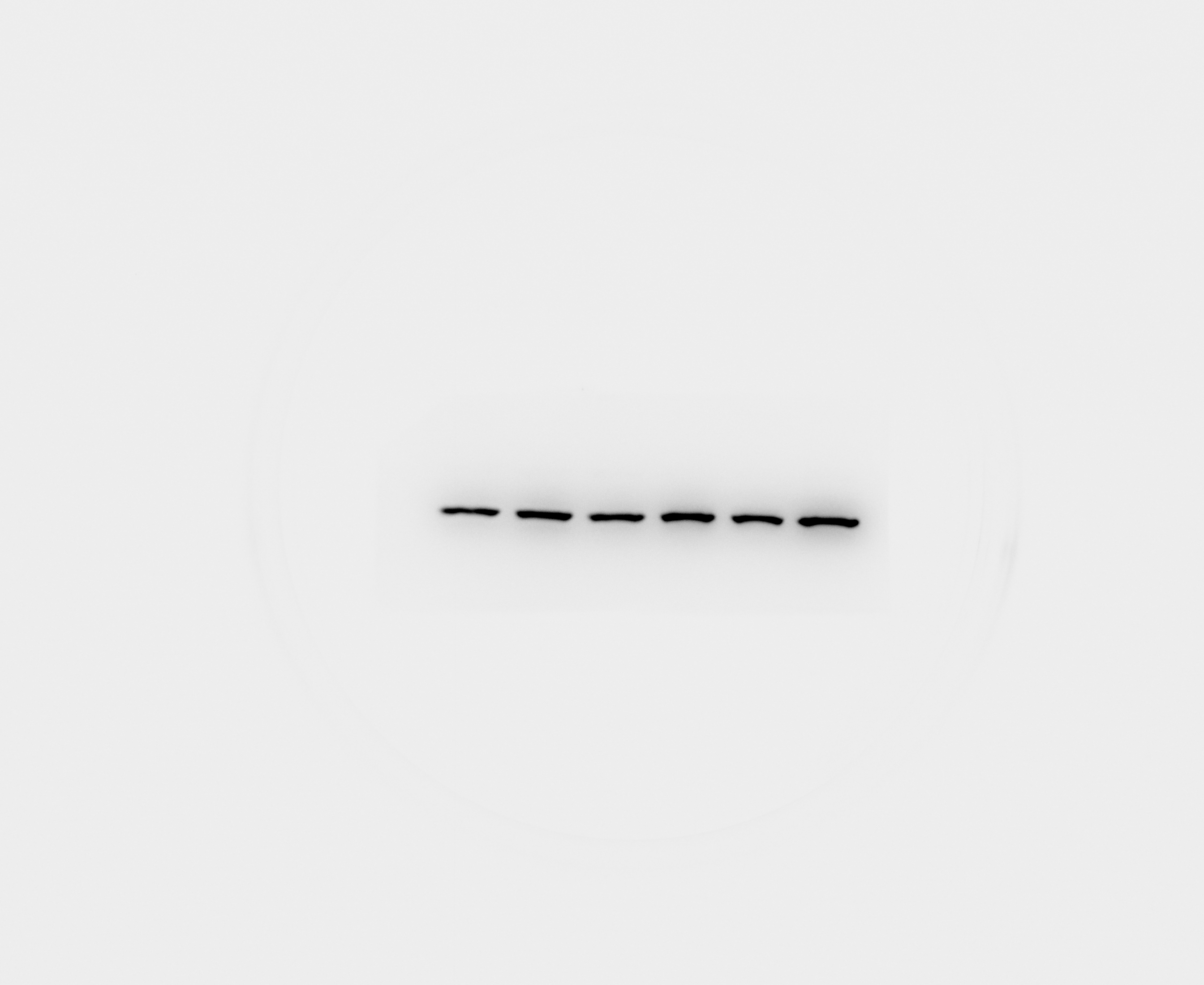

Supplement: Figure 2—source data 1. [file elife-100205-fig2-data1.zip › Figure 2-Source Data 1-Raw uncropped blots/Figure 2B/α-TUBULIN.tif]

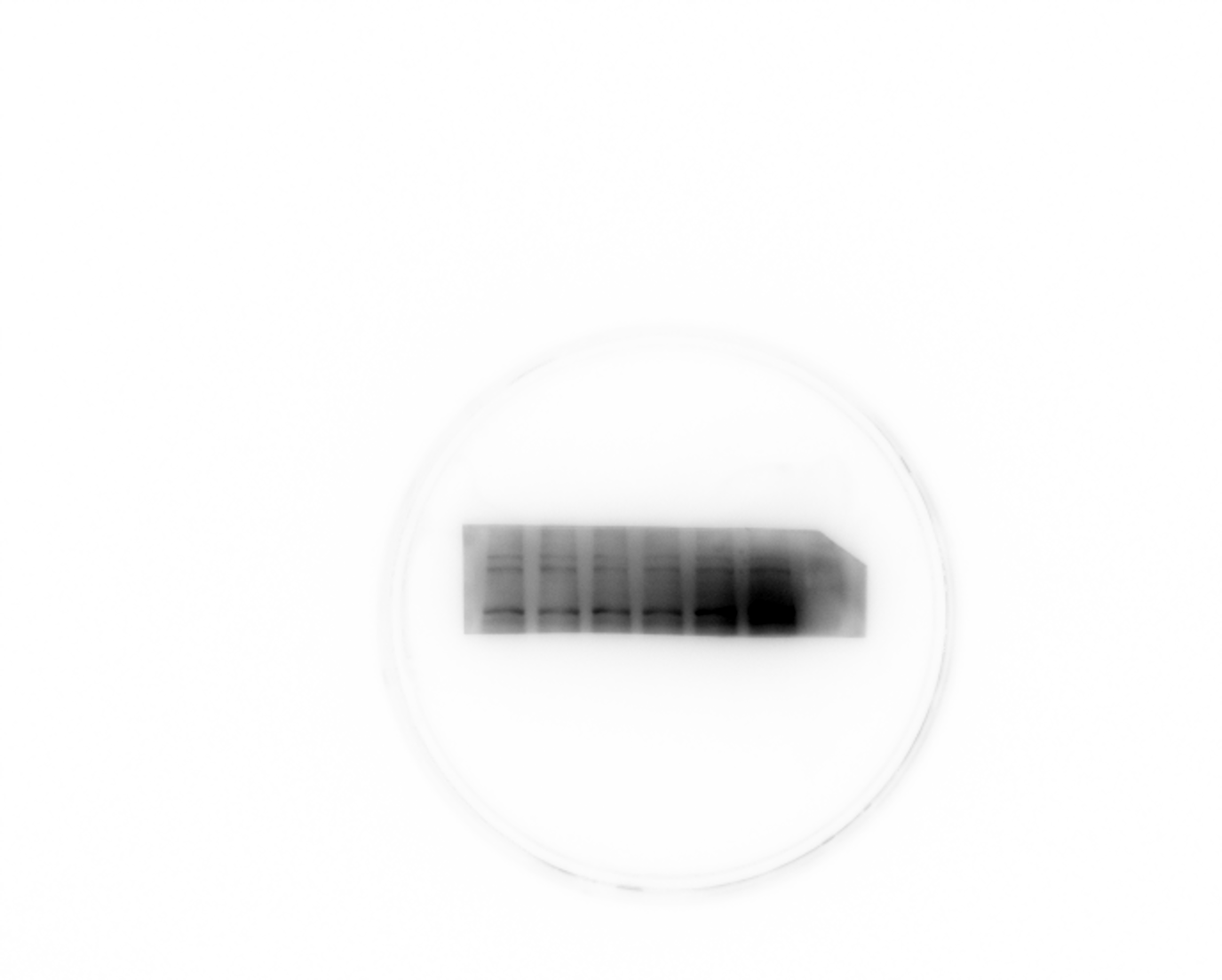

Supplement: Figure 2—source data 1. [file elife-100205-fig2-data1.zip › Figure 2-Source Data 1-Raw uncropped blots/Figure 2I/FLAG.tif]

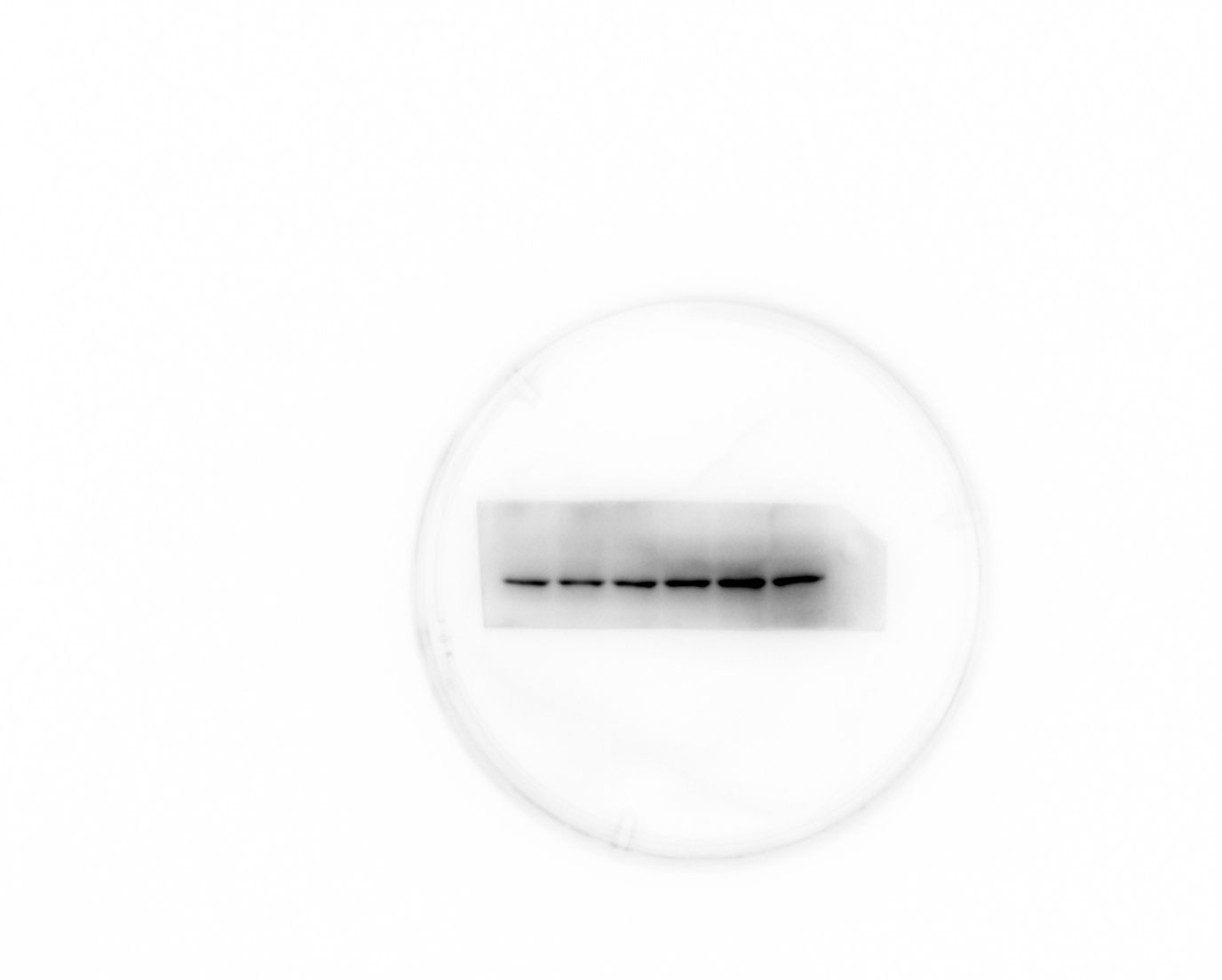

Supplement: Figure 2—source data 1. [file elife-100205-fig2-data1.zip › Figure 2-Source Data 1-Raw uncropped blots/Figure 2I/TUBULIN.tif]

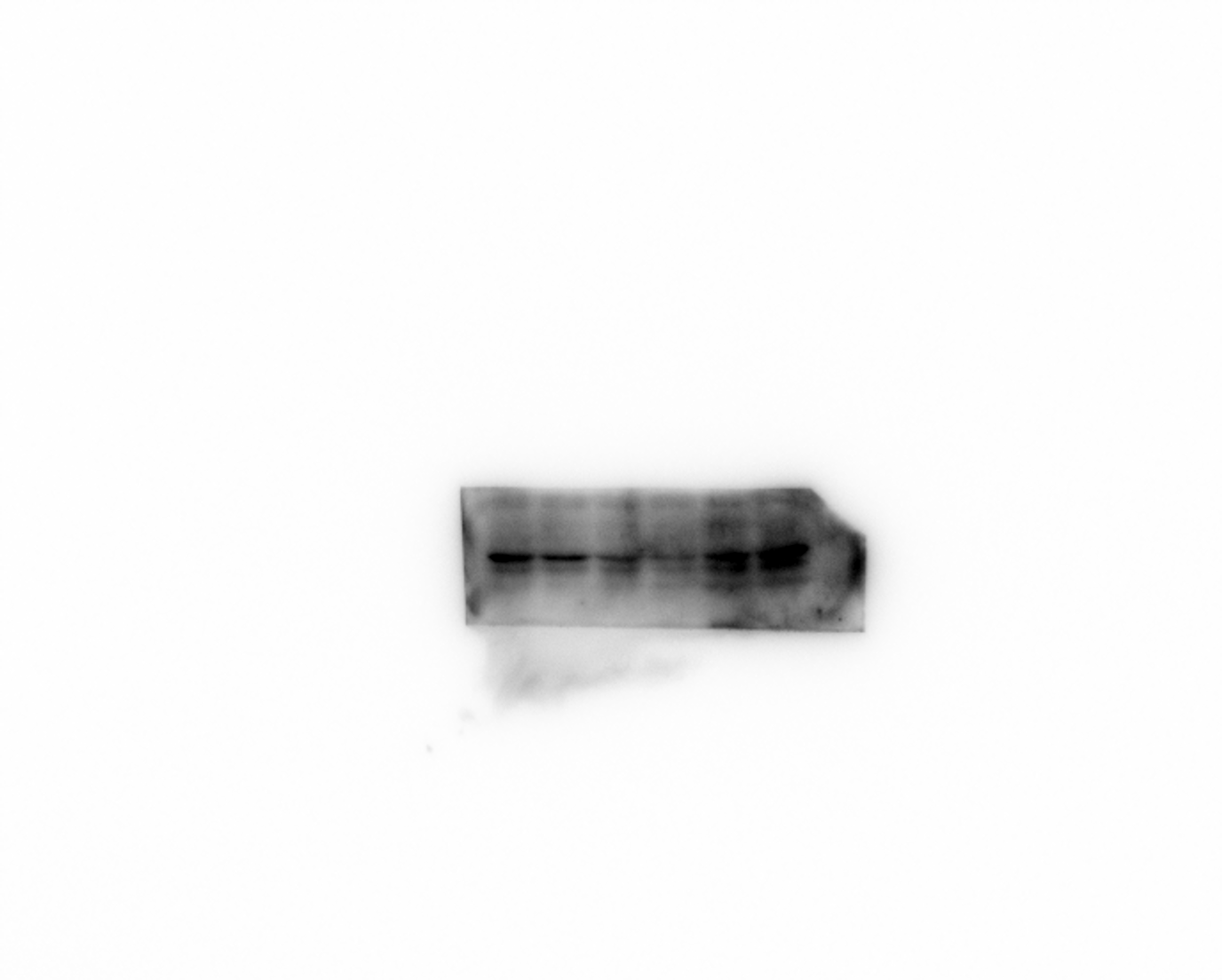

Supplement: Figure 2—source data 1. [file elife-100205-fig2-data1.zip › Figure 2-Source Data 1-Raw uncropped blots/Figure 2I/UCP1.tif]

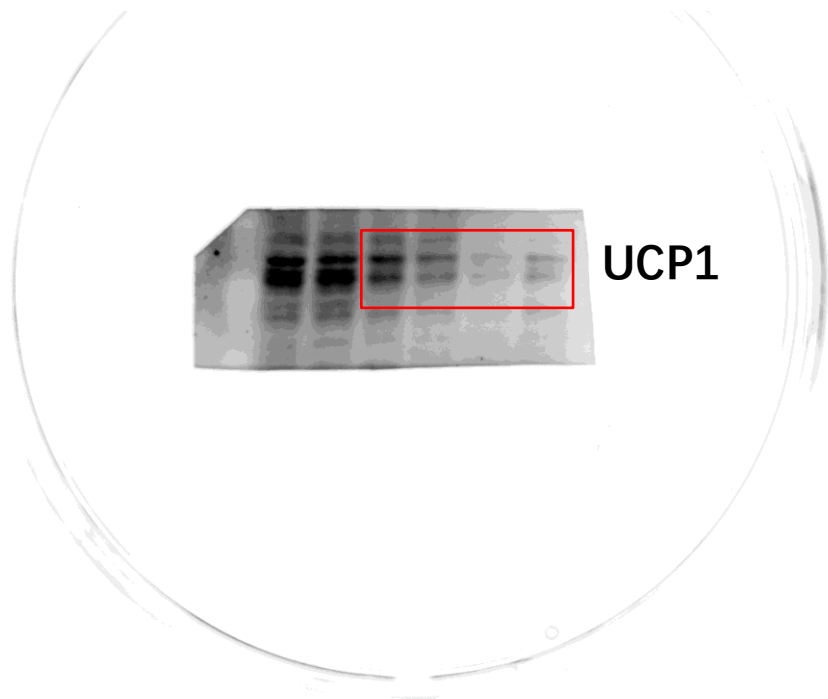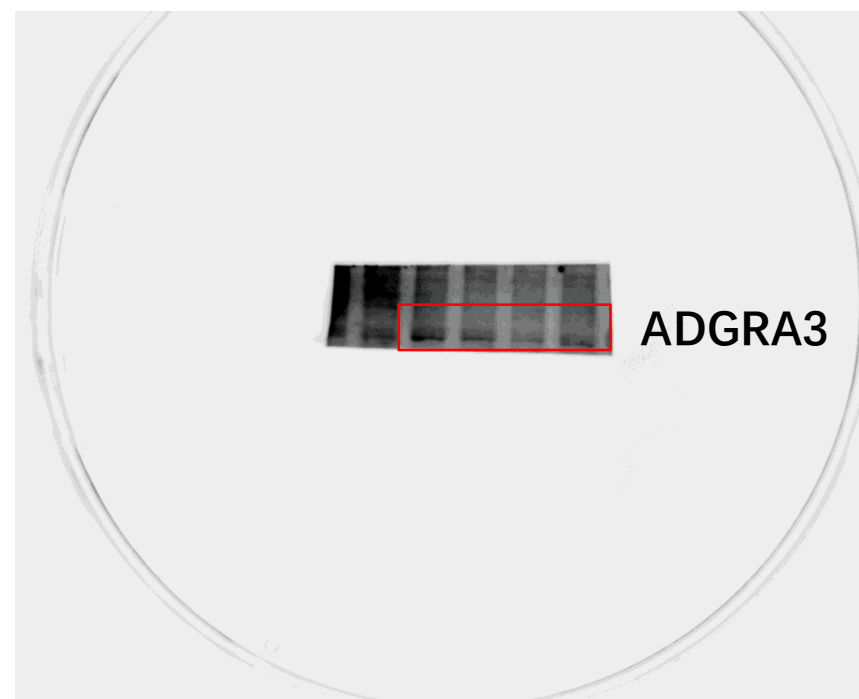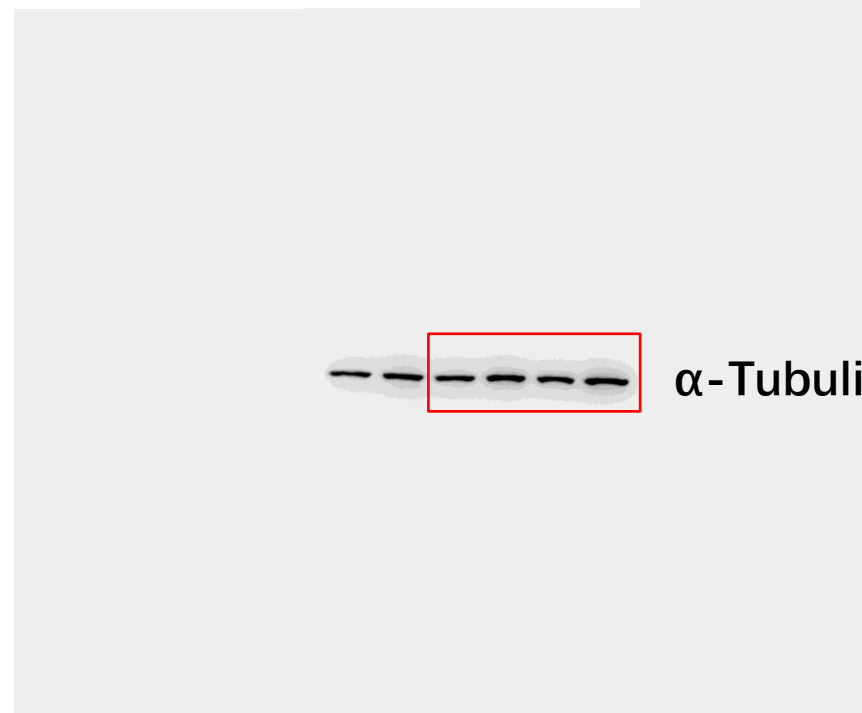

Supplement: Figure 2—source data 2. [file elife-100205-fig2-data2.zip › Figure 2-Source Data 2 -Uncropped and labeled blots/Figure 2B.pdf]

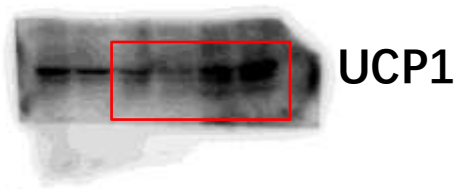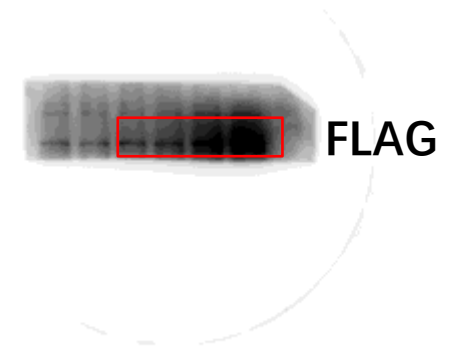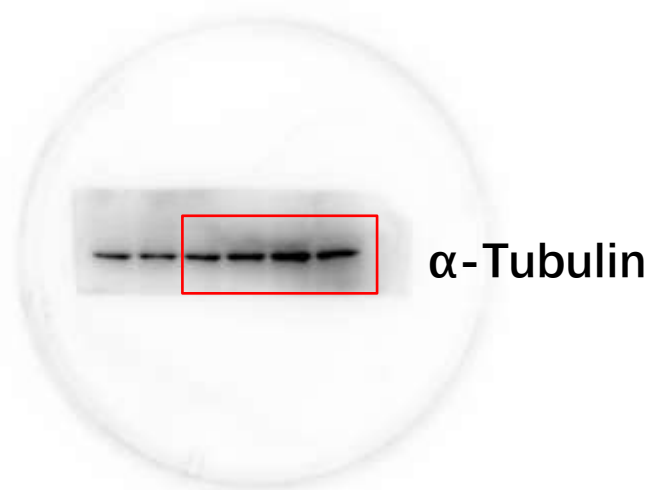

Supplement: Figure 2—source data 2. [file elife-100205-fig2-data2.zip › Figure 2-Source Data 2 -Uncropped and labeled blots/Figure 2I.pdf]

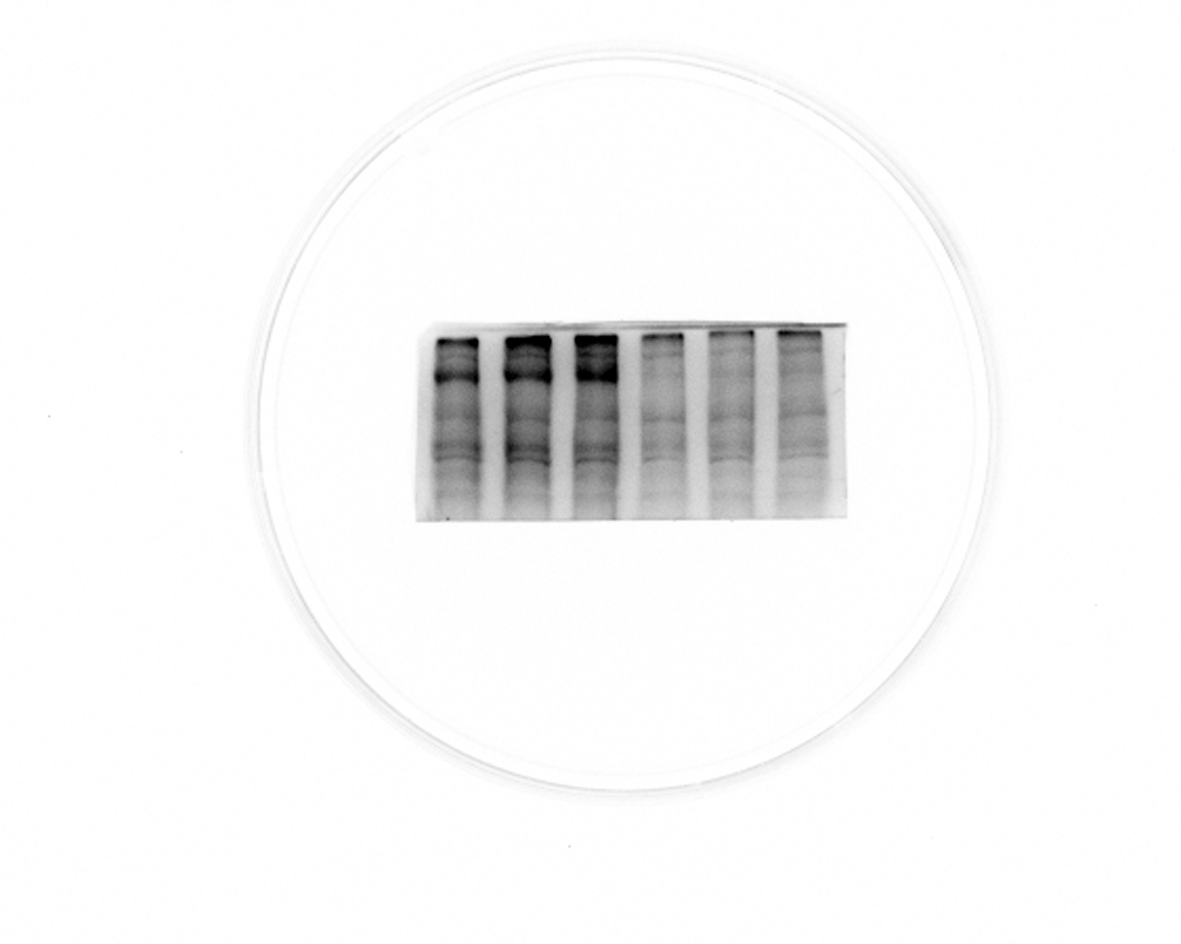

Supplement: Figure 3—source data 1. [file elife-100205-fig3-data1.zip › Figure 3-Source Data 1-Raw uncropped blots/Figure 3H/ADGRA3.tif]

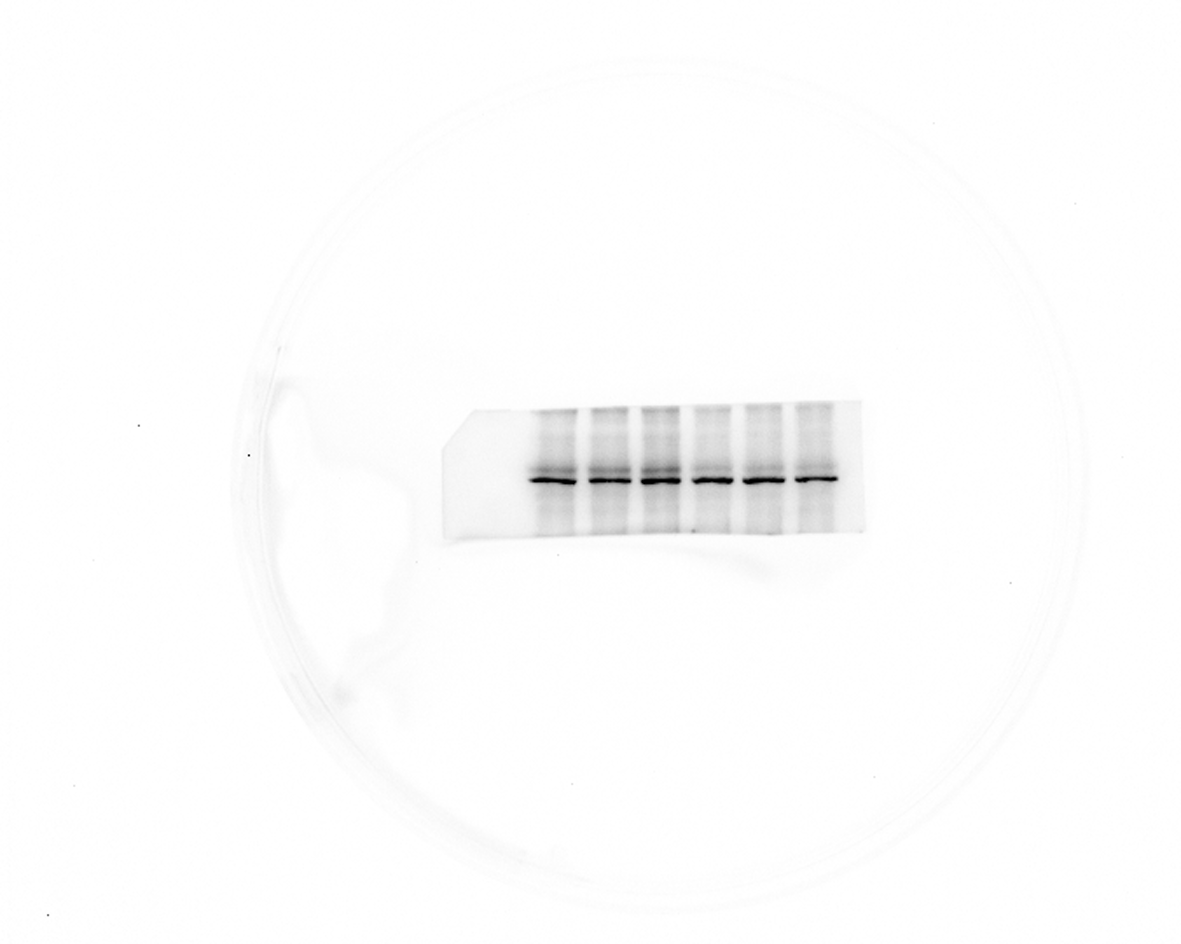

Supplement: Figure 3—source data 1. [file elife-100205-fig3-data1.zip › Figure 3-Source Data 1-Raw uncropped blots/Figure 3H/HSP90.tif]

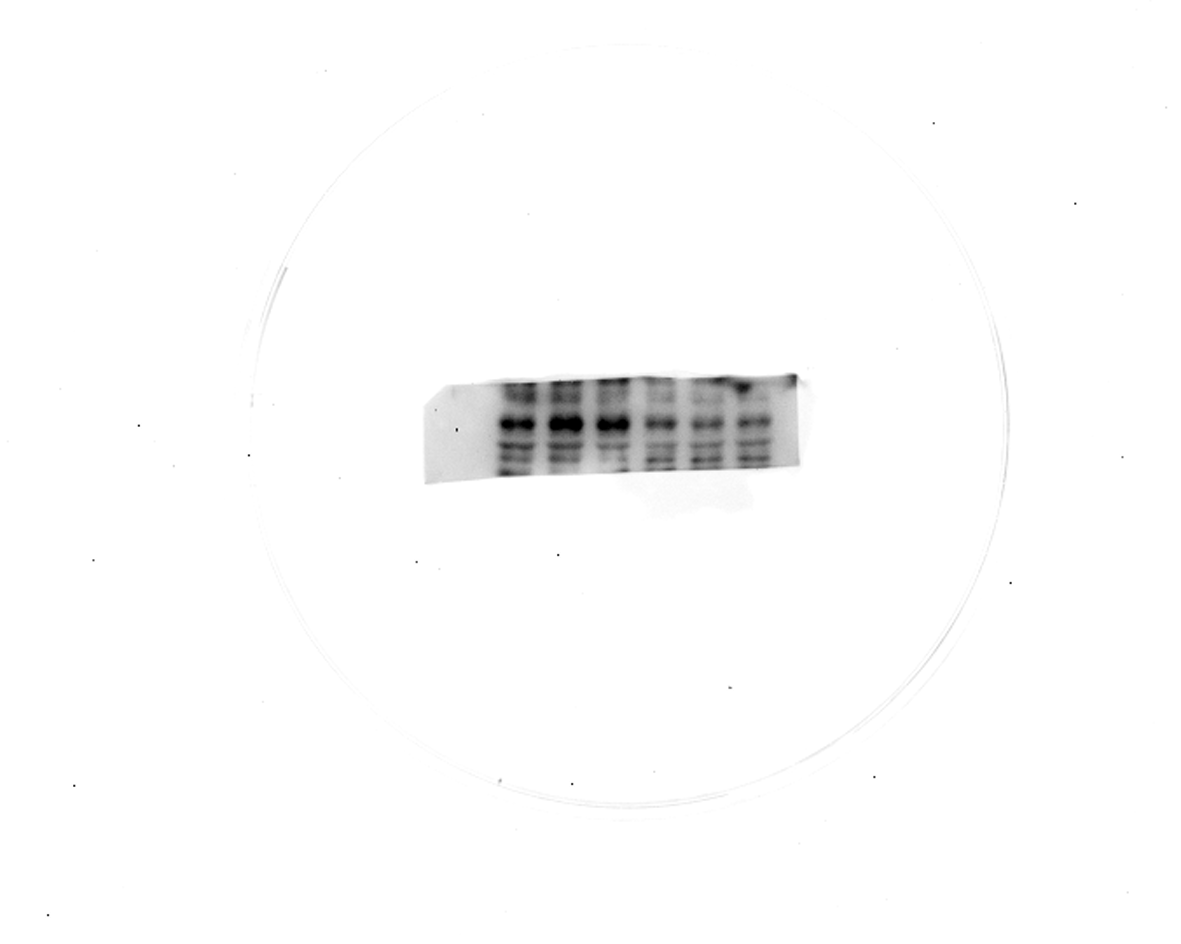

Supplement: Figure 3—source data 1. [file elife-100205-fig3-data1.zip › Figure 3-Source Data 1-Raw uncropped blots/Figure 3H/UCP1.tif]

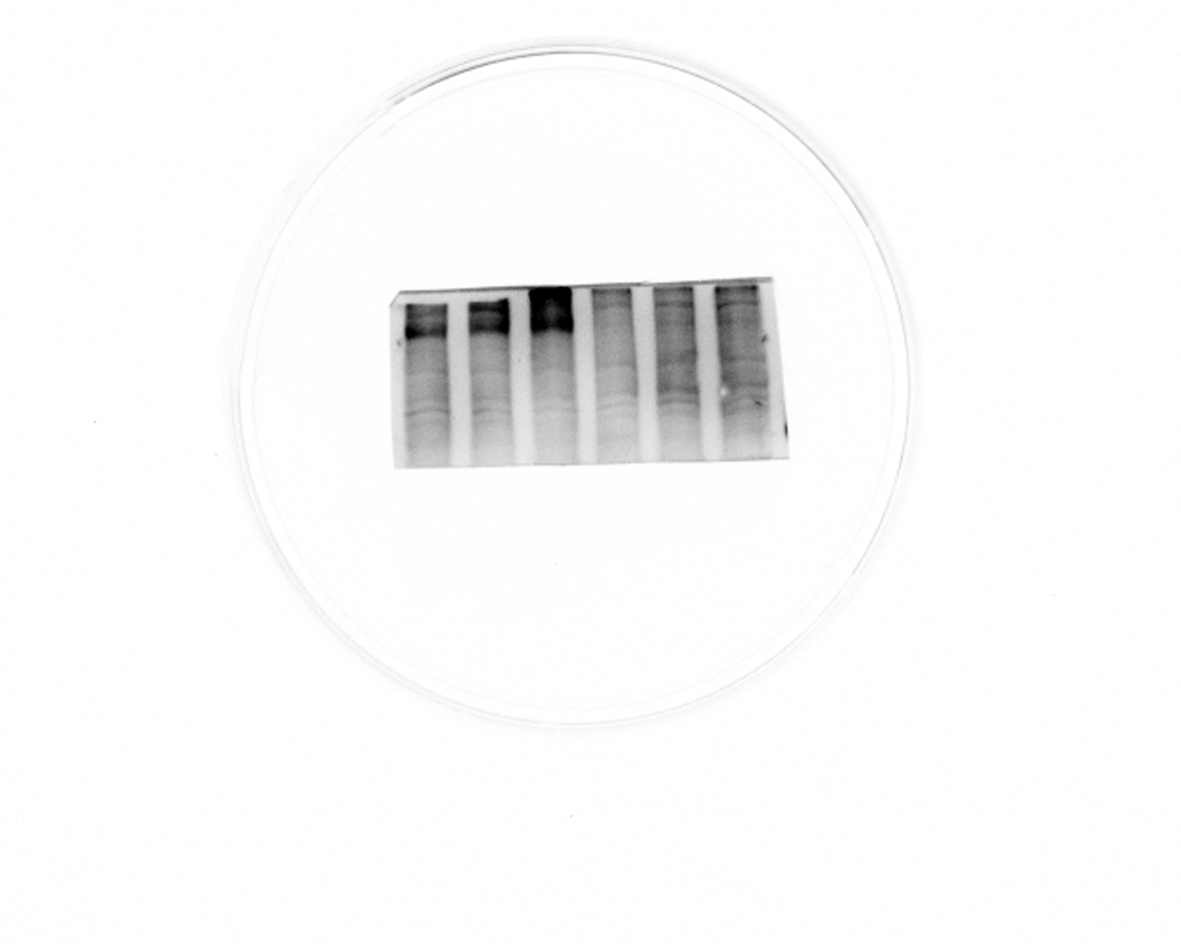

Supplement: Figure 3—source data 1. [file elife-100205-fig3-data1.zip › Figure 3-Source Data 1-Raw uncropped blots/Figure 3I/ADGRA3.tif]

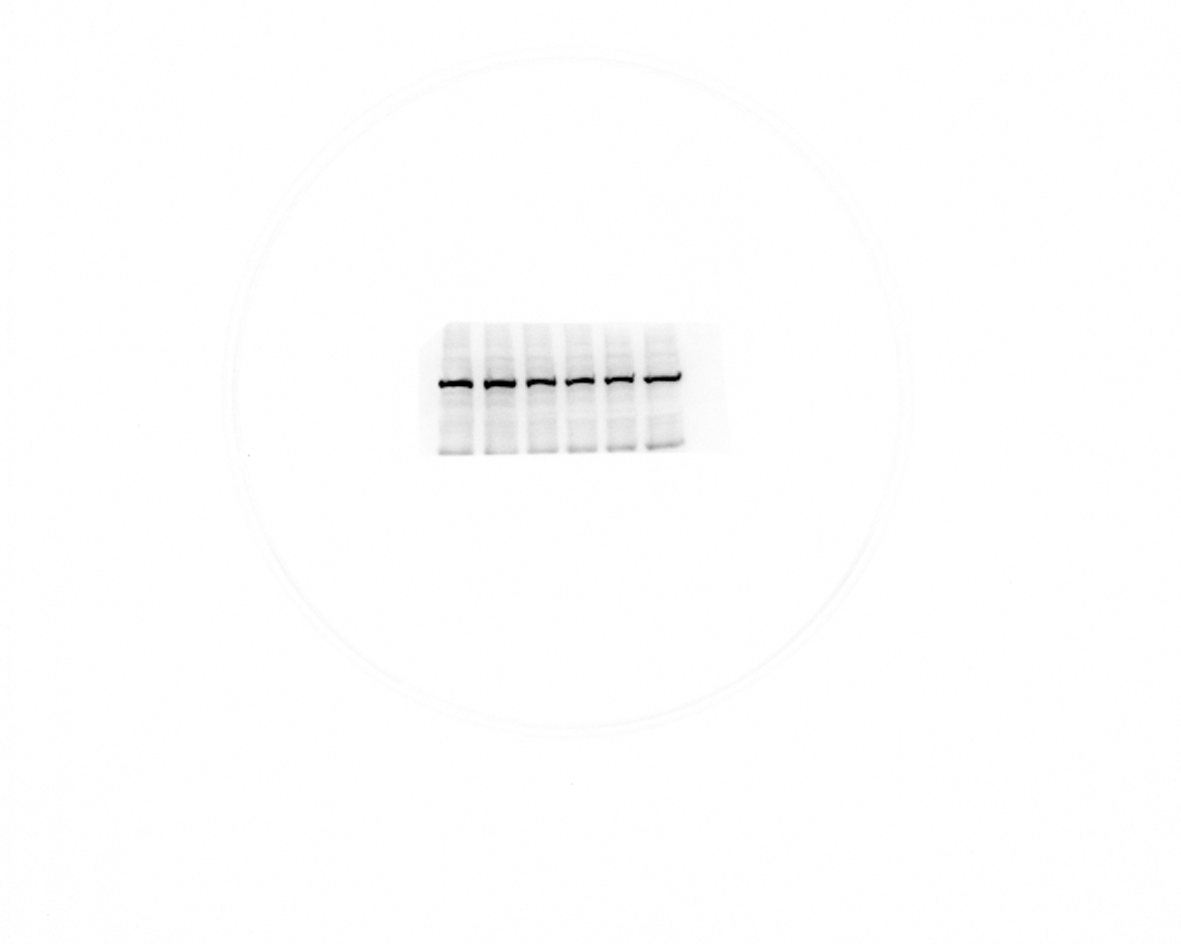

Supplement: Figure 3—source data 1. [file elife-100205-fig3-data1.zip › Figure 3-Source Data 1-Raw uncropped blots/Figure 3I/HSP90.tif]

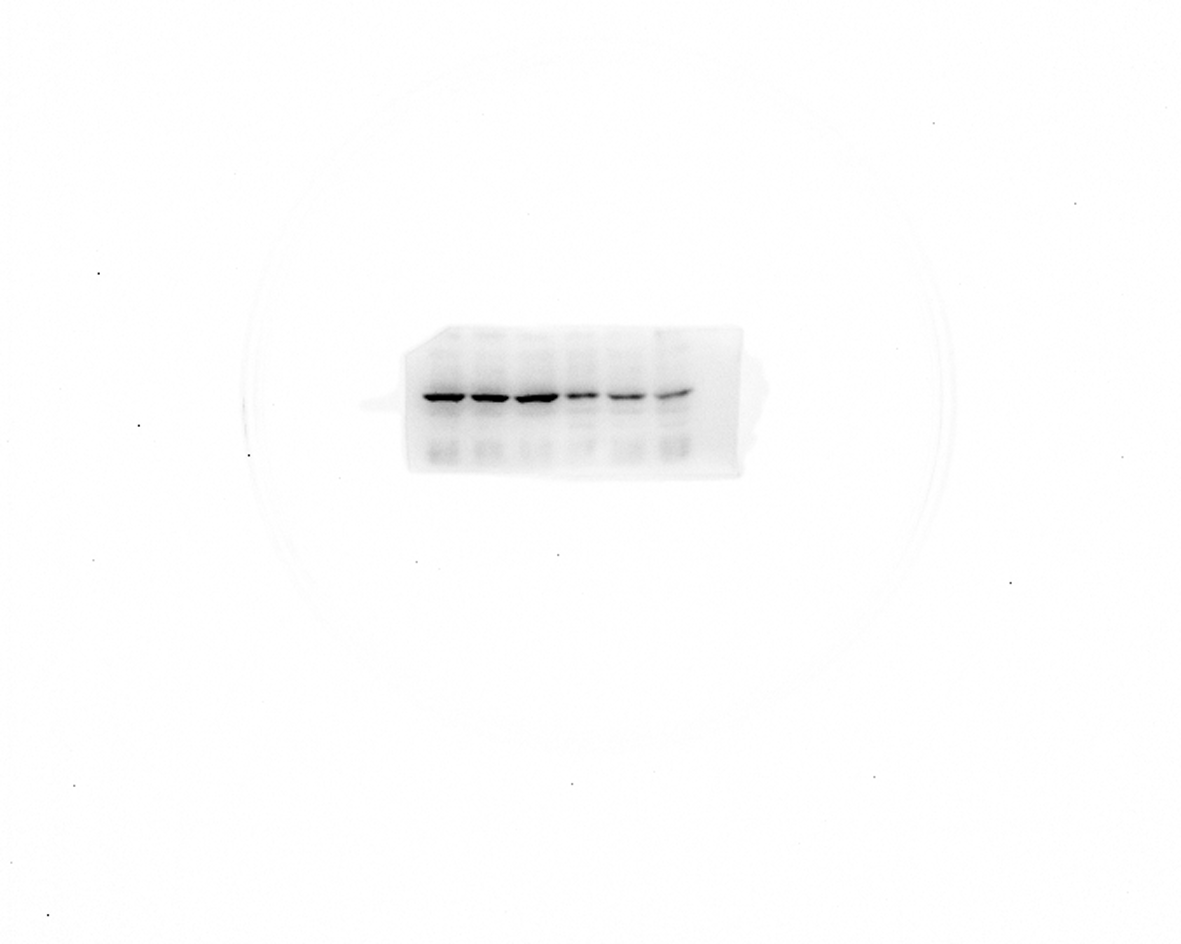

Supplement: Figure 3—source data 1. [file elife-100205-fig3-data1.zip › Figure 3-Source Data 1-Raw uncropped blots/Figure 3I/UCP1.tif]

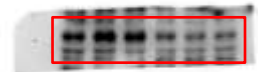

**UCP1**

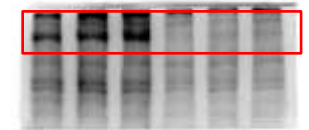

**ADGRA3**

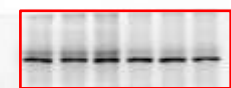

**HSP90**

Supplement: Figure 3—source data 2. [file elife-100205-fig3-data2.zip › Figure 3-Source Data 2 -Uncropped and labeled blots/Figure 3H.pdf]

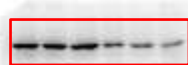

**UCP1**

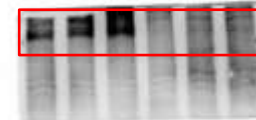

**ADGRA3**

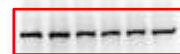

**HSP90**

Supplement: Figure 3—source data 2. [file elife-100205-fig3-data2.zip › Figure 3-Source Data 2 -Uncropped and labeled blots/Figure 3I.pdf]

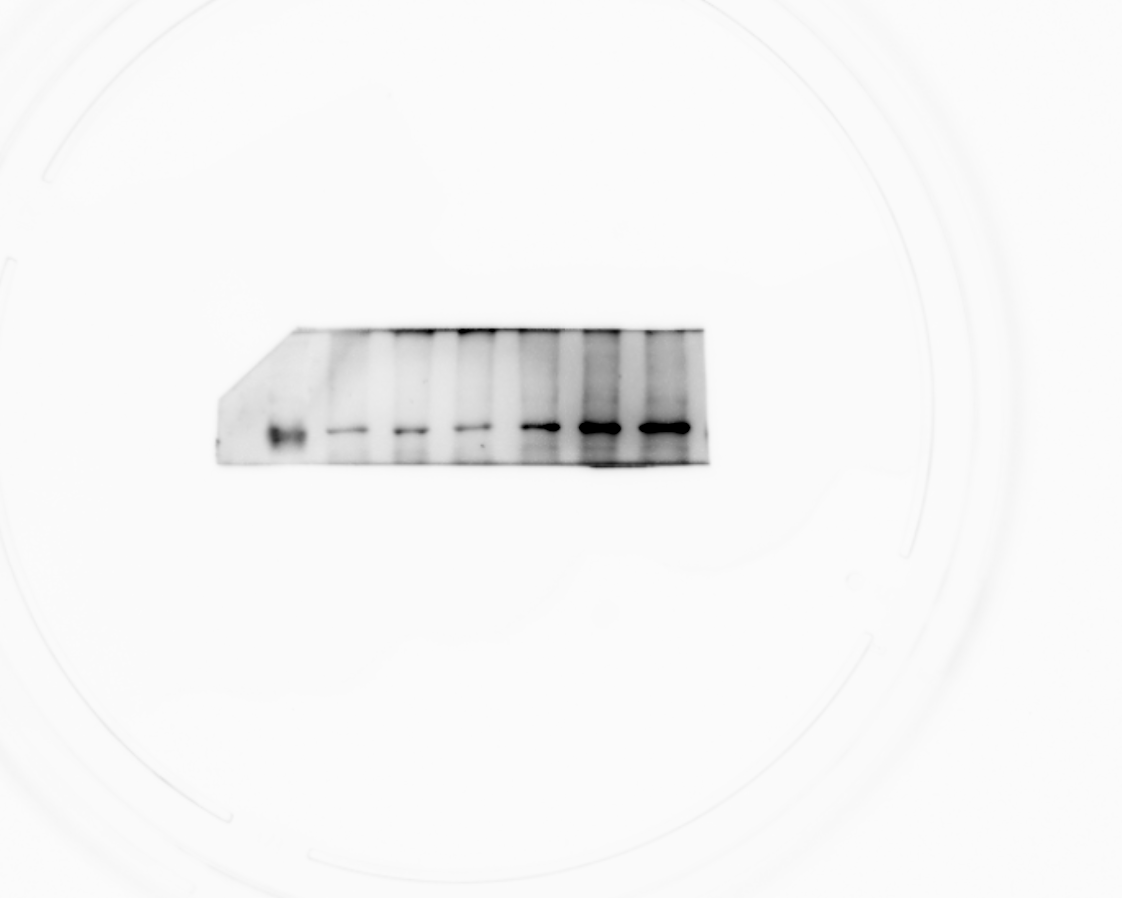

Supplement: Figure 4—source data 1. [file elife-100205-fig4-data1.zip › Figure 4-Source Data 1-Raw uncropped blots/Figure 4H/FLAG.tif]

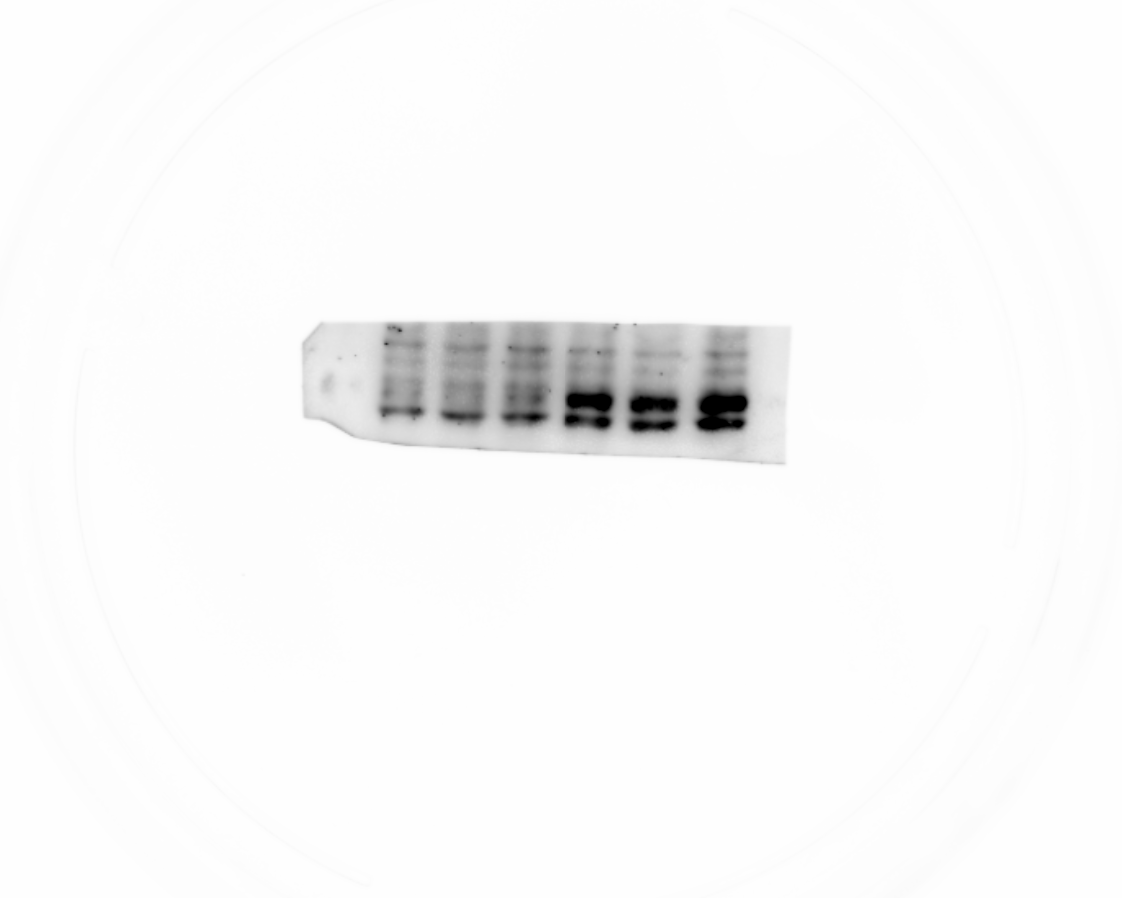

Supplement: Figure 4—source data 1. [file elife-100205-fig4-data1.zip › Figure 4-Source Data 1-Raw uncropped blots/Figure 4H/UCP1.tif]

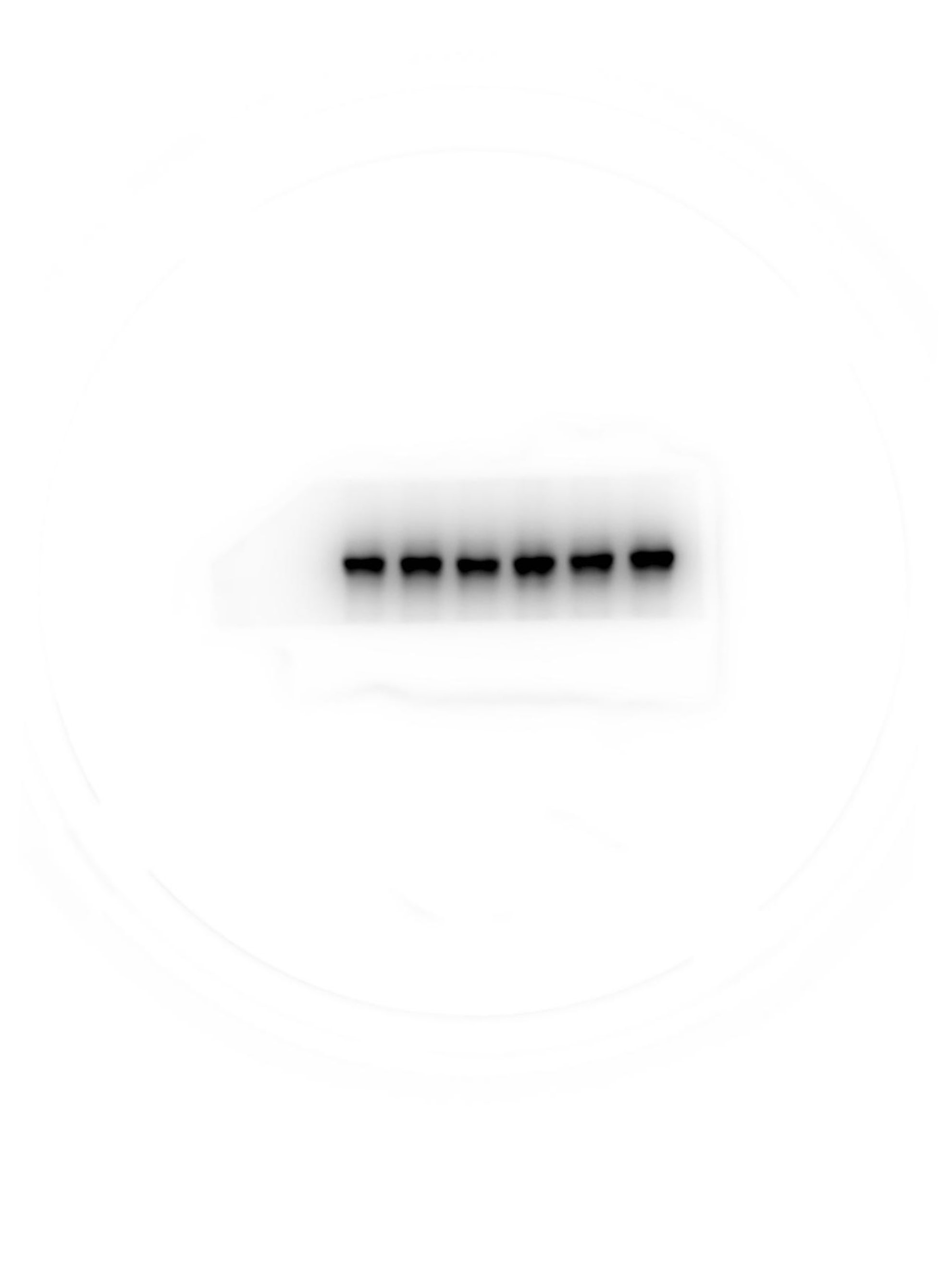

Supplement: Figure 4—source data 1. [file elife-100205-fig4-data1.zip › Figure 4-Source Data 1-Raw uncropped blots/Figure 4H/α-Tubulin.jpg]

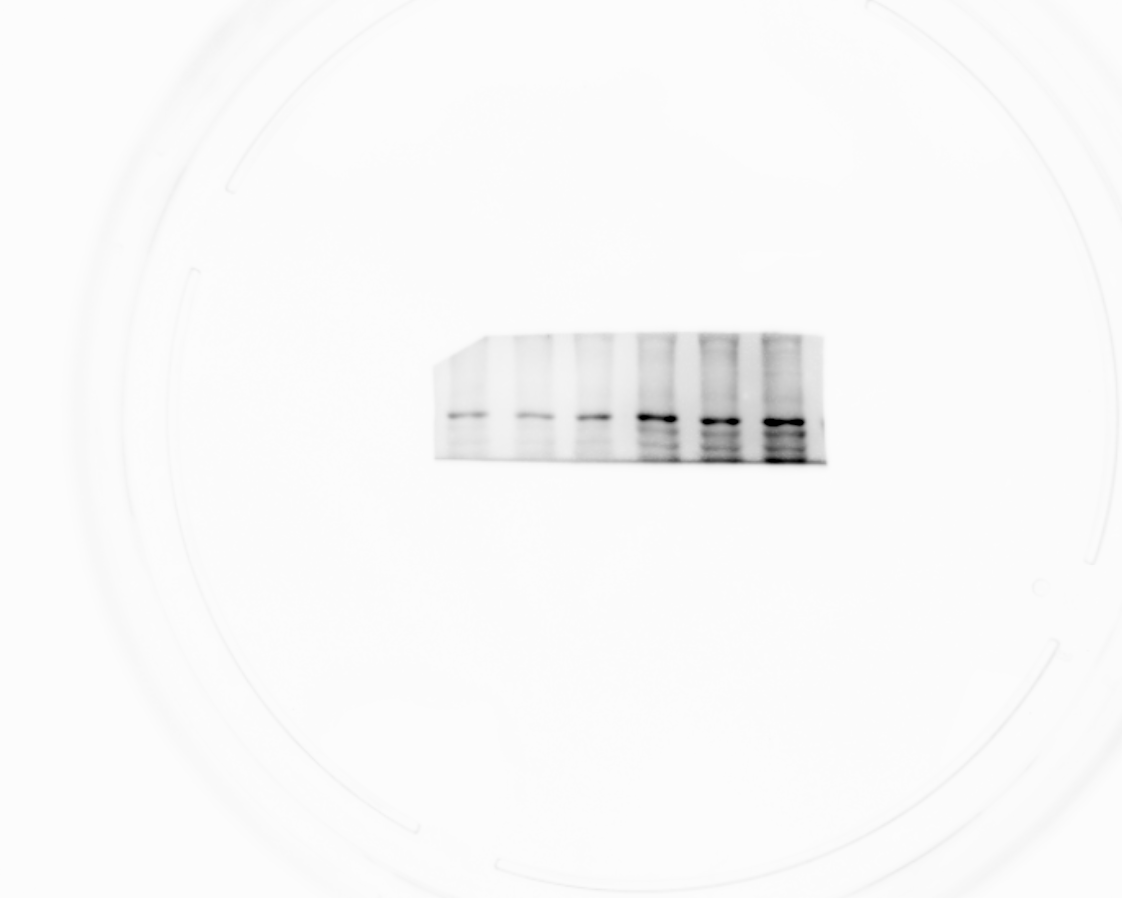

Supplement: Figure 4—source data 1. [file elife-100205-fig4-data1.zip › Figure 4-Source Data 1-Raw uncropped blots/Figure 4I/FLAG.tif]

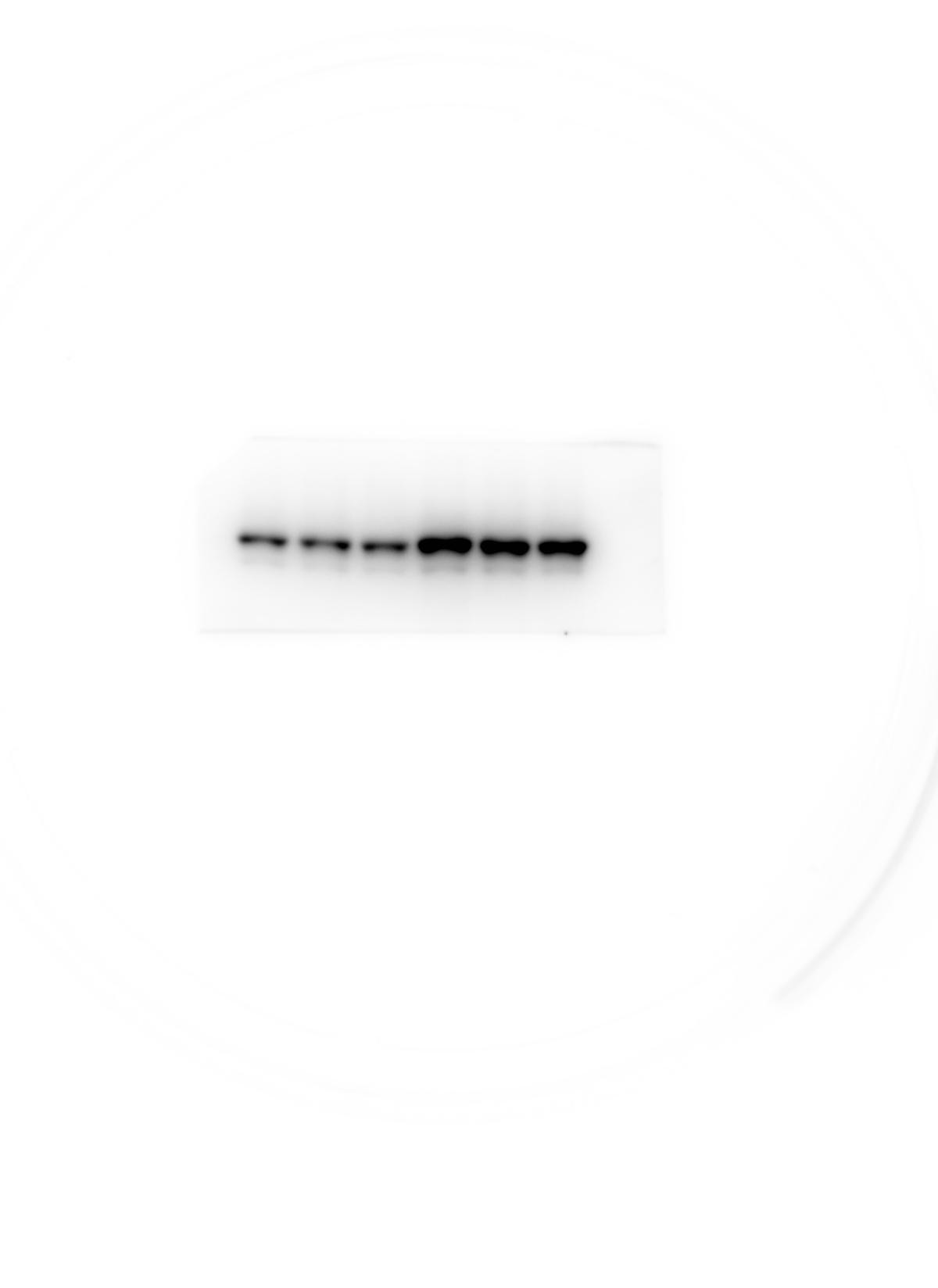

Supplement: Figure 4—source data 1. [file elife-100205-fig4-data1.zip › Figure 4-Source Data 1-Raw uncropped blots/Figure 4I/UCP1.jpg]

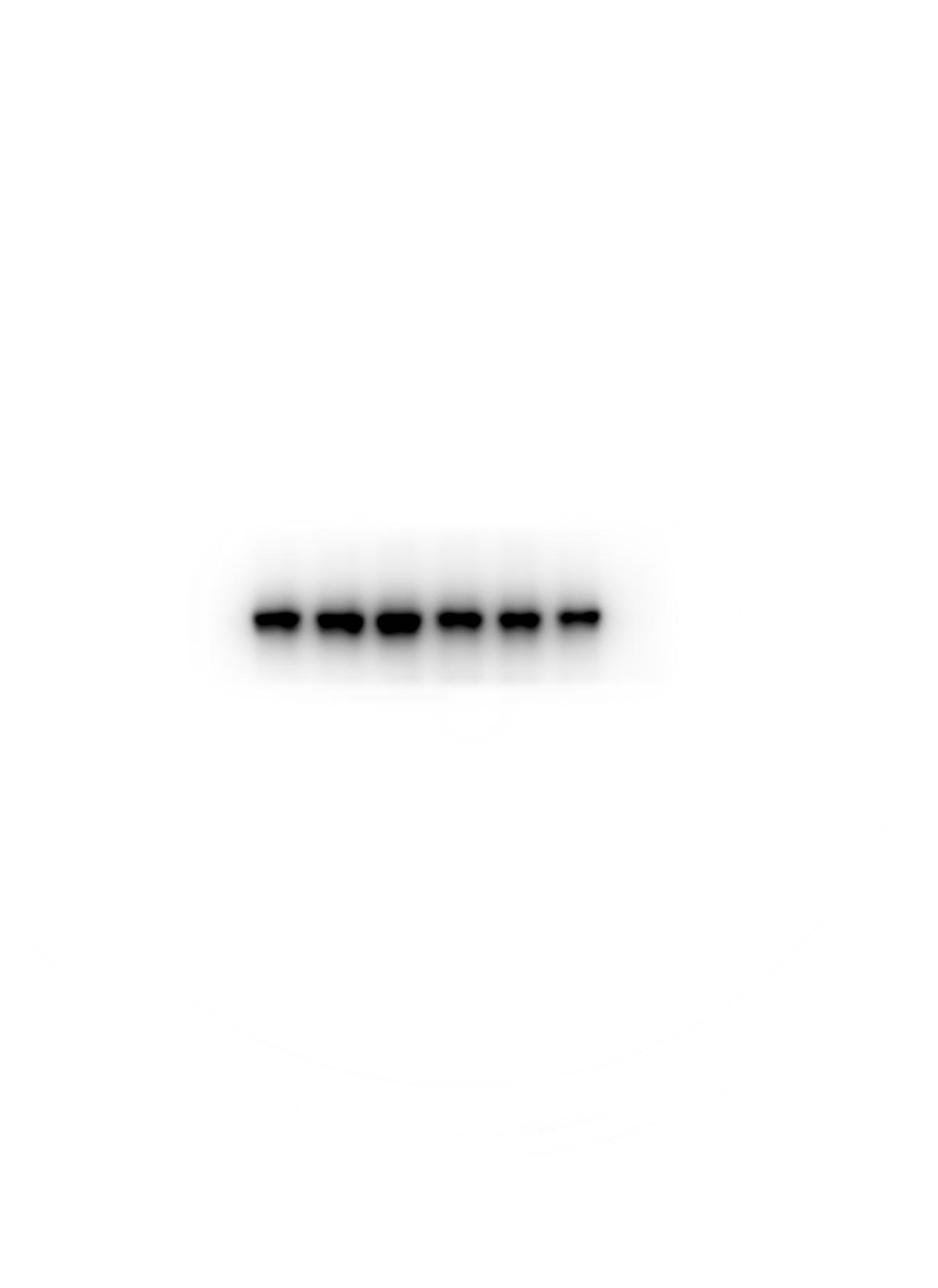

Supplement: Figure 4—source data 1. [file elife-100205-fig4-data1.zip › Figure 4-Source Data 1-Raw uncropped blots/Figure 4I/α-Tubulin.jpg]

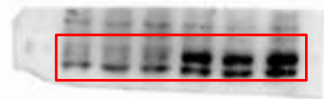

UCP1

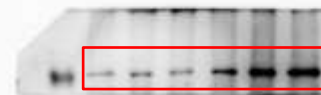

FLAG

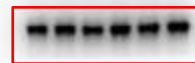

$\alpha$ -Tubulin

Supplement: Figure 4—source data 2. [file elife-100205-fig4-data2.zip › Figure 4-Source Data 2 -Uncropped and labeled blots/Figure 4H.pdf]

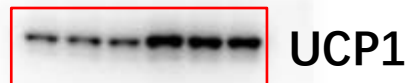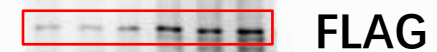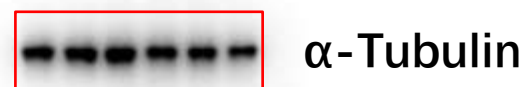

Supplement: Figure 4—source data 2. [file elife-100205-fig4-data2.zip › Figure 4-Source Data 2 -Uncropped and labeled blots/Figure 4I.pdf]

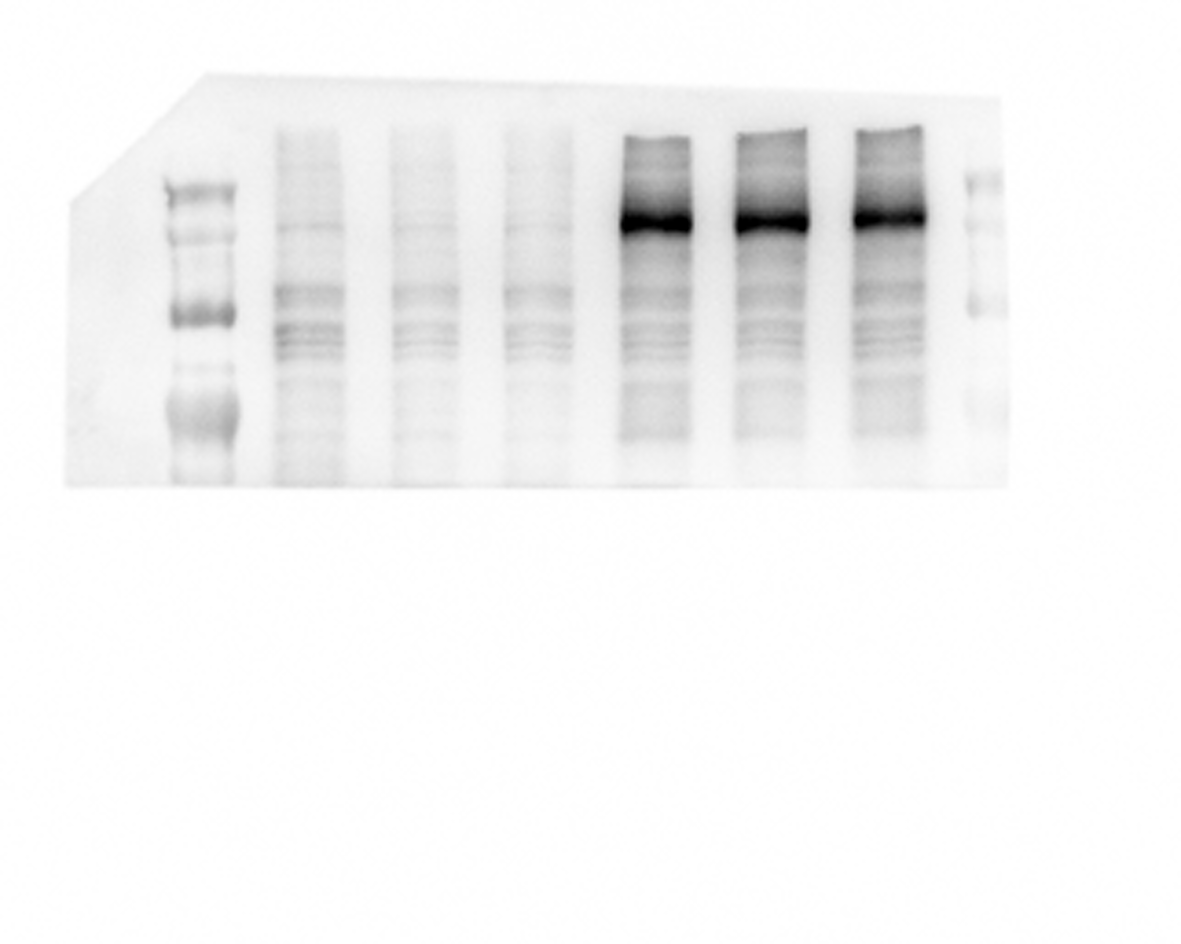

Supplement: Figure 4—figure supplement 1—source data 1. [file elife-100205-fig4-figsupp1-data1.zip › Figure 4-figure supplement 1-Source Data 1-Raw uncropped blots/Figure 4-figure supplement 1J/FLAG.tif]

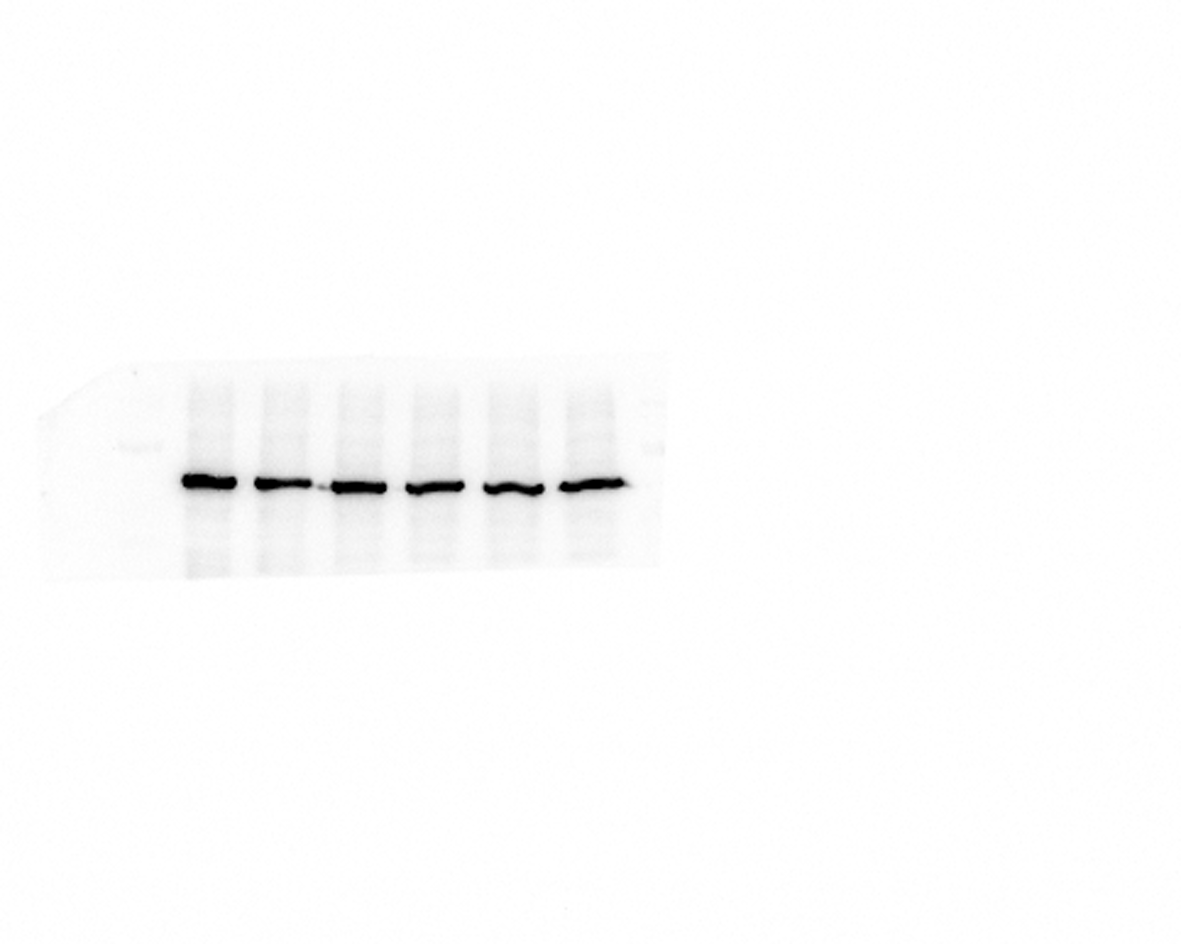

Supplement: Figure 4—figure supplement 1—source data 1. [file elife-100205-fig4-figsupp1-data1.zip › Figure 4-figure supplement 1-Source Data 1-Raw uncropped blots/Figure 4-figure supplement 1J/HSP90.tif]

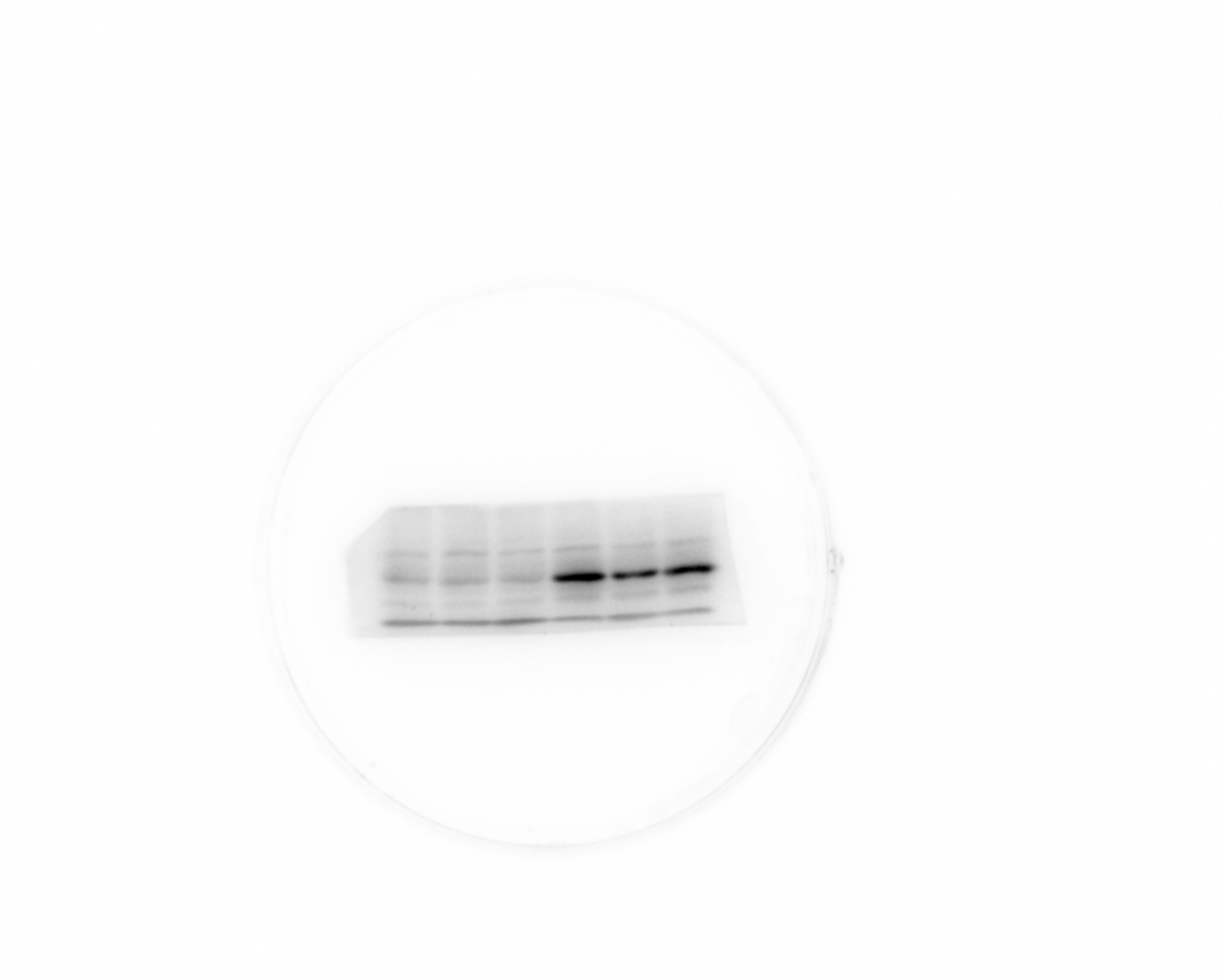

Supplement: Figure 4—figure supplement 1—source data 1. [file elife-100205-fig4-figsupp1-data1.zip › Figure 4-figure supplement 1-Source Data 1-Raw uncropped blots/Figure 4-figure supplement 1J/UCP1.tif]

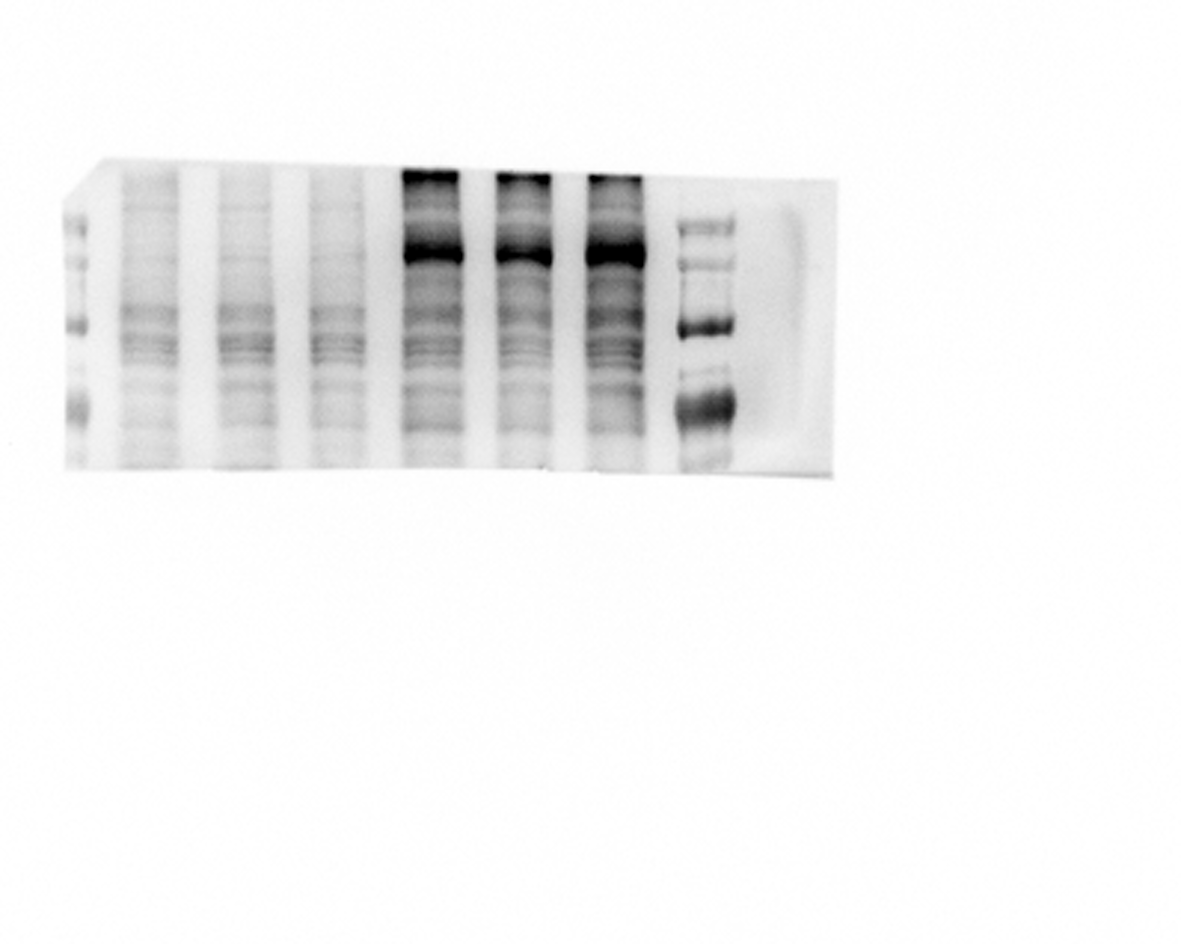

Supplement: Figure 4—figure supplement 1—source data 1. [file elife-100205-fig4-figsupp1-data1.zip › Figure 4-figure supplement 1-Source Data 1-Raw uncropped blots/Figure 4-figure supplement 1K/FLAG.tif]

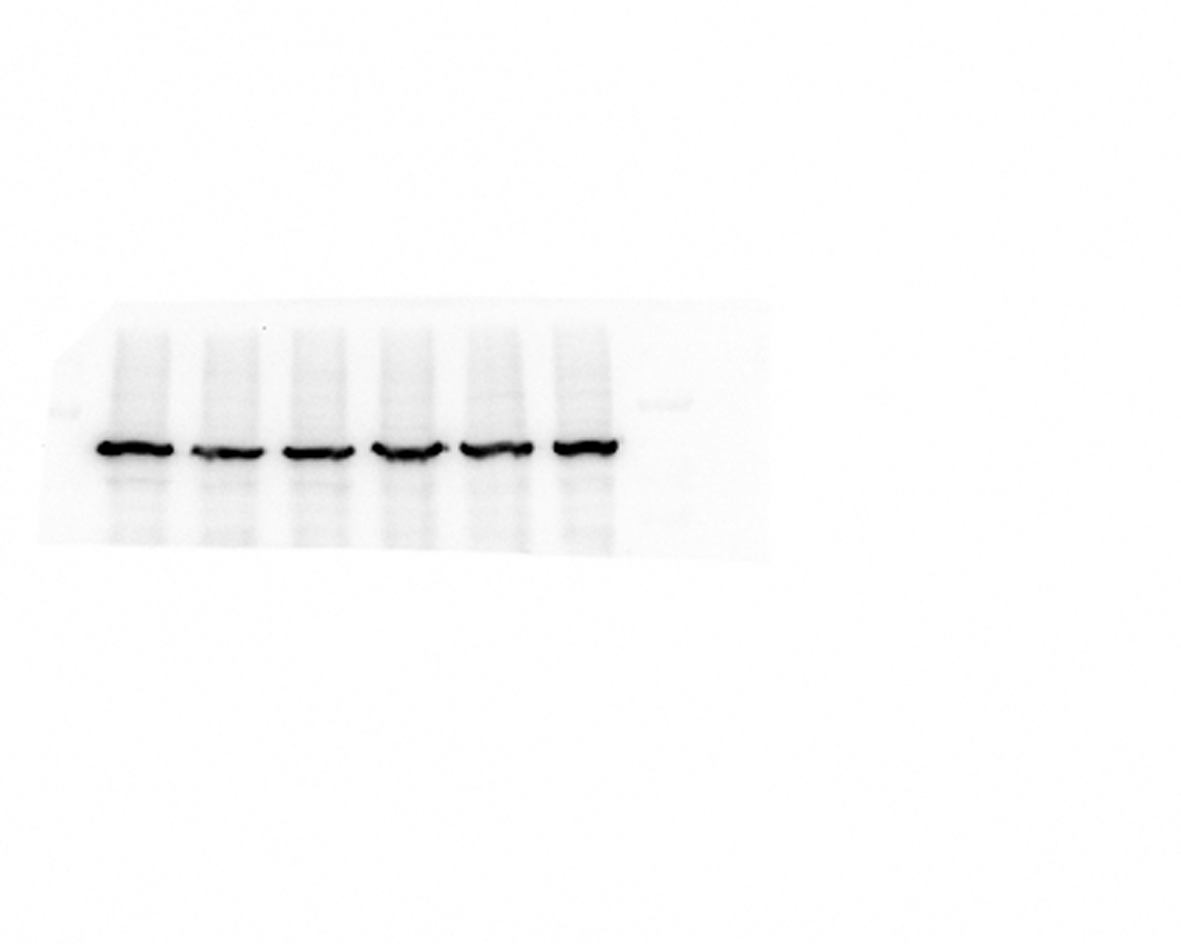

Supplement: Figure 4—figure supplement 1—source data 1. [file elife-100205-fig4-figsupp1-data1.zip › Figure 4-figure supplement 1-Source Data 1-Raw uncropped blots/Figure 4-figure supplement 1K/HSP90.tif]

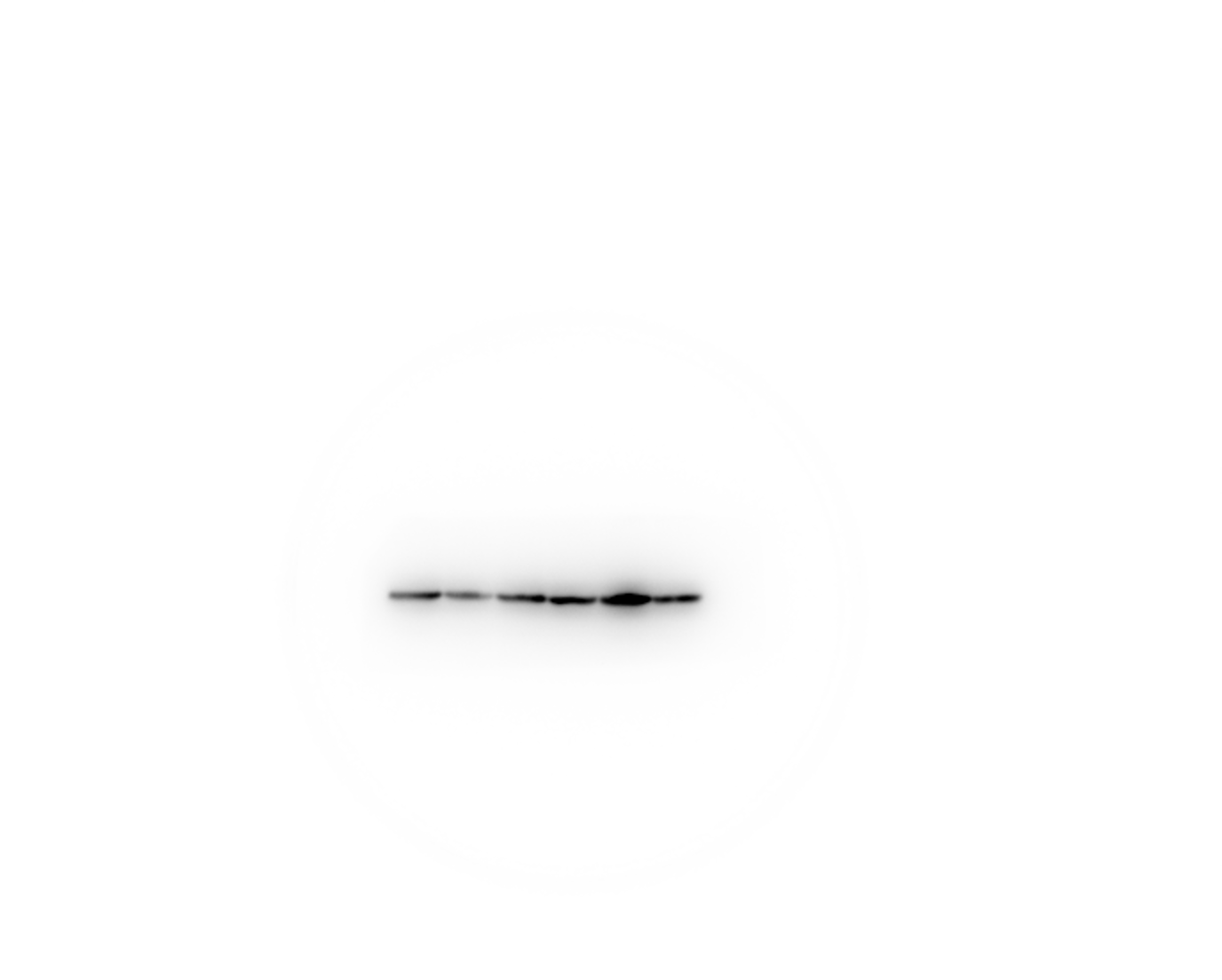

Supplement: Figure 4—figure supplement 1—source data 1. [file elife-100205-fig4-figsupp1-data1.zip › Figure 4-figure supplement 1-Source Data 1-Raw uncropped blots/Figure 4-figure supplement 1K/UCP1.tif]

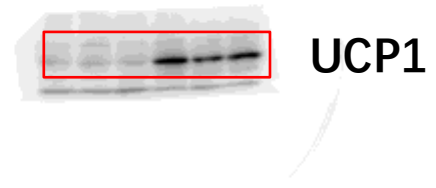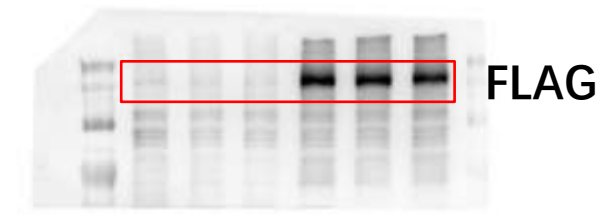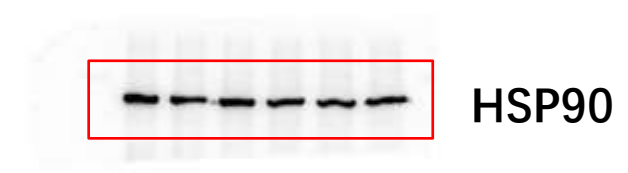

Supplement: Figure 4—figure supplement 1—source data 2. [file elife-100205-fig4-figsupp1-data2.zip › Figure 4-figure supplement 1-Source Data 2 -Uncropped and labeled blots/Figure 4-figure supplement 1J.pdf]

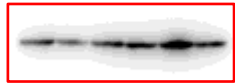

UCP1

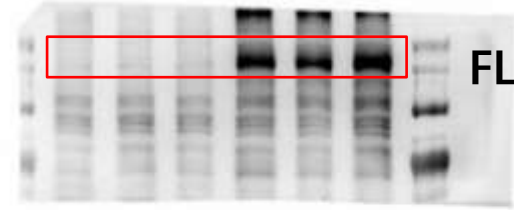

FLAG

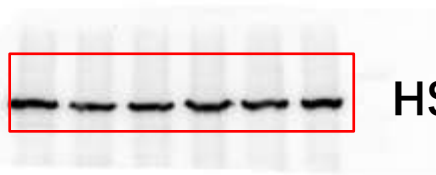

HSP90

Supplement: Figure 4—figure supplement 1—source data 2. [file elife-100205-fig4-figsupp1-data2.zip › Figure 4-figure supplement 1-Source Data 2 -Uncropped and labeled blots/Figure 4-figure supplement 1K.pdf]

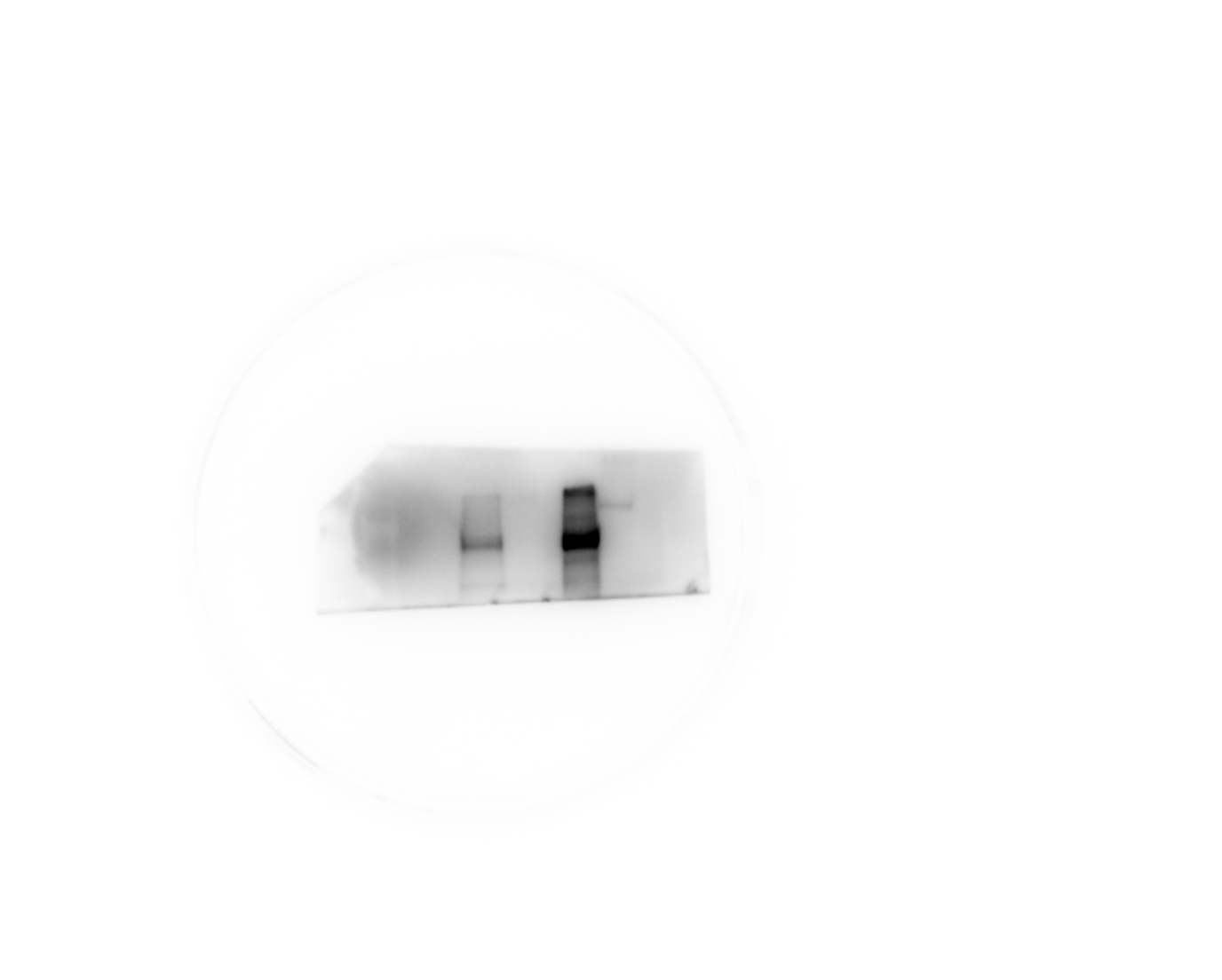

Supplement: Figure 5—source data 1. [file elife-100205-fig5-data1.zip › Figure 5-Source Data 1-Raw uncropped blots/Figure 5A/FLAG.tif]

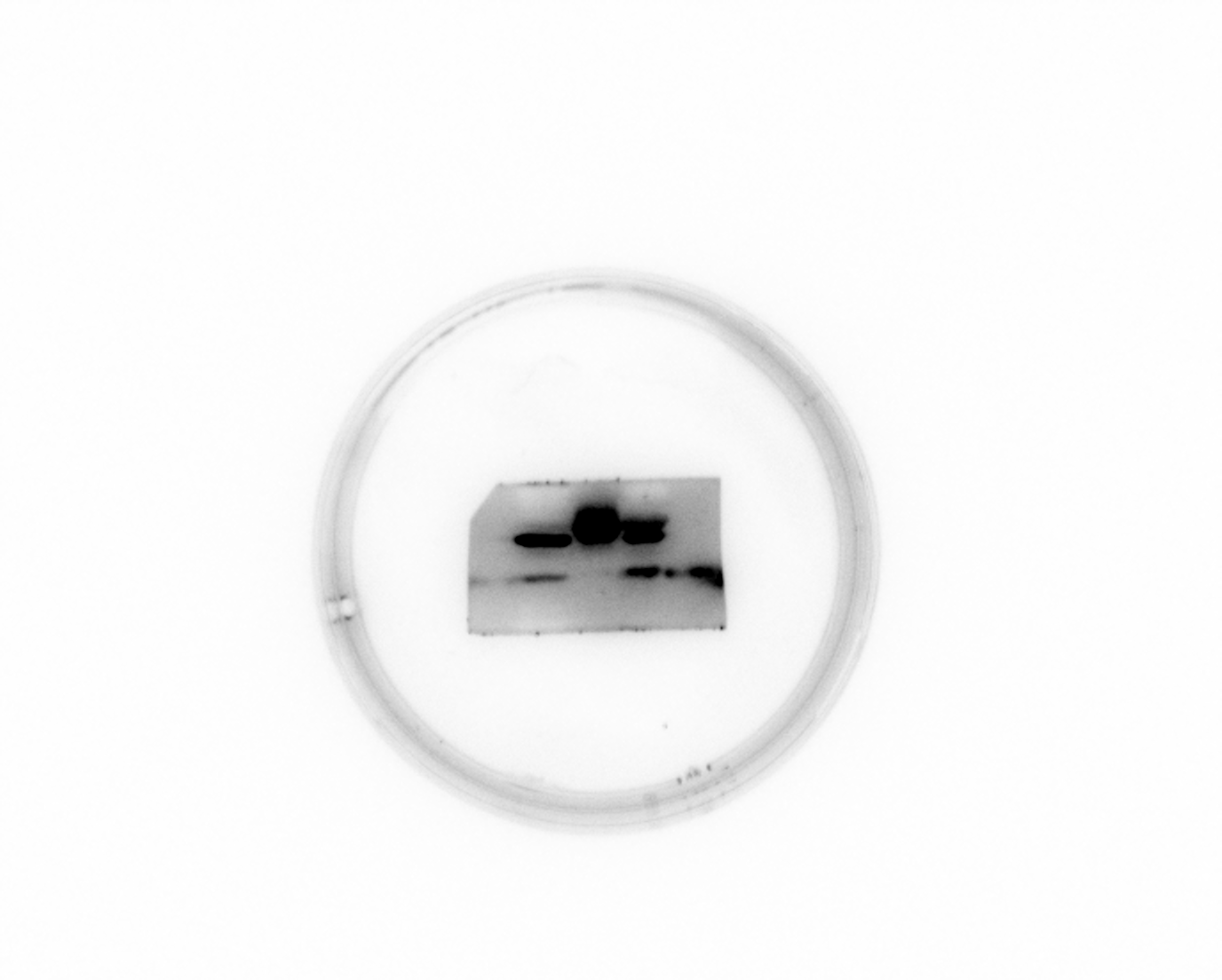

Supplement: Figure 5—source data 1. [file elife-100205-fig5-data1.zip › Figure 5-Source Data 1-Raw uncropped blots/Figure 5A/His.tif]

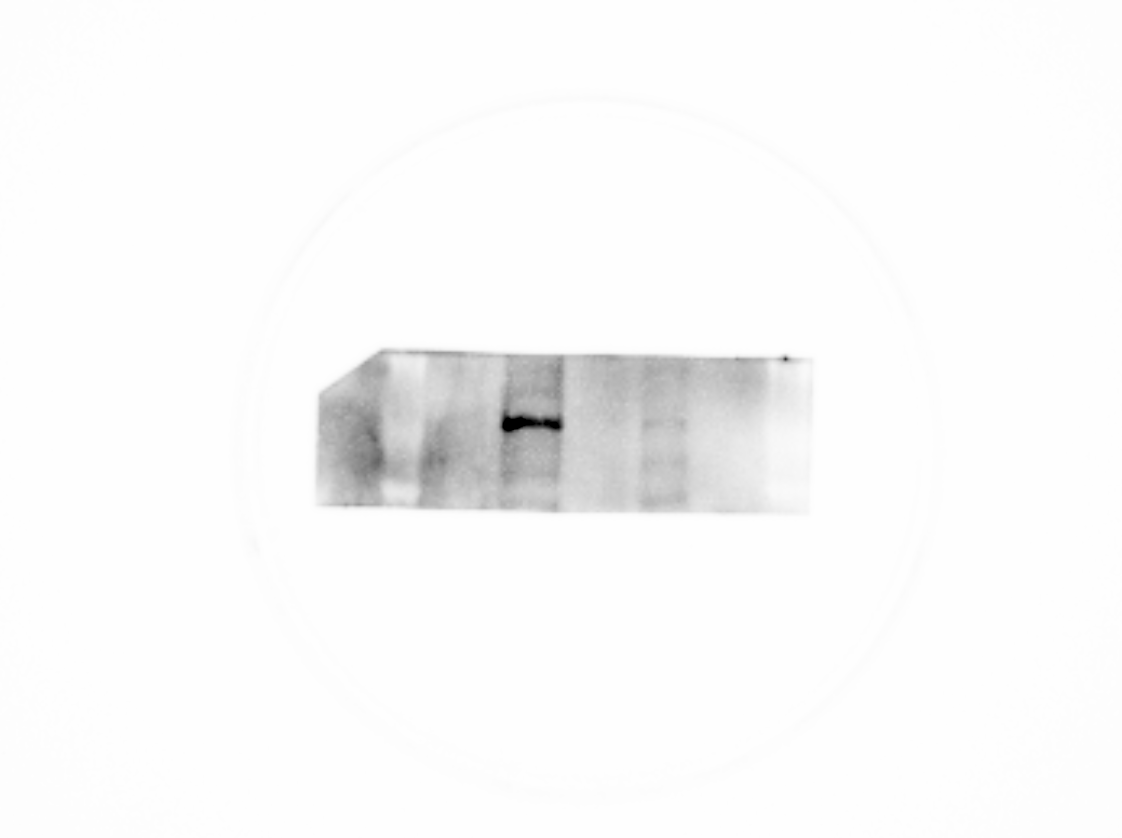

Supplement: Figure 5—source data 1. [file elife-100205-fig5-data1.zip › Figure 5-Source Data 1-Raw uncropped blots/Figure 5A/HSP90.tif]

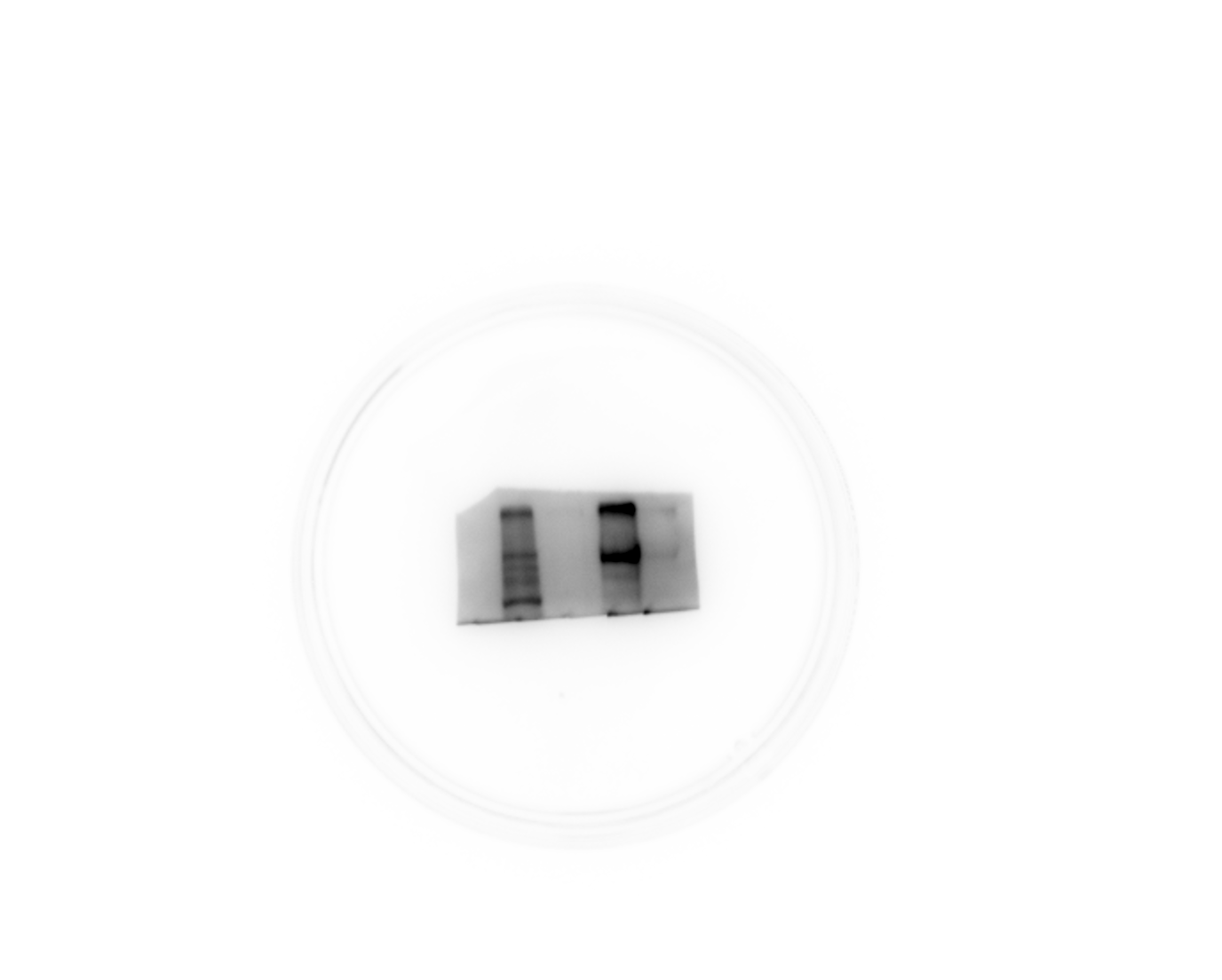

Supplement: Figure 5—source data 1. [file elife-100205-fig5-data1.zip › Figure 5-Source Data 1-Raw uncropped blots/Figure 5B/FLAG.tif]

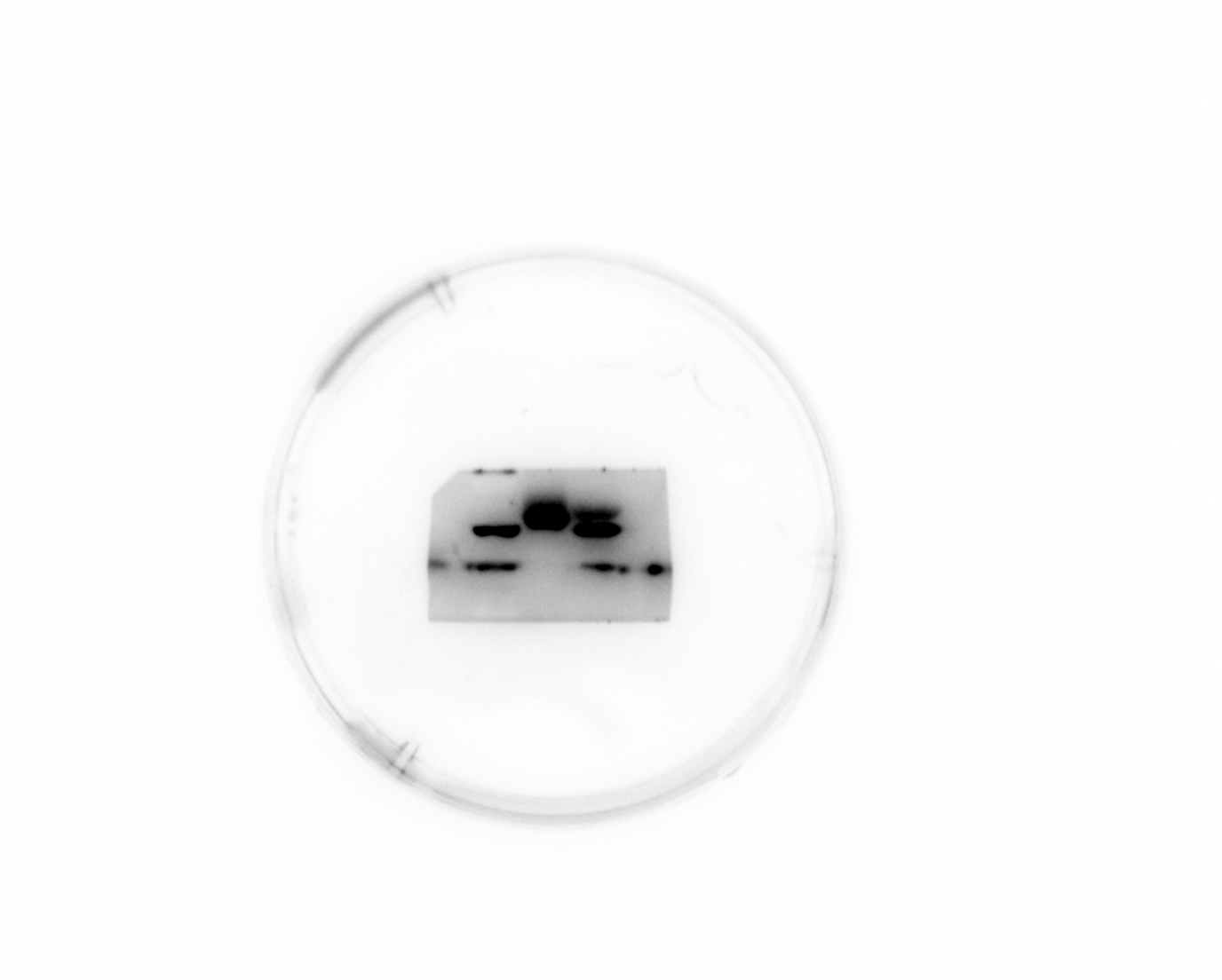

Supplement: Figure 5—source data 1. [file elife-100205-fig5-data1.zip › Figure 5-Source Data 1-Raw uncropped blots/Figure 5B/His.tif]

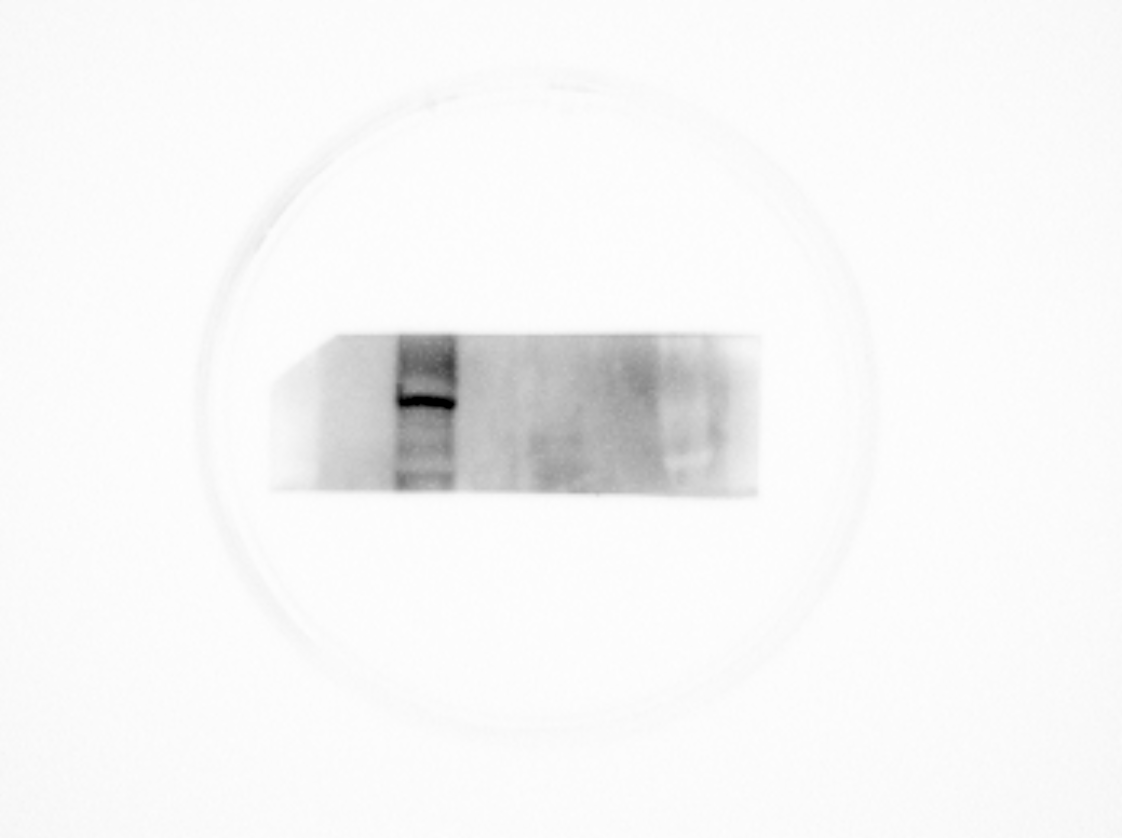

Supplement: Figure 5—source data 1. [file elife-100205-fig5-data1.zip › Figure 5-Source Data 1-Raw uncropped blots/Figure 5B/HSP90.tif]

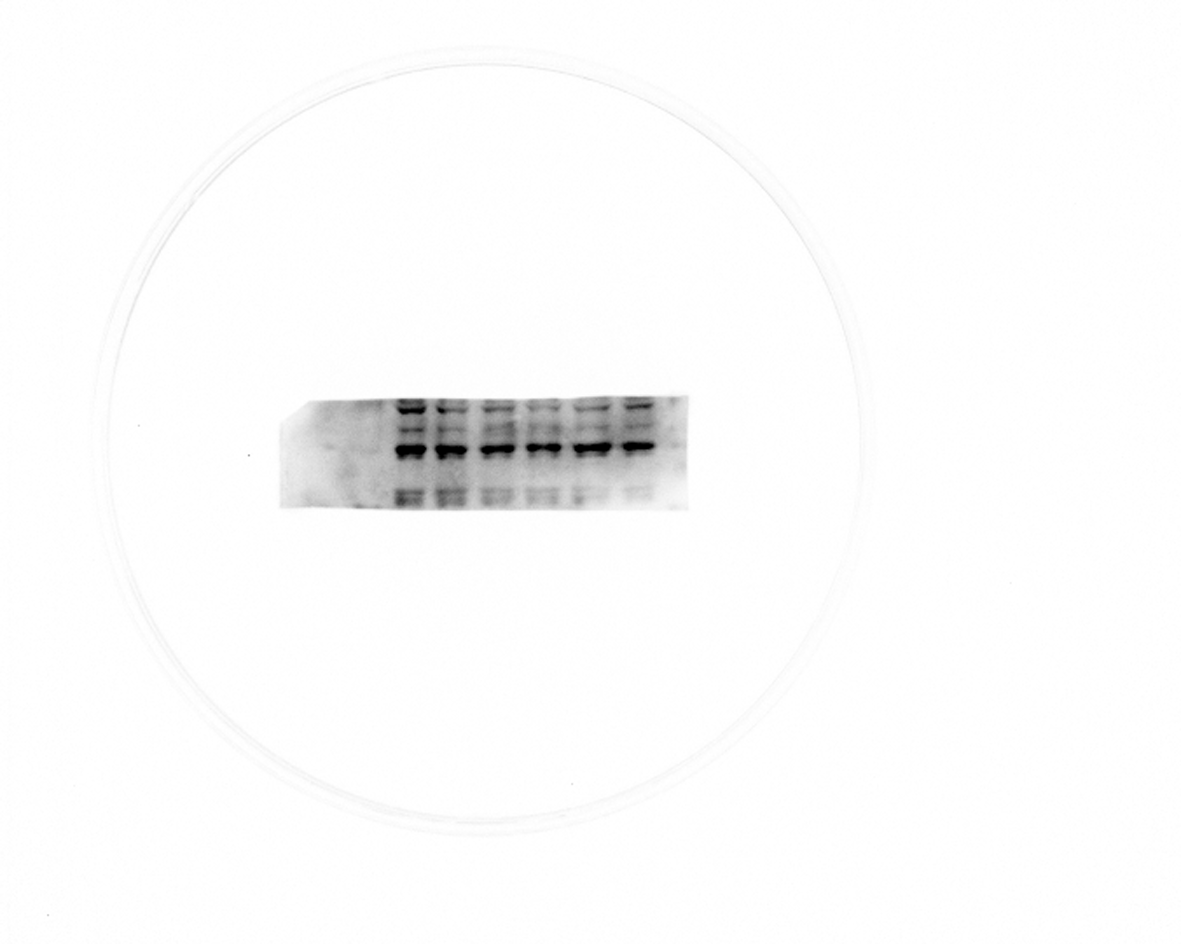

Supplement: Figure 5—source data 1. [file elife-100205-fig5-data1.zip › Figure 5-Source Data 1-Raw uncropped blots/Figure 5E/CREB.tif]

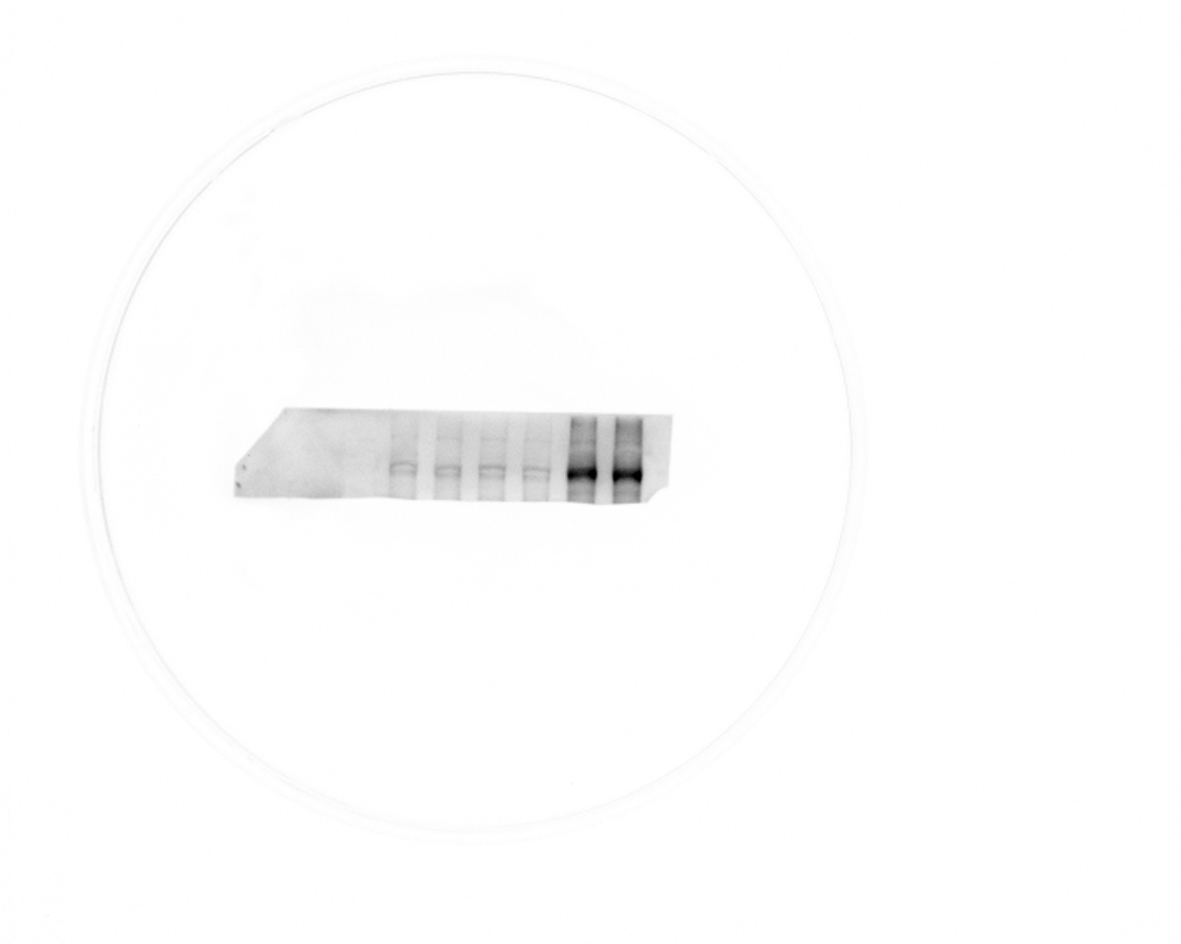

Supplement: Figure 5—source data 1. [file elife-100205-fig5-data1.zip › Figure 5-Source Data 1-Raw uncropped blots/Figure 5E/FLAG.tif]

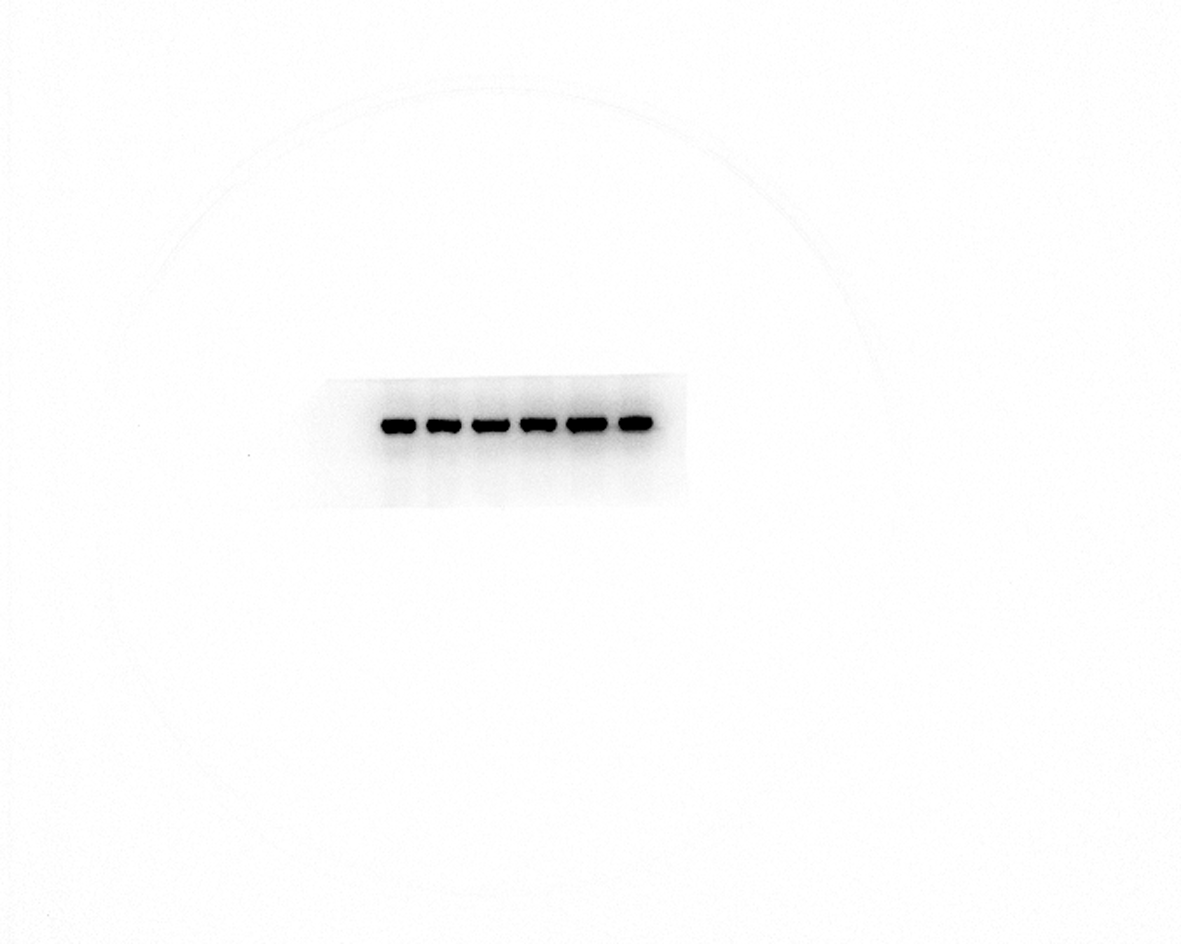

Supplement: Figure 5—source data 1. [file elife-100205-fig5-data1.zip › Figure 5-Source Data 1-Raw uncropped blots/Figure 5E/HSP90.tif]

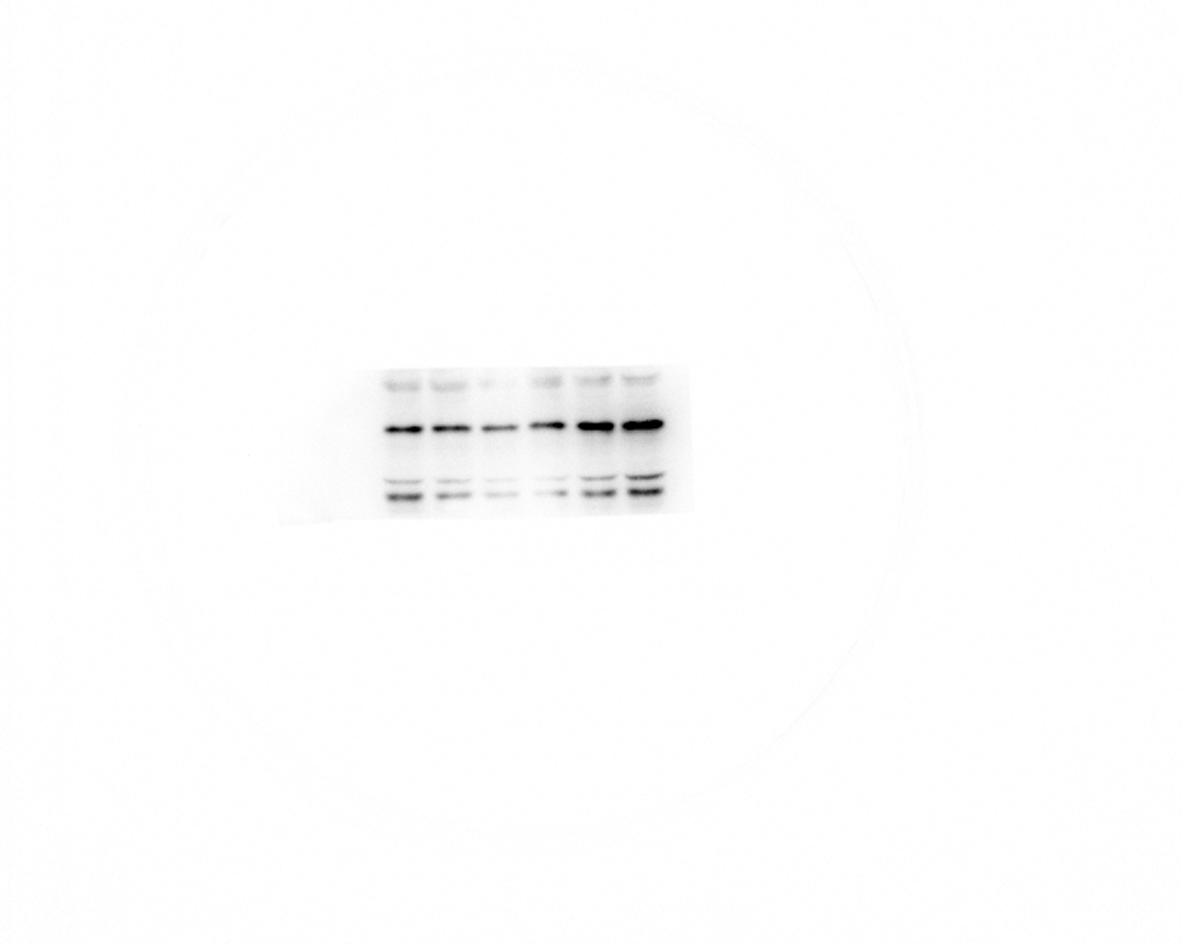

Supplement: Figure 5—source data 1. [file elife-100205-fig5-data1.zip › Figure 5-Source Data 1-Raw uncropped blots/Figure 5E/pCREB.tif]

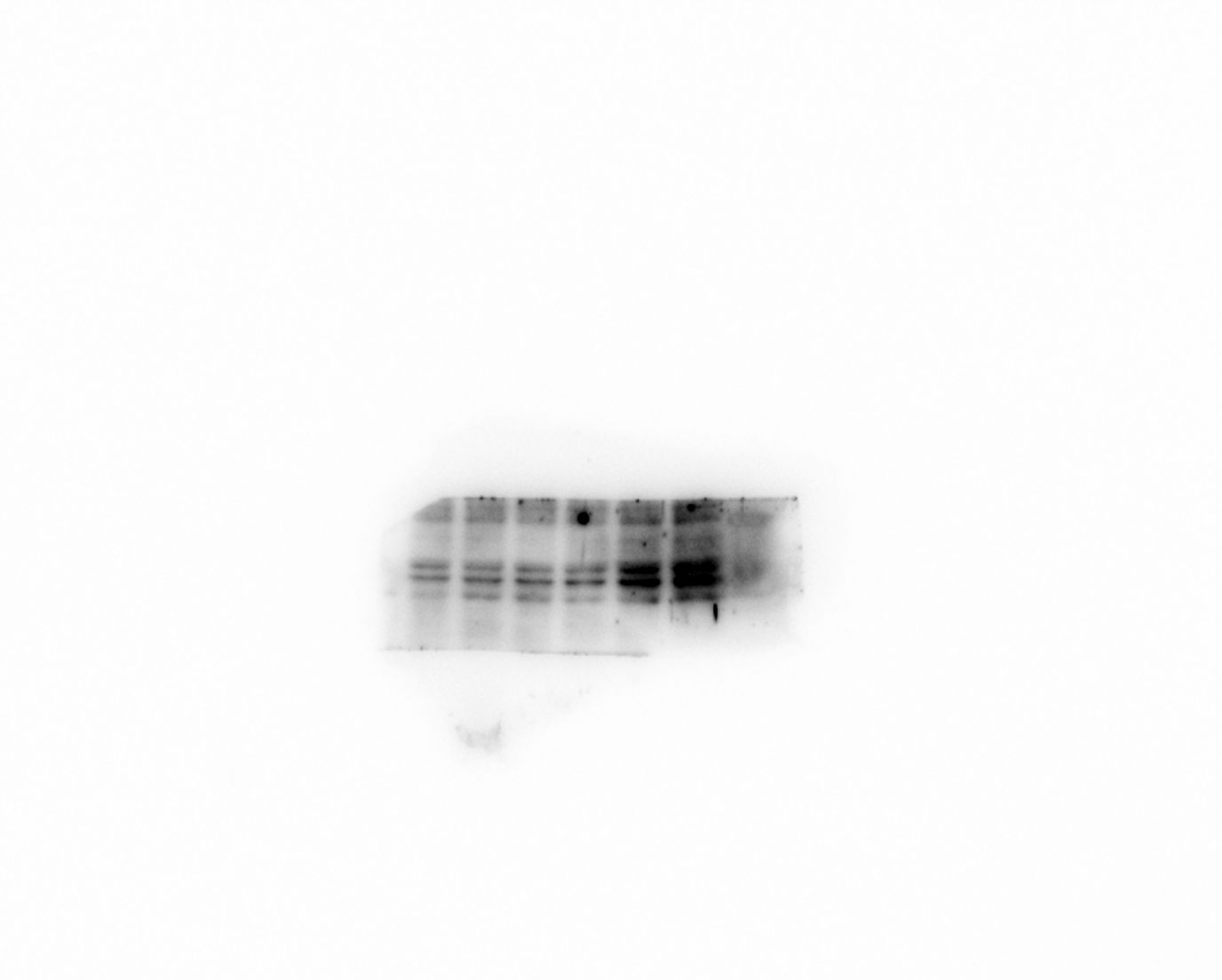

Supplement: Figure 5—source data 1. [file elife-100205-fig5-data1.zip › Figure 5-Source Data 1-Raw uncropped blots/Figure 5E/UCP1.tif]

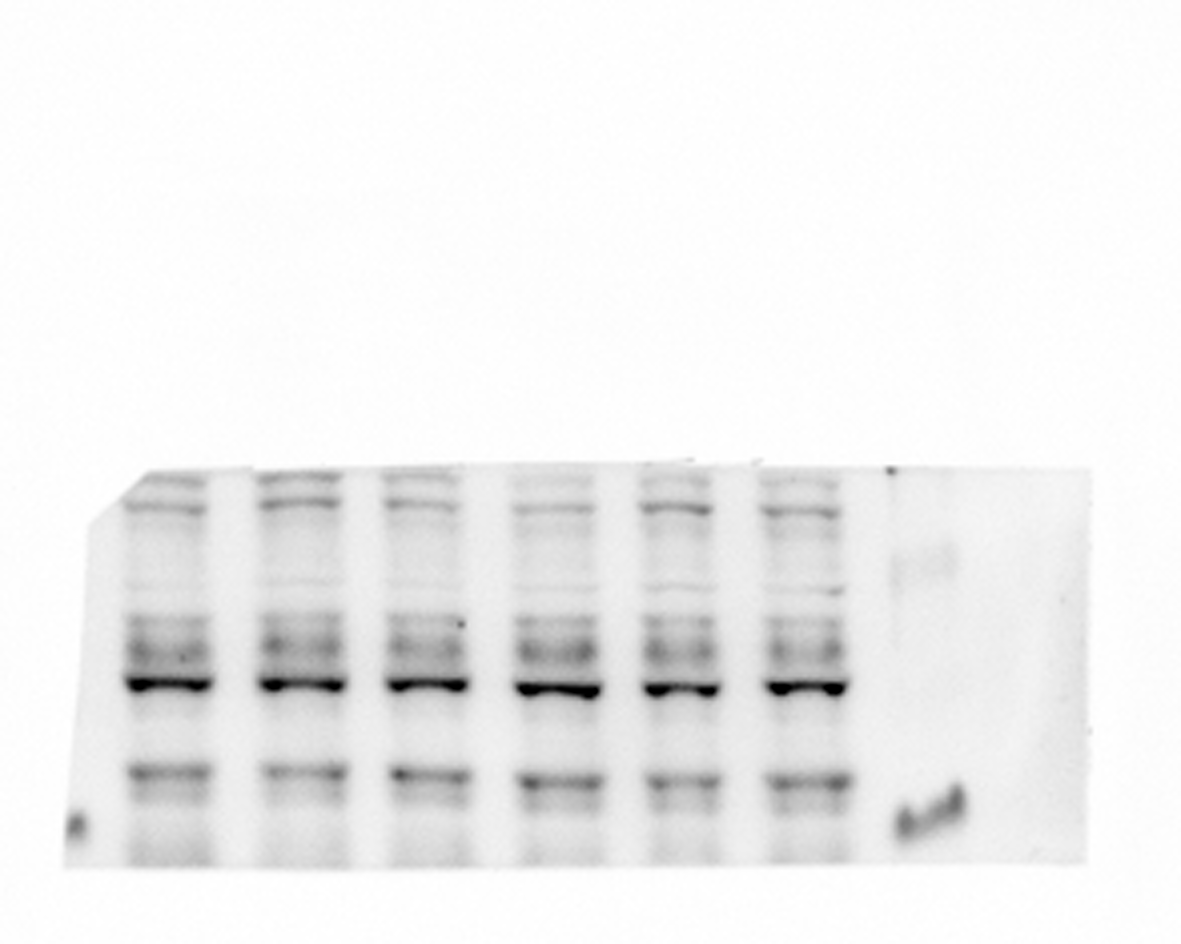

Supplement: Figure 5—source data 1. [file elife-100205-fig5-data1.zip › Figure 5-Source Data 1-Raw uncropped blots/Figure 5F/CREB-BAT.tif]

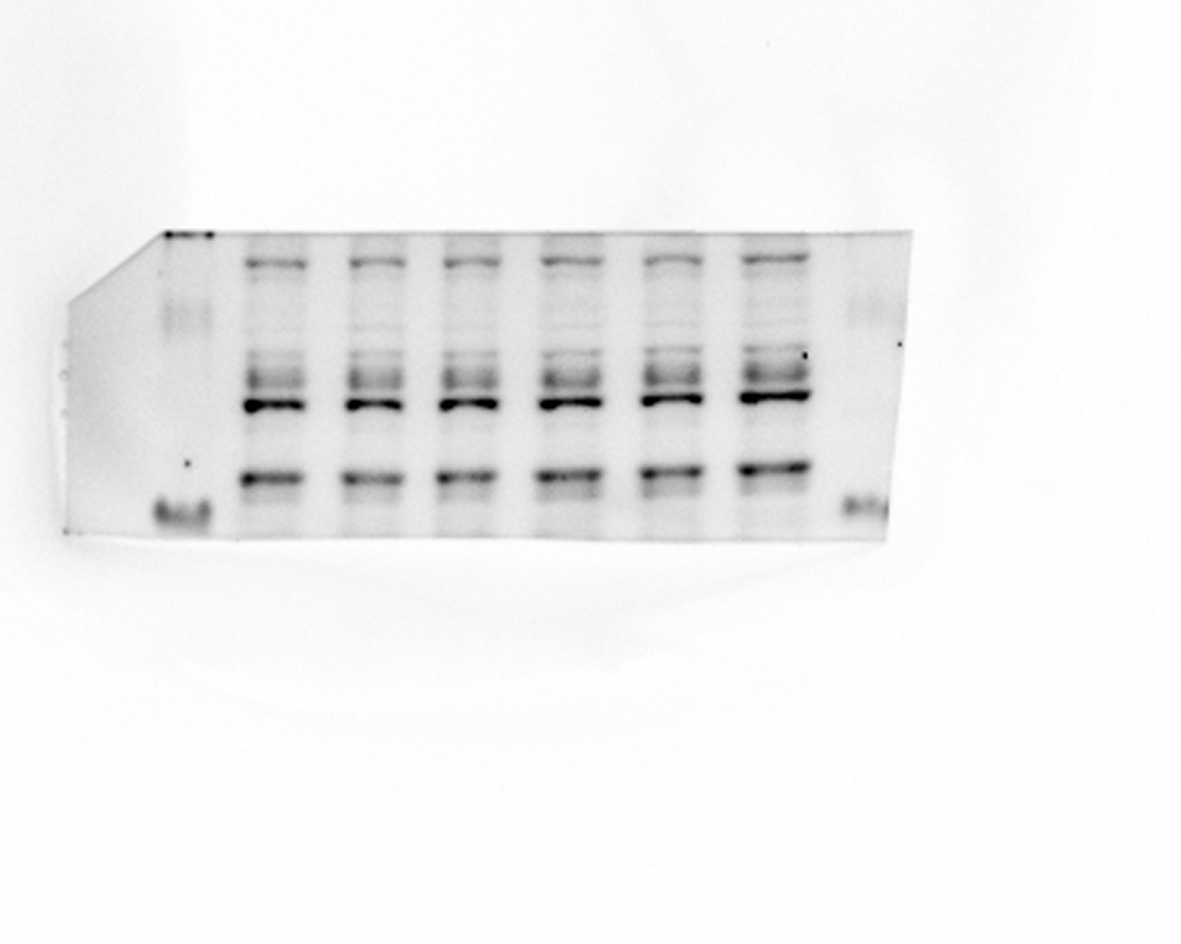

Supplement: Figure 5—source data 1. [file elife-100205-fig5-data1.zip › Figure 5-Source Data 1-Raw uncropped blots/Figure 5F/CREB-iWAT.tif]

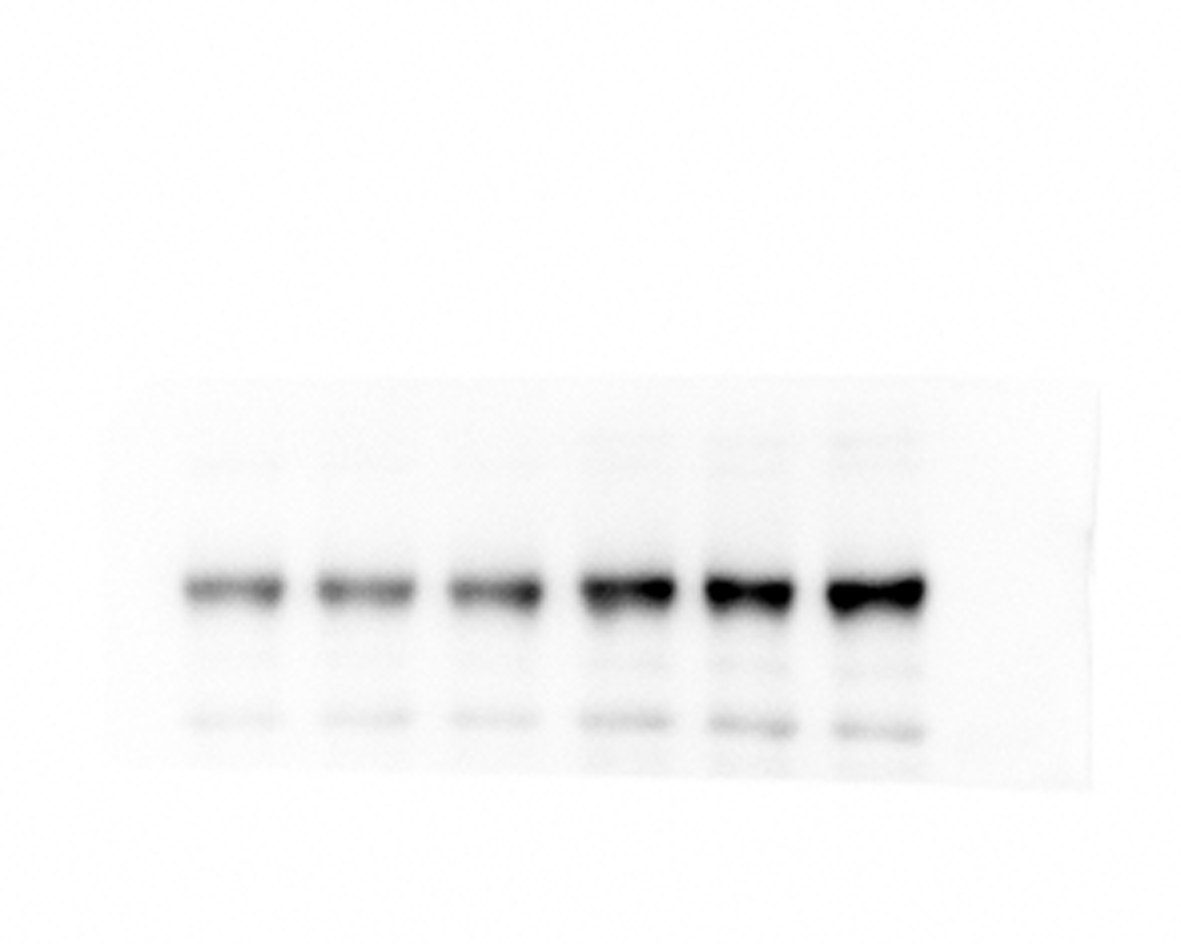

Supplement: Figure 5—source data 1. [file elife-100205-fig5-data1.zip › Figure 5-Source Data 1-Raw uncropped blots/Figure 5F/pCREB-BAT.tif]

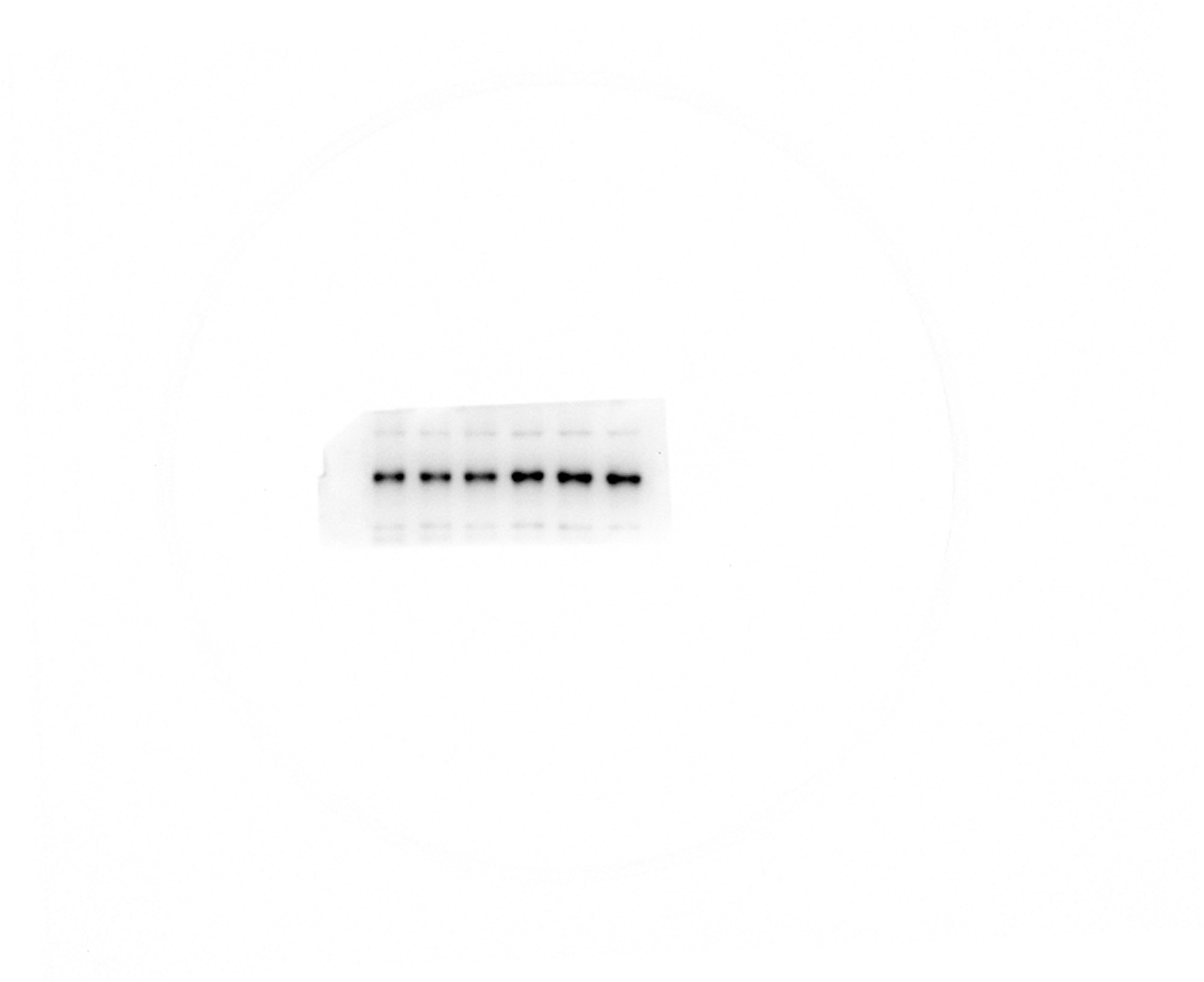

Supplement: Figure 5—source data 1. [file elife-100205-fig5-data1.zip › Figure 5-Source Data 1-Raw uncropped blots/Figure 5F/pCREB-iWAT.tif]

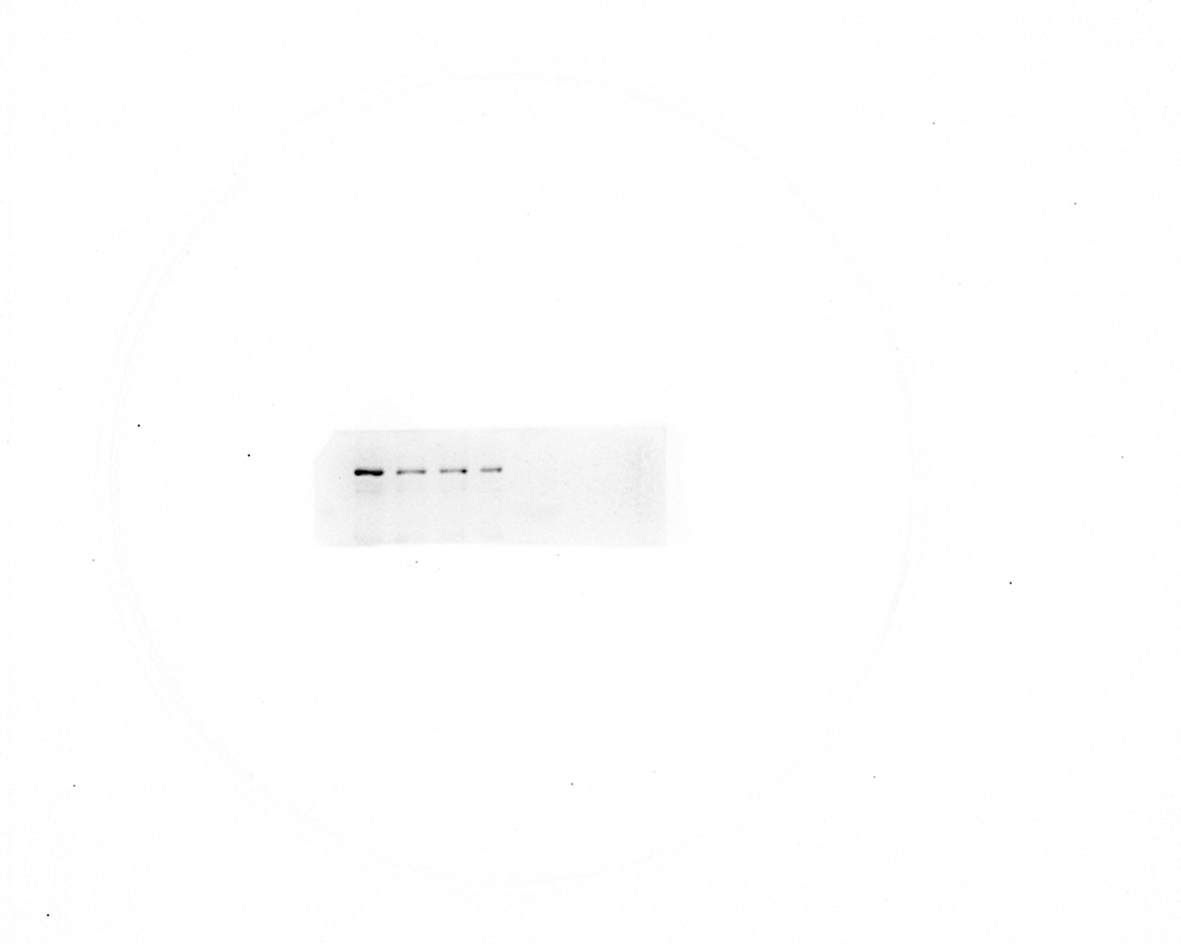

Supplement: Figure 5—source data 1. [file elife-100205-fig5-data1.zip › Figure 5-Source Data 1-Raw uncropped blots/Figure 5G/ADGRA3.tif]

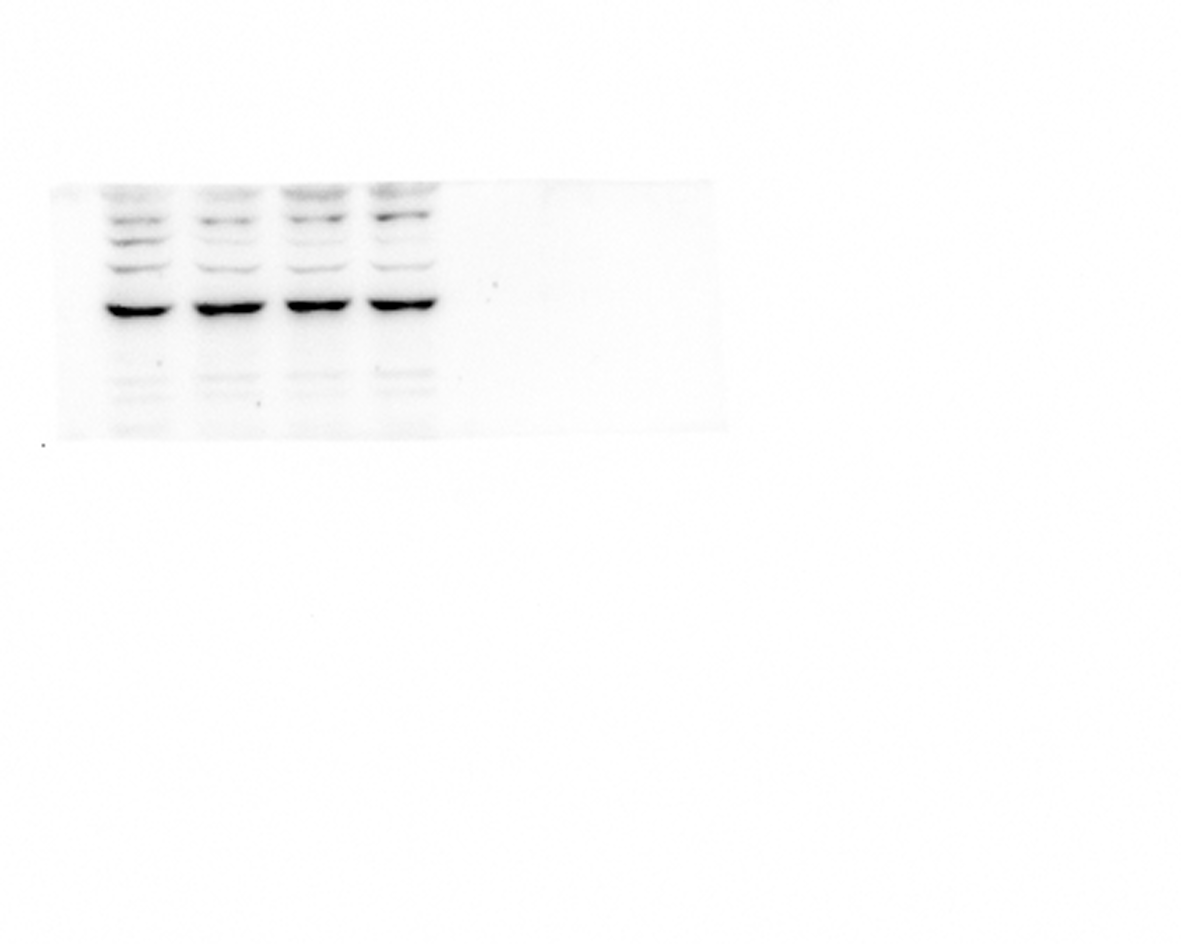

Supplement: Figure 5—source data 1. [file elife-100205-fig5-data1.zip › Figure 5-Source Data 1-Raw uncropped blots/Figure 5G/CREB.tif]

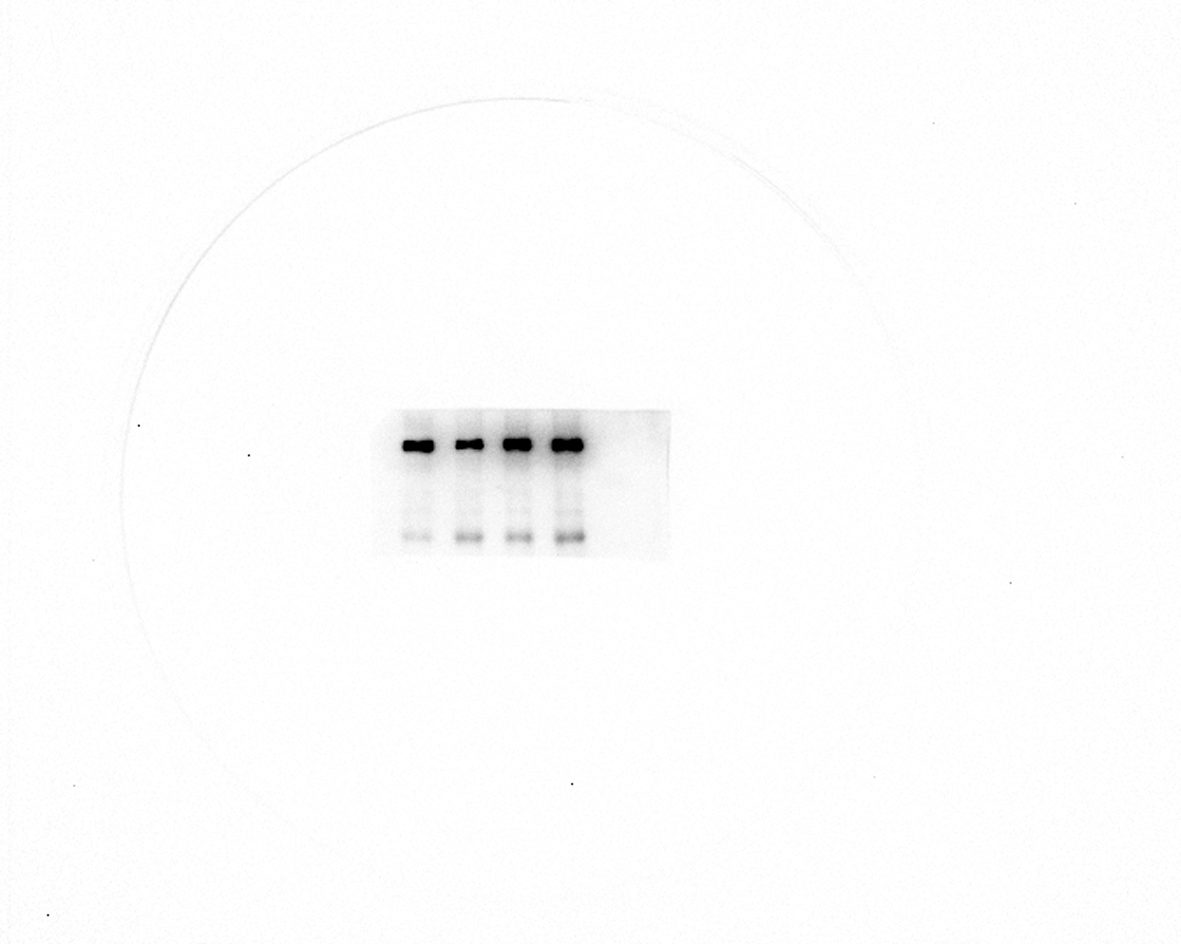

Supplement: Figure 5—source data 1. [file elife-100205-fig5-data1.zip › Figure 5-Source Data 1-Raw uncropped blots/Figure 5G/HSP90.tif]

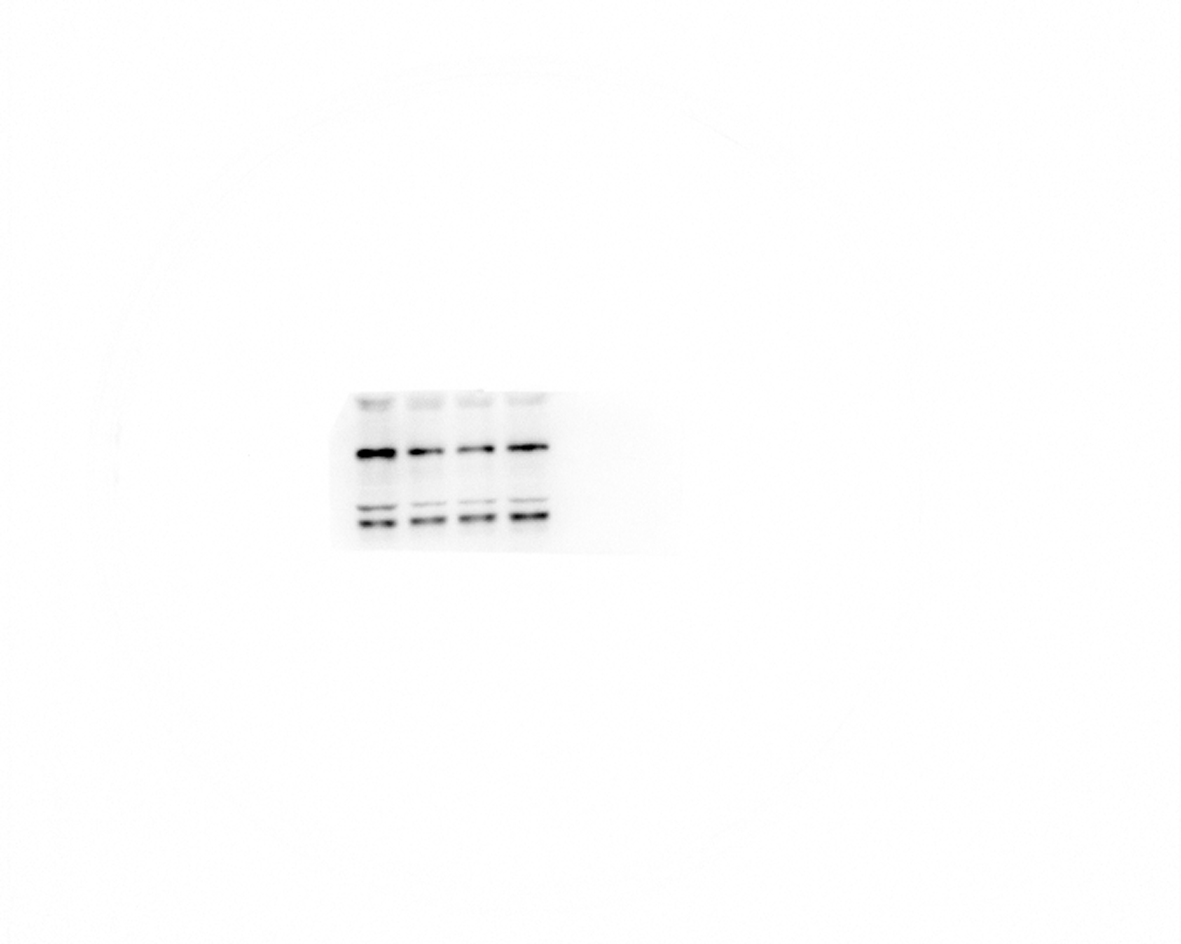

Supplement: Figure 5—source data 1. [file elife-100205-fig5-data1.zip › Figure 5-Source Data 1-Raw uncropped blots/Figure 5G/pCREB.tif]

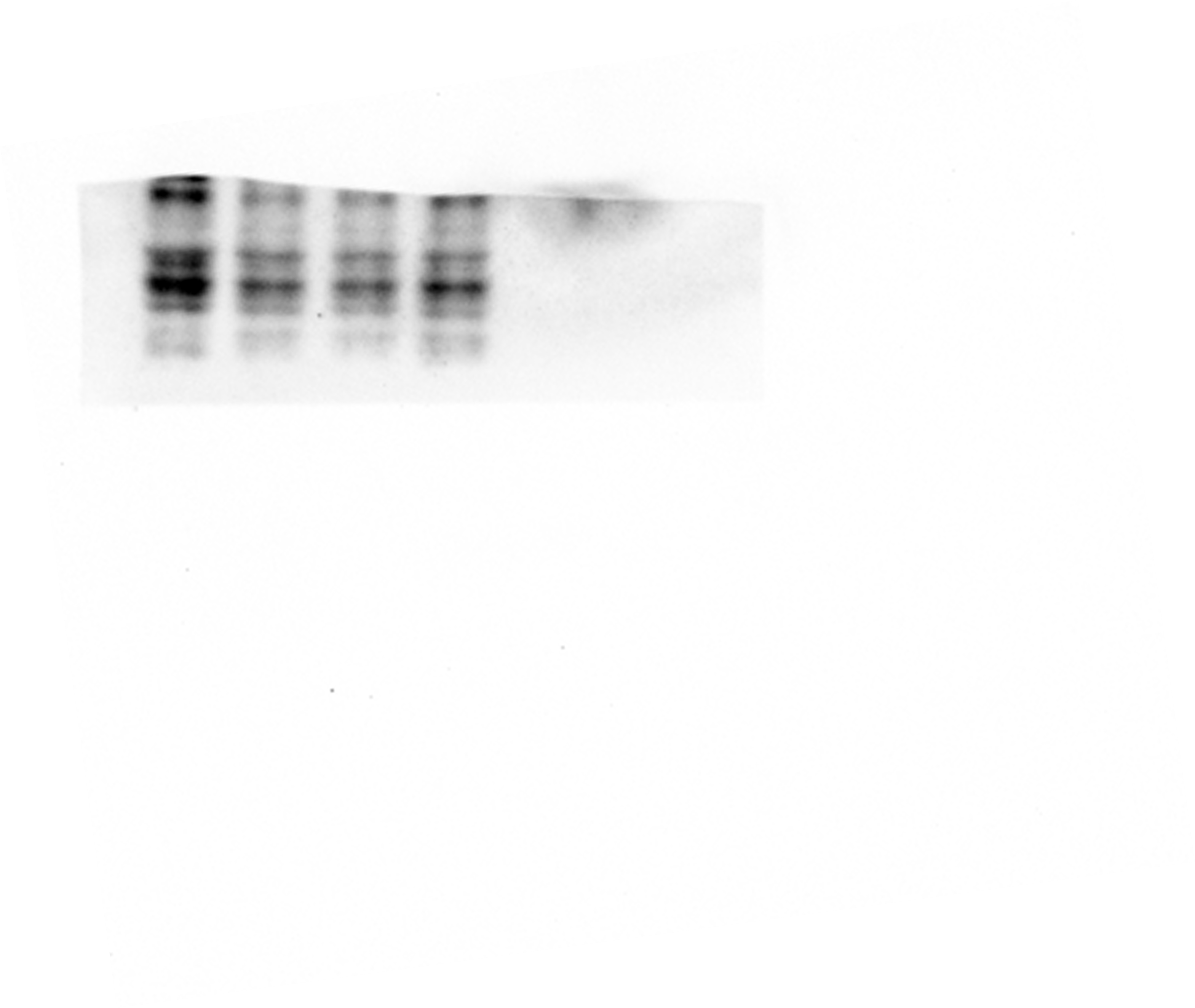

Supplement: Figure 5—source data 1. [file elife-100205-fig5-data1.zip › Figure 5-Source Data 1-Raw uncropped blots/Figure 5G/UCP1.tif]

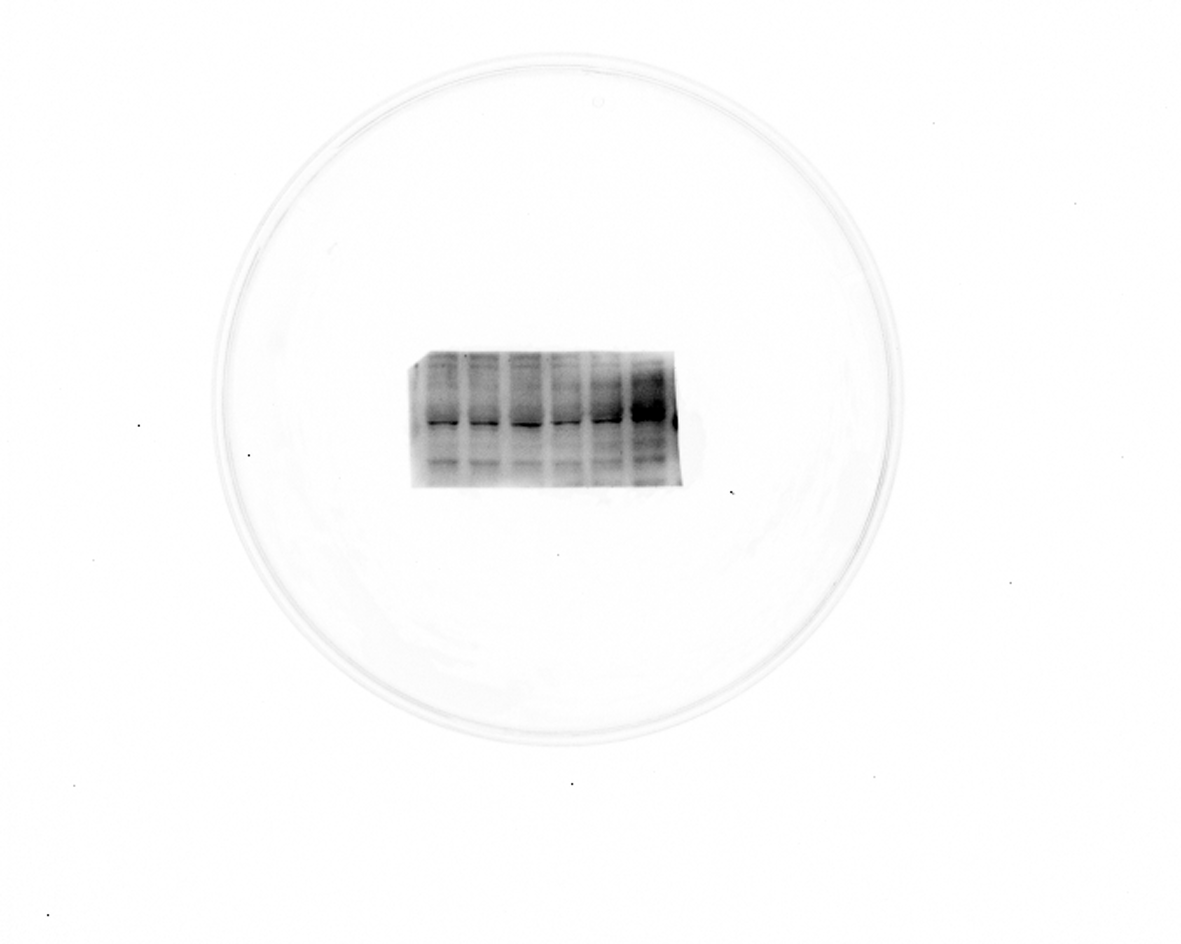

Supplement: Figure 5—source data 1. [file elife-100205-fig5-data1.zip › Figure 5-Source Data 1-Raw uncropped blots/Figure 5H/CREB-BAT.tif]

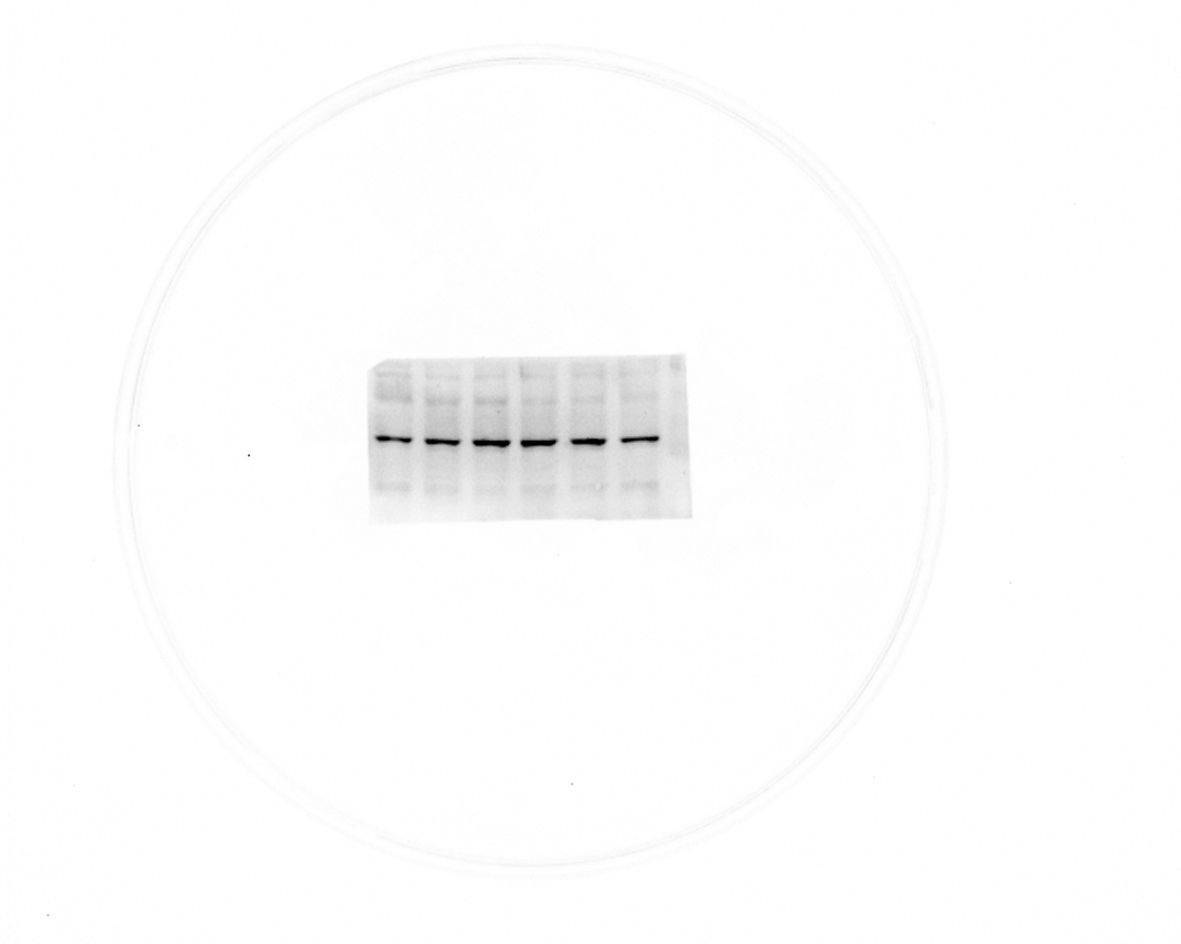

Supplement: Figure 5—source data 1. [file elife-100205-fig5-data1.zip › Figure 5-Source Data 1-Raw uncropped blots/Figure 5H/CREB-iWAT.tif]

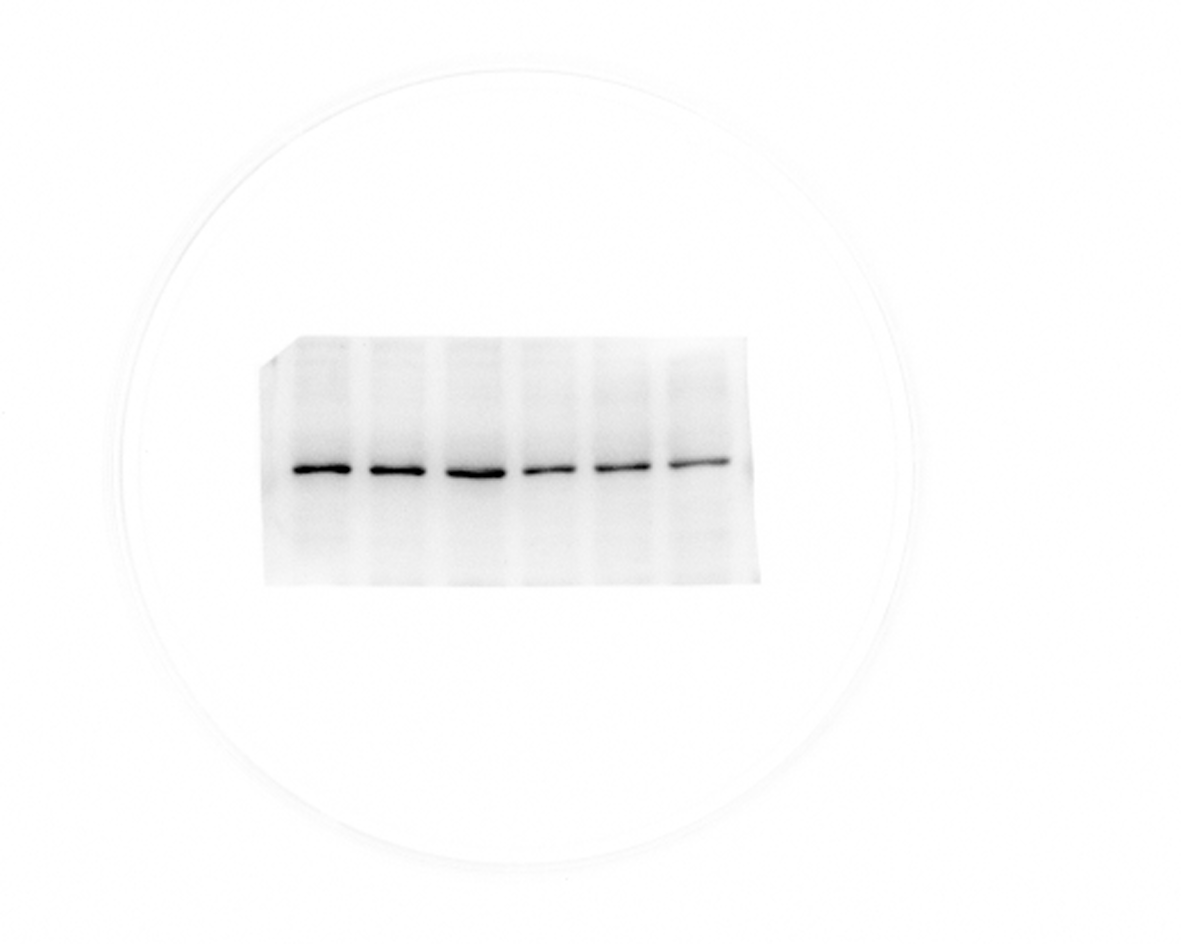

Supplement: Figure 5—source data 1. [file elife-100205-fig5-data1.zip › Figure 5-Source Data 1-Raw uncropped blots/Figure 5H/pCREB-BAT.tif]

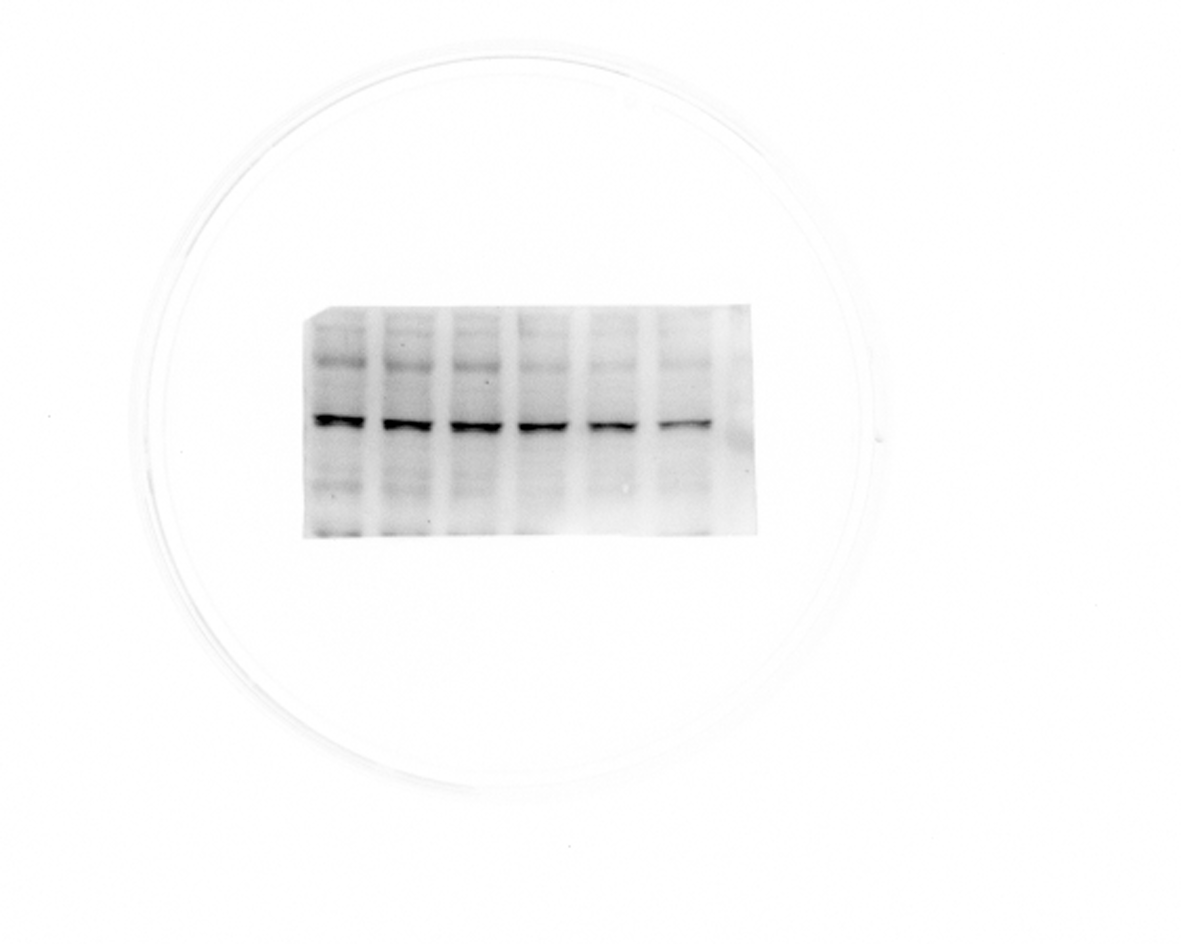

Supplement: Figure 5—source data 1. [file elife-100205-fig5-data1.zip › Figure 5-Source Data 1-Raw uncropped blots/Figure 5H/pCREB-iWAT.tif]

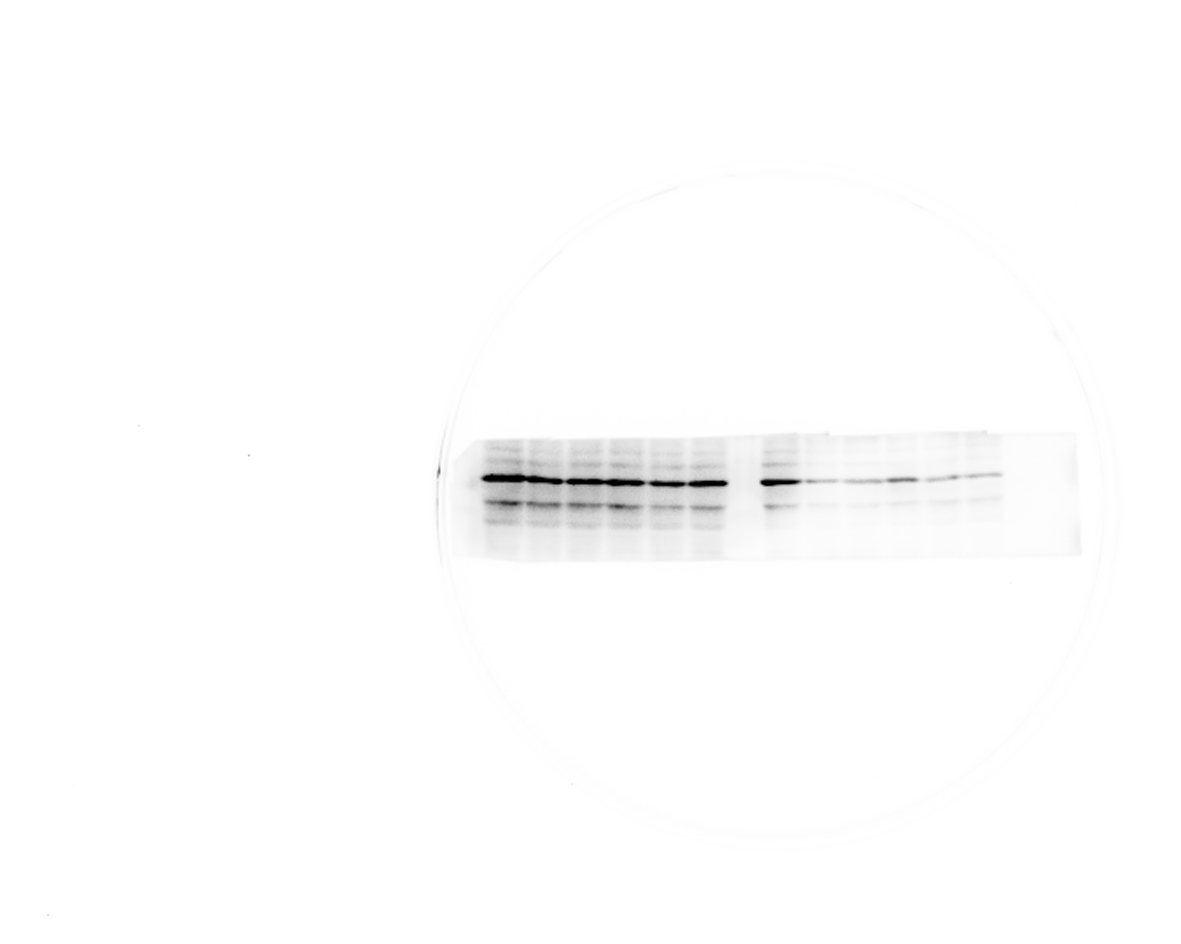

Supplement: Figure 5—source data 1. [file elife-100205-fig5-data1.zip › Figure 5-Source Data 1-Raw uncropped blots/Figure 5I/CREB(Left).tif]

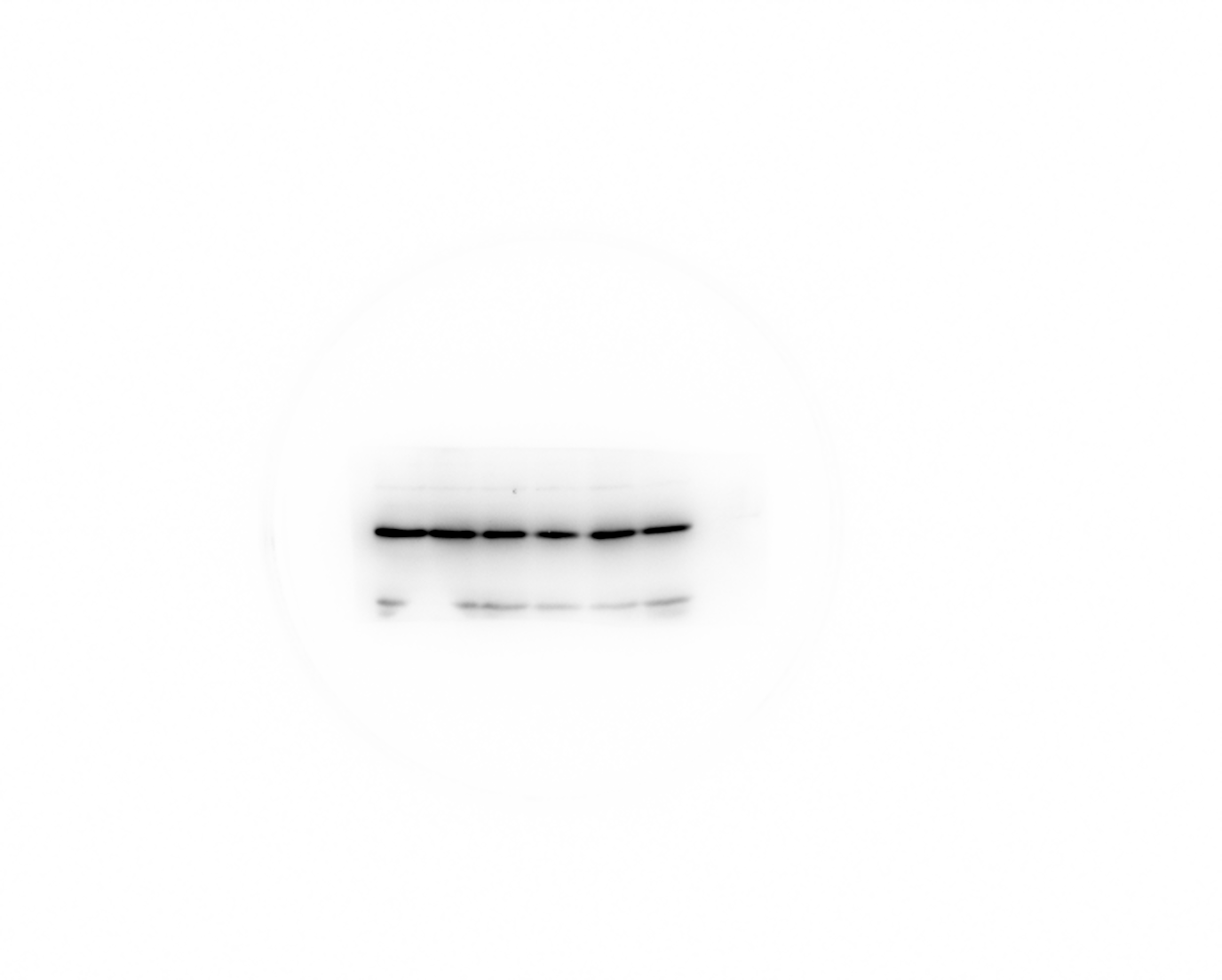

Supplement: Figure 5—source data 1. [file elife-100205-fig5-data1.zip › Figure 5-Source Data 1-Raw uncropped blots/Figure 5I/CREB.tif]

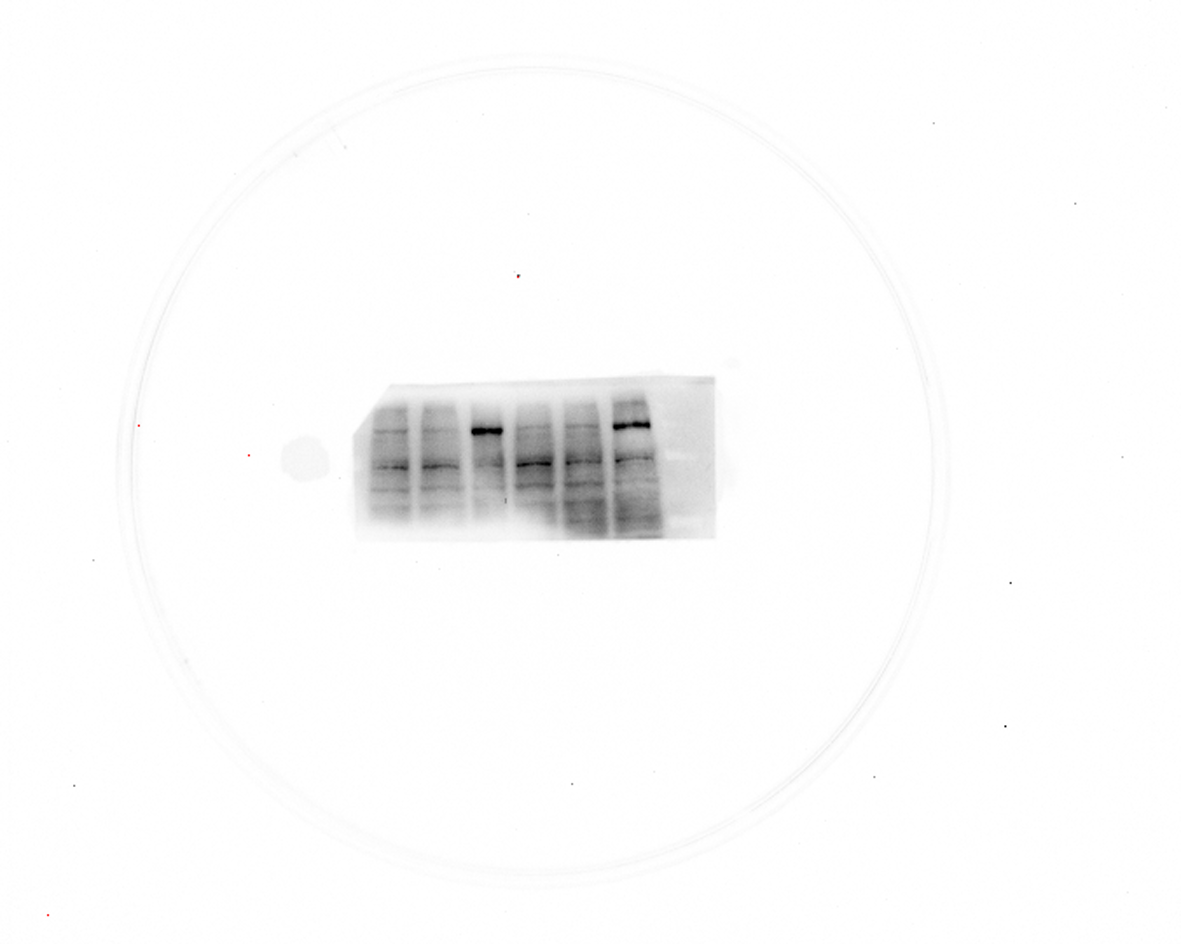

Supplement: Figure 5—source data 1. [file elife-100205-fig5-data1.zip › Figure 5-Source Data 1-Raw uncropped blots/Figure 5I/FLAG.tif]

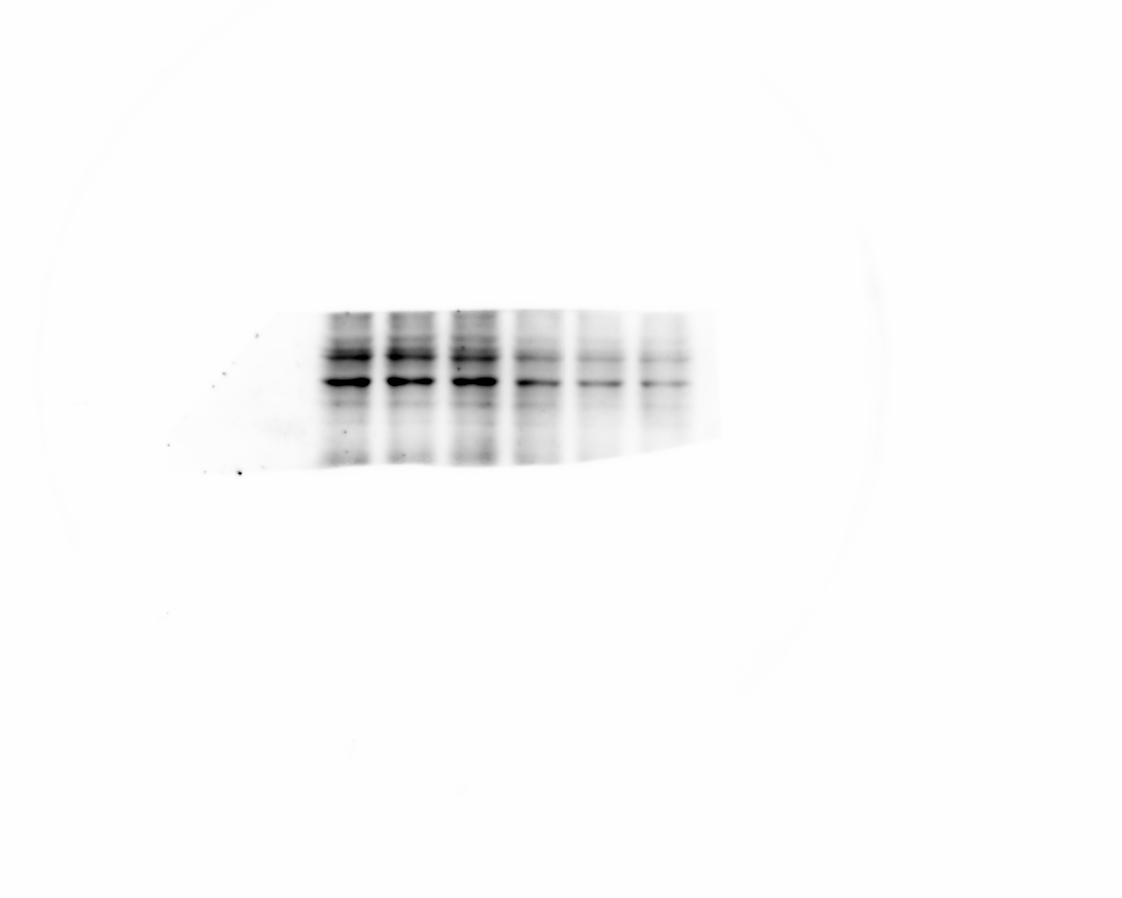

Supplement: Figure 5—source data 1. [file elife-100205-fig5-data1.zip › Figure 5-Source Data 1-Raw uncropped blots/Figure 5I/GNAS.tif]

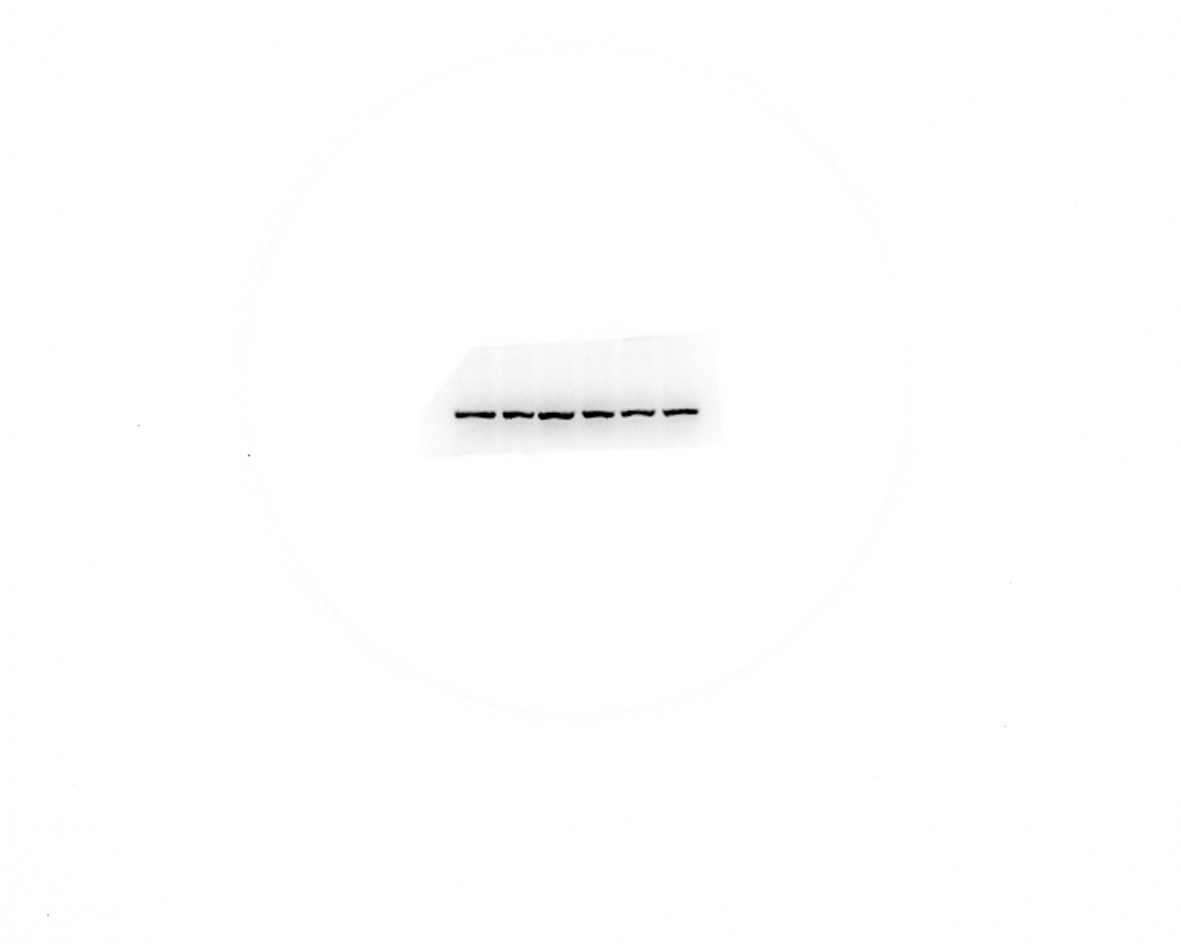

Supplement: Figure 5—source data 1. [file elife-100205-fig5-data1.zip › Figure 5-Source Data 1-Raw uncropped blots/Figure 5I/HSP90.tif]

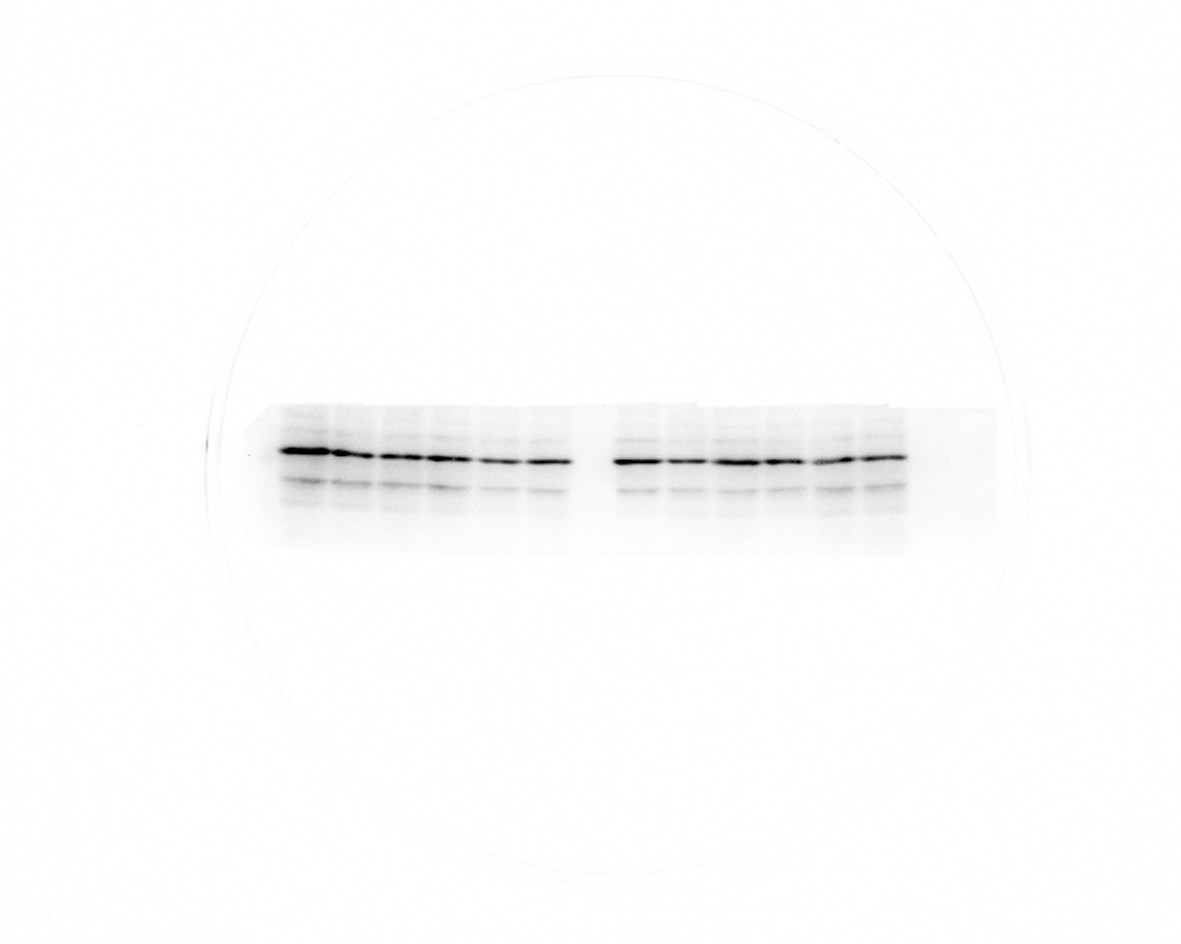

Supplement: Figure 5—source data 1. [file elife-100205-fig5-data1.zip › Figure 5-Source Data 1-Raw uncropped blots/Figure 5I/pCREB(Right).tif]

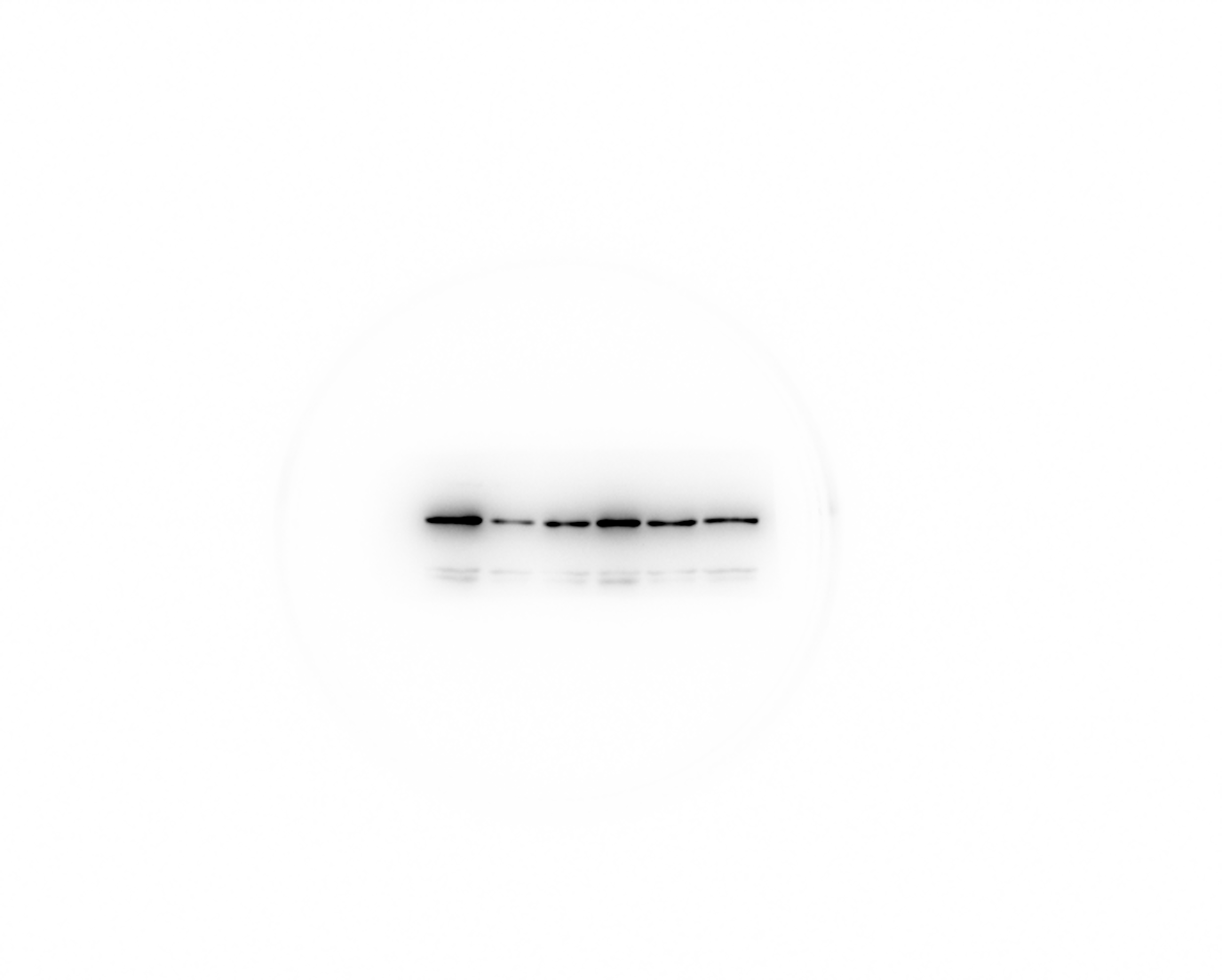

Supplement: Figure 5—source data 1. [file elife-100205-fig5-data1.zip › Figure 5-Source Data 1-Raw uncropped blots/Figure 5I/pCREB.tif]

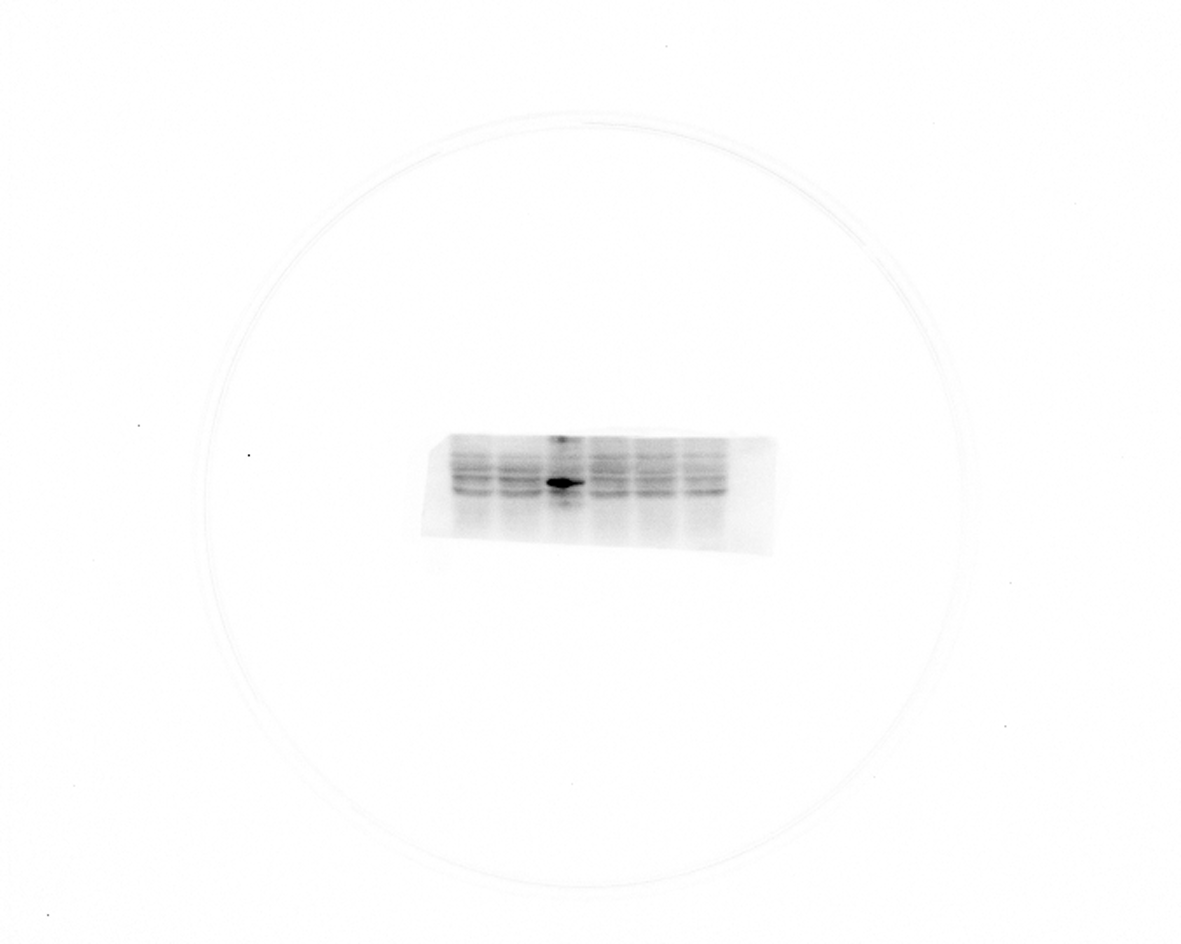

Supplement: Figure 5—source data 1. [file elife-100205-fig5-data1.zip › Figure 5-Source Data 1-Raw uncropped blots/Figure 5I/UCP1.tif]

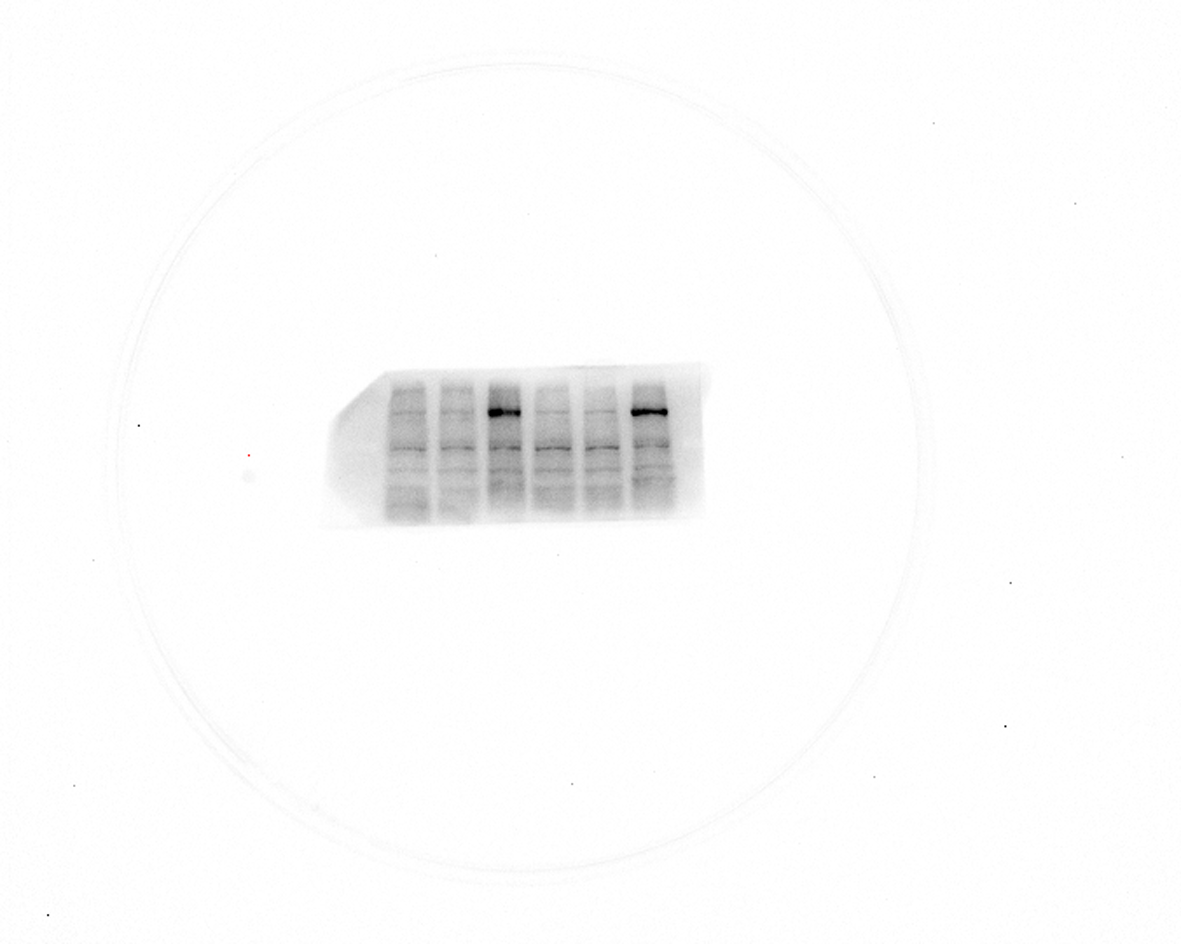

Supplement: Figure 5—source data 1. [file elife-100205-fig5-data1.zip › Figure 5-Source Data 1-Raw uncropped blots/Figure 5J/FLAG.tif]

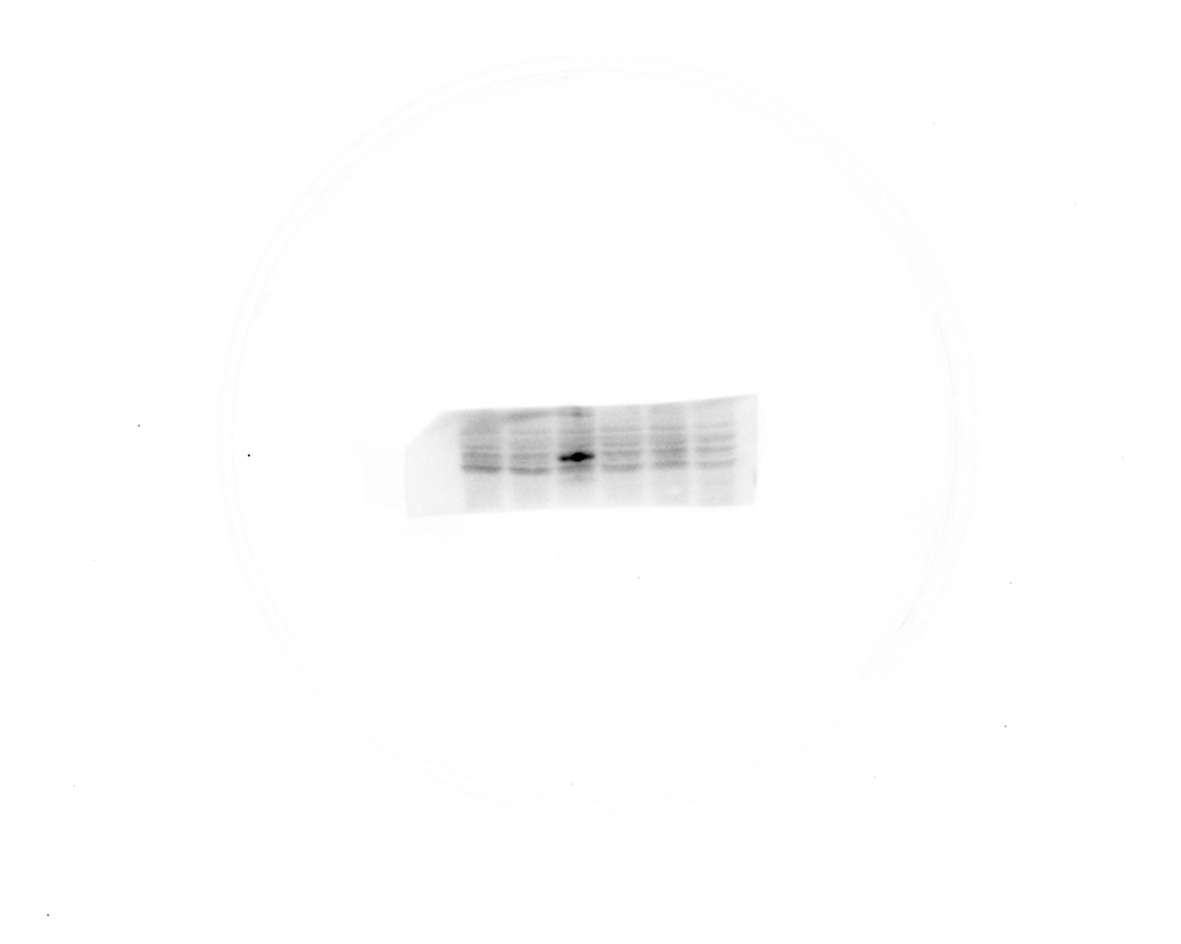

Supplement: Figure 5—source data 1. [file elife-100205-fig5-data1.zip › Figure 5-Source Data 1-Raw uncropped blots/Figure 5J/UCP1.tif]

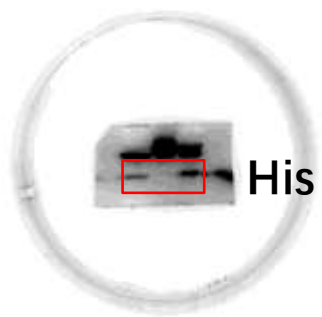

His

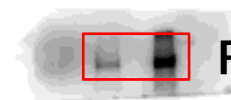

FLAG

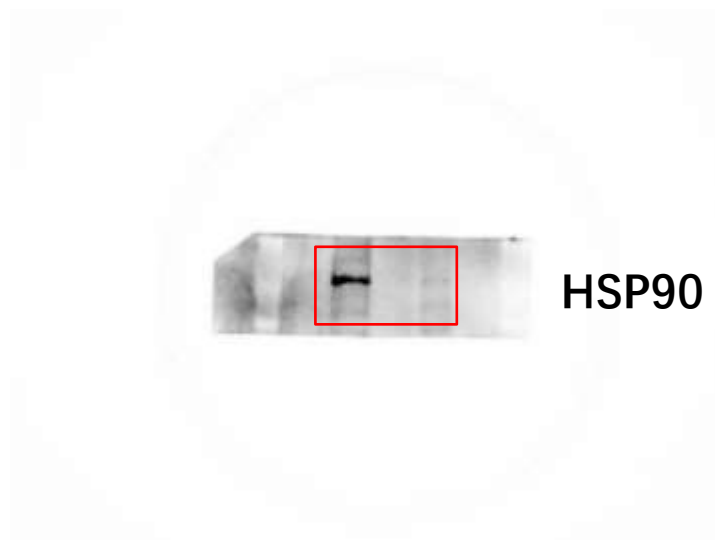

HSP90

Supplement: Figure 5—source data 2. [file elife-100205-fig5-data2.zip › Figure 5-Source Data 2 -Uncropped and labeled blots/Figure 5A.pdf]

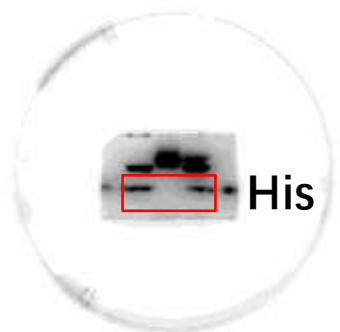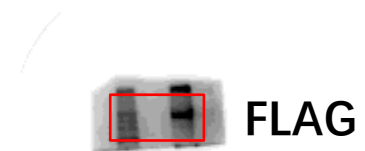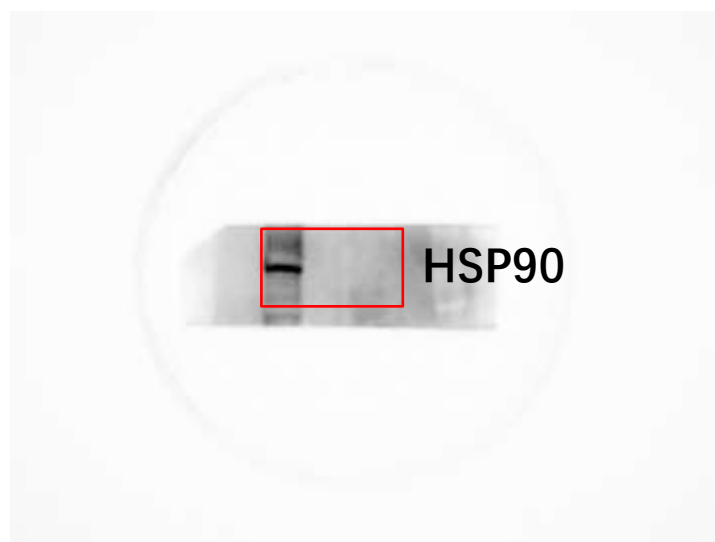

Supplement: Figure 5—source data 2. [file elife-100205-fig5-data2.zip › Figure 5-Source Data 2 -Uncropped and labeled blots/Figure 5B.pdf]

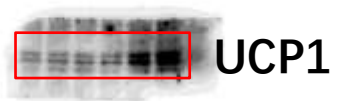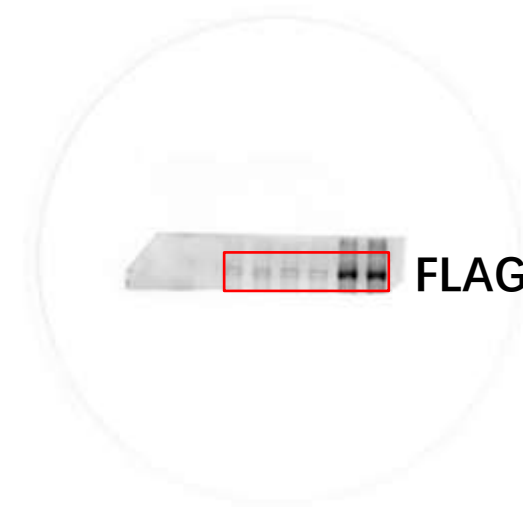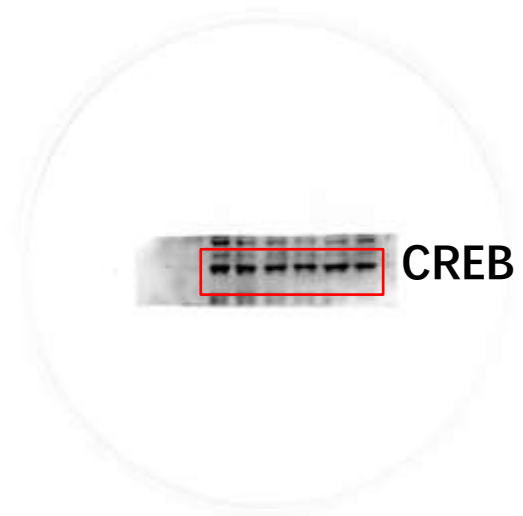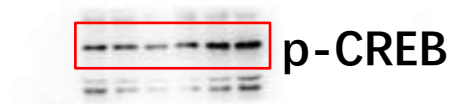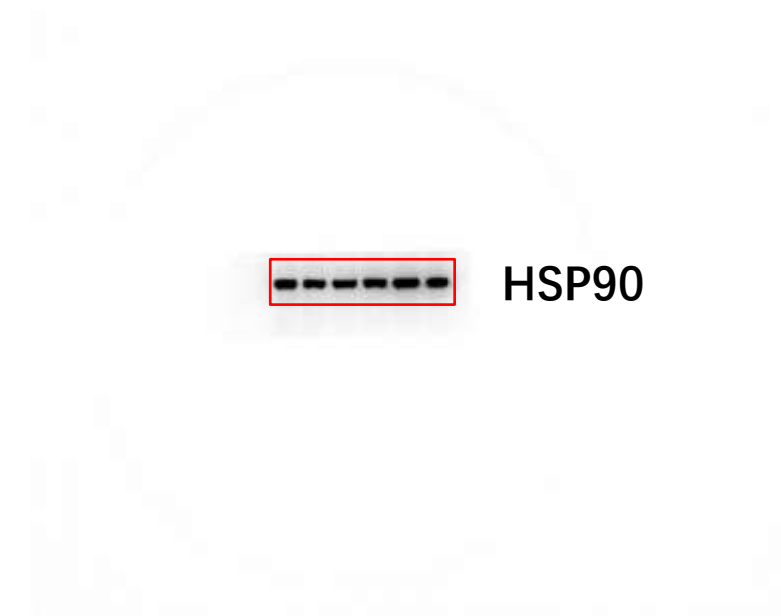

Supplement: Figure 5—source data 2. [file elife-100205-fig5-data2.zip › Figure 5-Source Data 2 -Uncropped and labeled blots/Figure 5E.pdf]

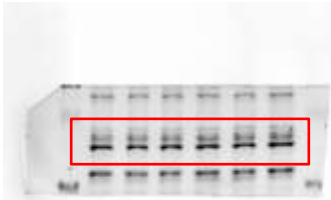

**CREB (iWAT)**

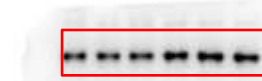

**p-CREB (iWAT)**

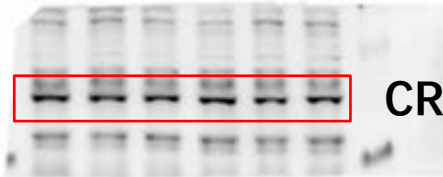

**CREB (BAT)**

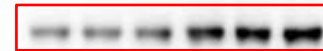

**p-CREB (BAT)**

Supplement: Figure 5—source data 2. [file elife-100205-fig5-data2.zip › Figure 5-Source Data 2 -Uncropped and labeled blots/Figure 5F.pdf]

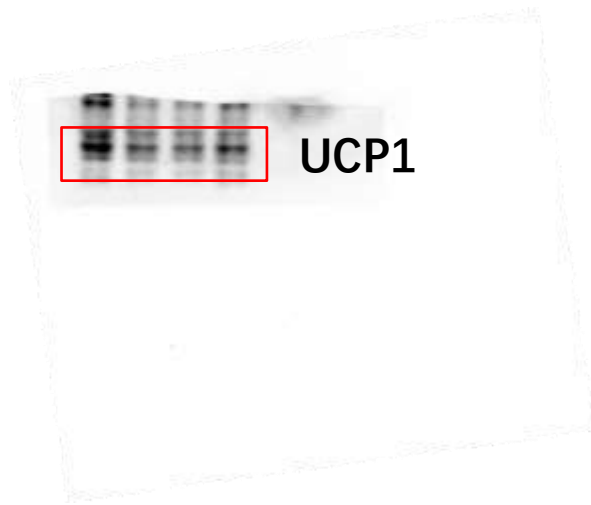

**UCP1**

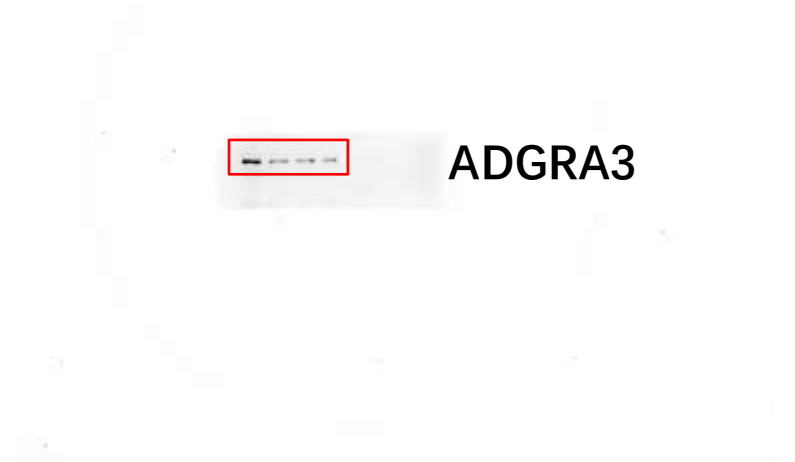

**ADGRA3**

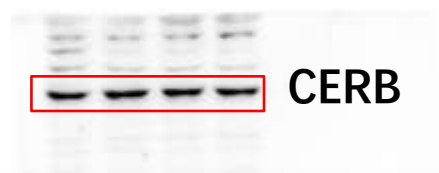

**CERB**

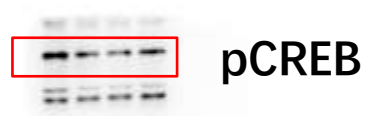

**pCREB**

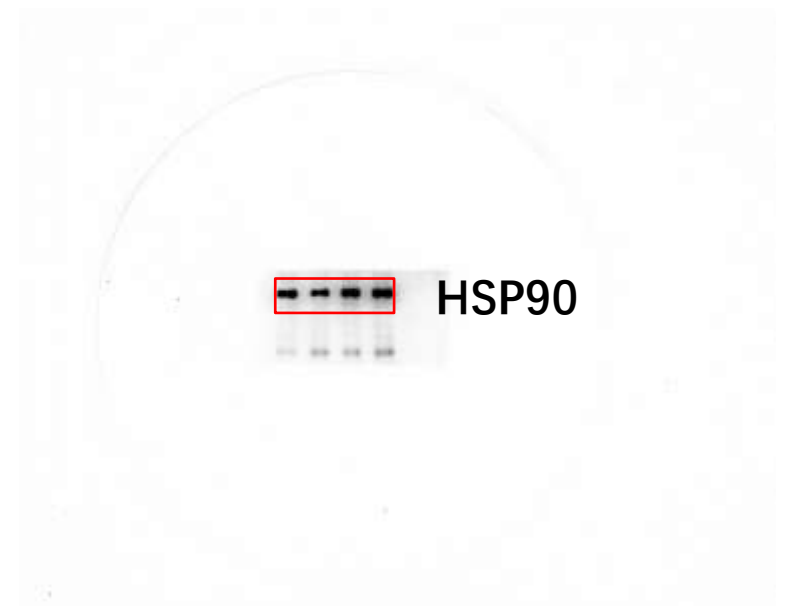

**HSP90**

Supplement: Figure 5—source data 2. [file elife-100205-fig5-data2.zip › Figure 5-Source Data 2 -Uncropped and labeled blots/Figure 5G.pdf]

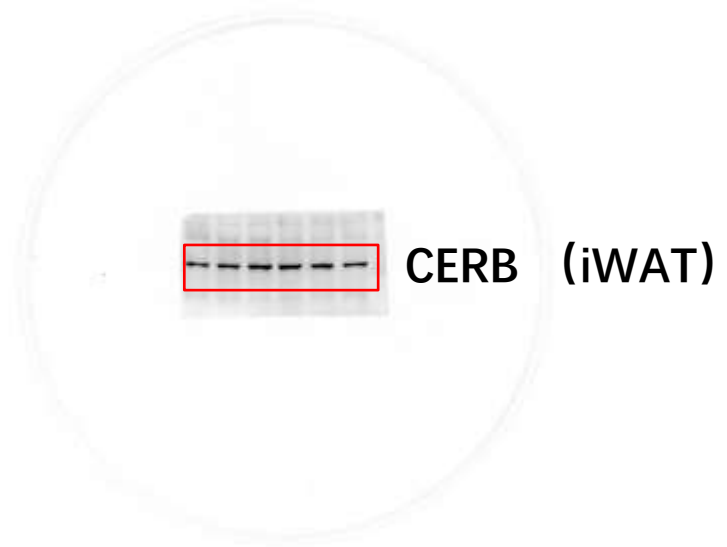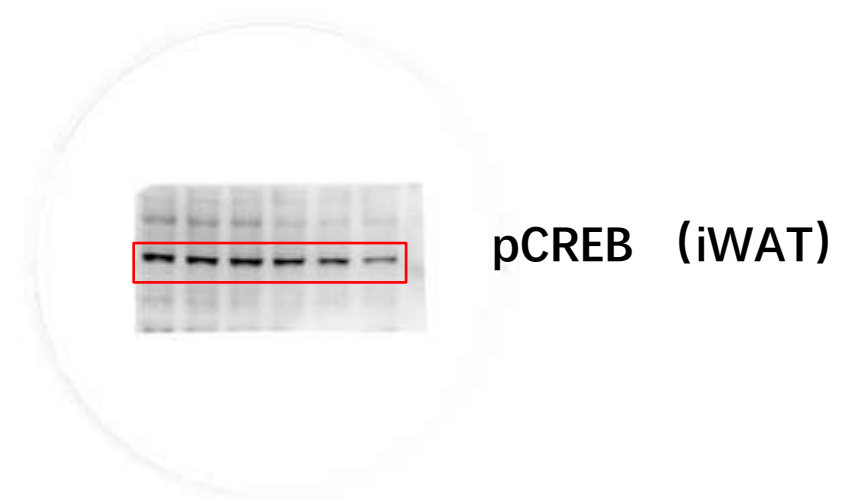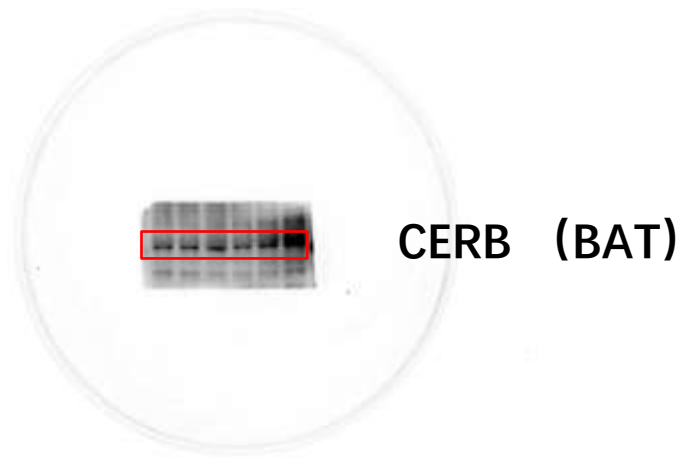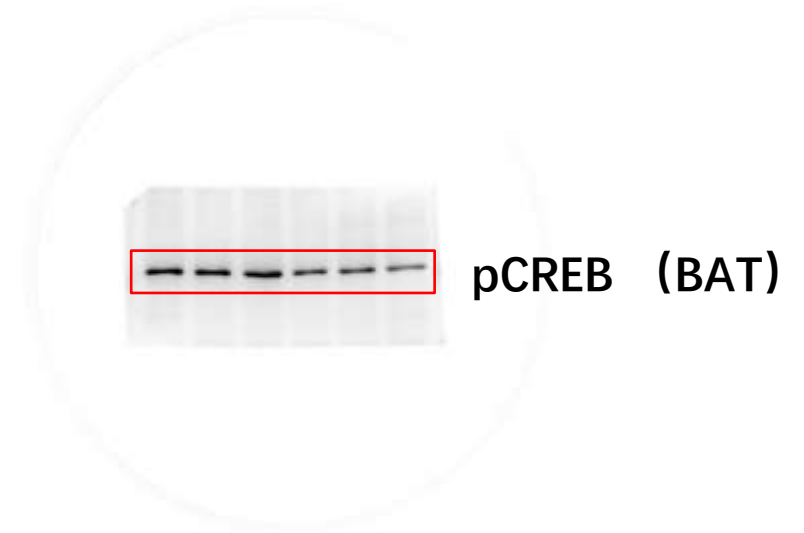

Supplement: Figure 5—source data 2. [file elife-100205-fig5-data2.zip › Figure 5-Source Data 2 -Uncropped and labeled blots/Figure 5H.pdf]

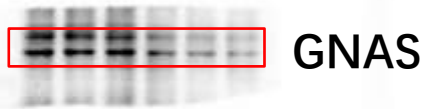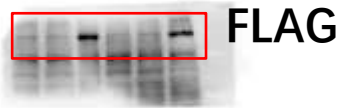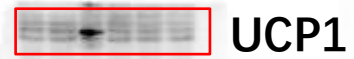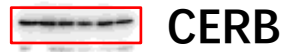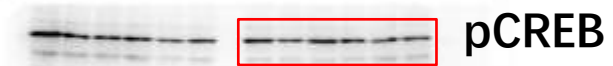

Supplement: Figure 5—source data 2. [file elife-100205-fig5-data2.zip › Figure 5-Source Data 2 -Uncropped and labeled blots/Figure 5I.pdf]

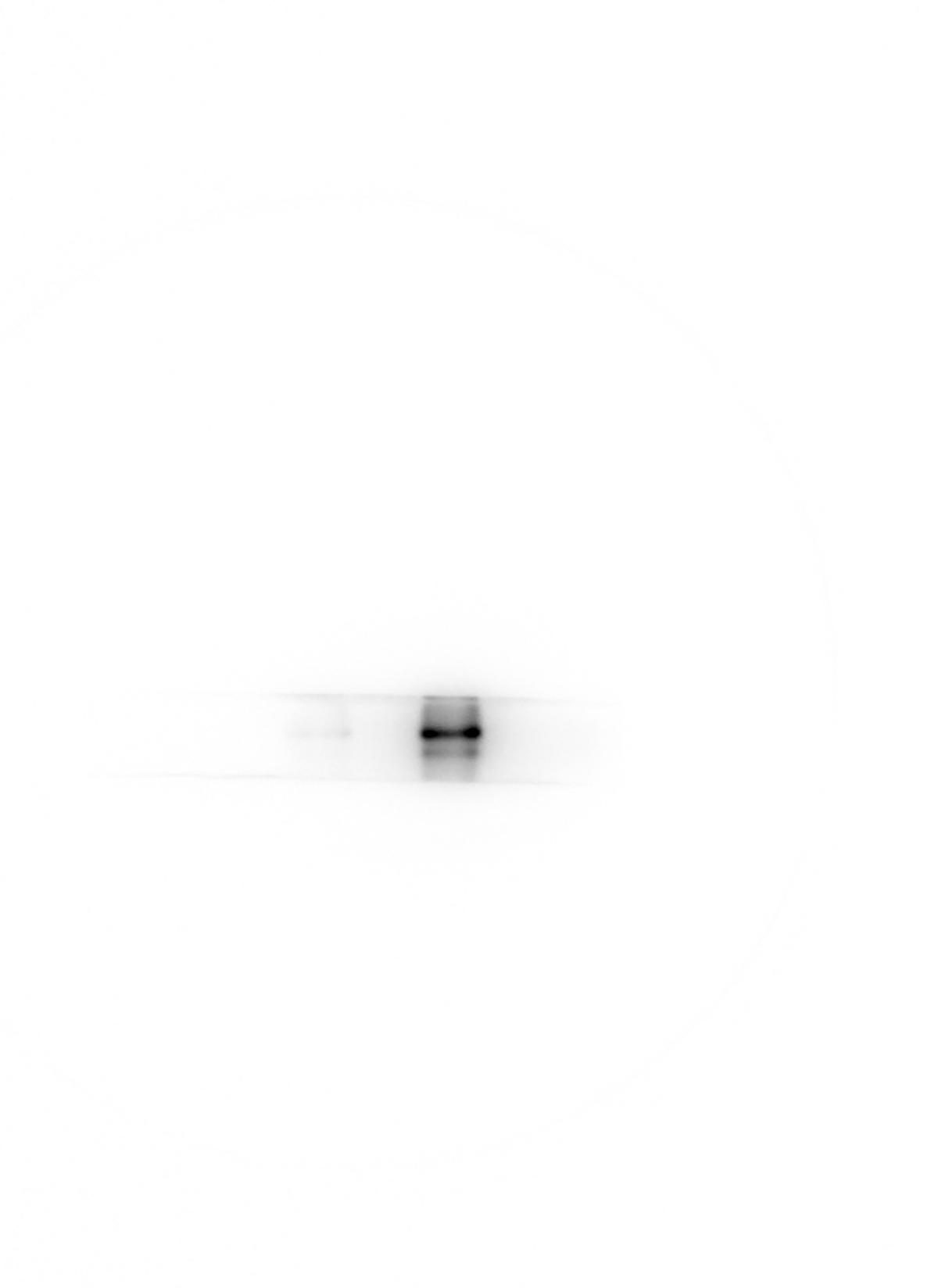

Supplement: Figure 5—figure supplement 1—source data 1. [file elife-100205-fig5-figsupp1-data1.zip › Figure 5-figure supplement 1-Source Data 1-Raw uncropped blots/Figure 5-figure supplement 1A/FLAG.jpg]

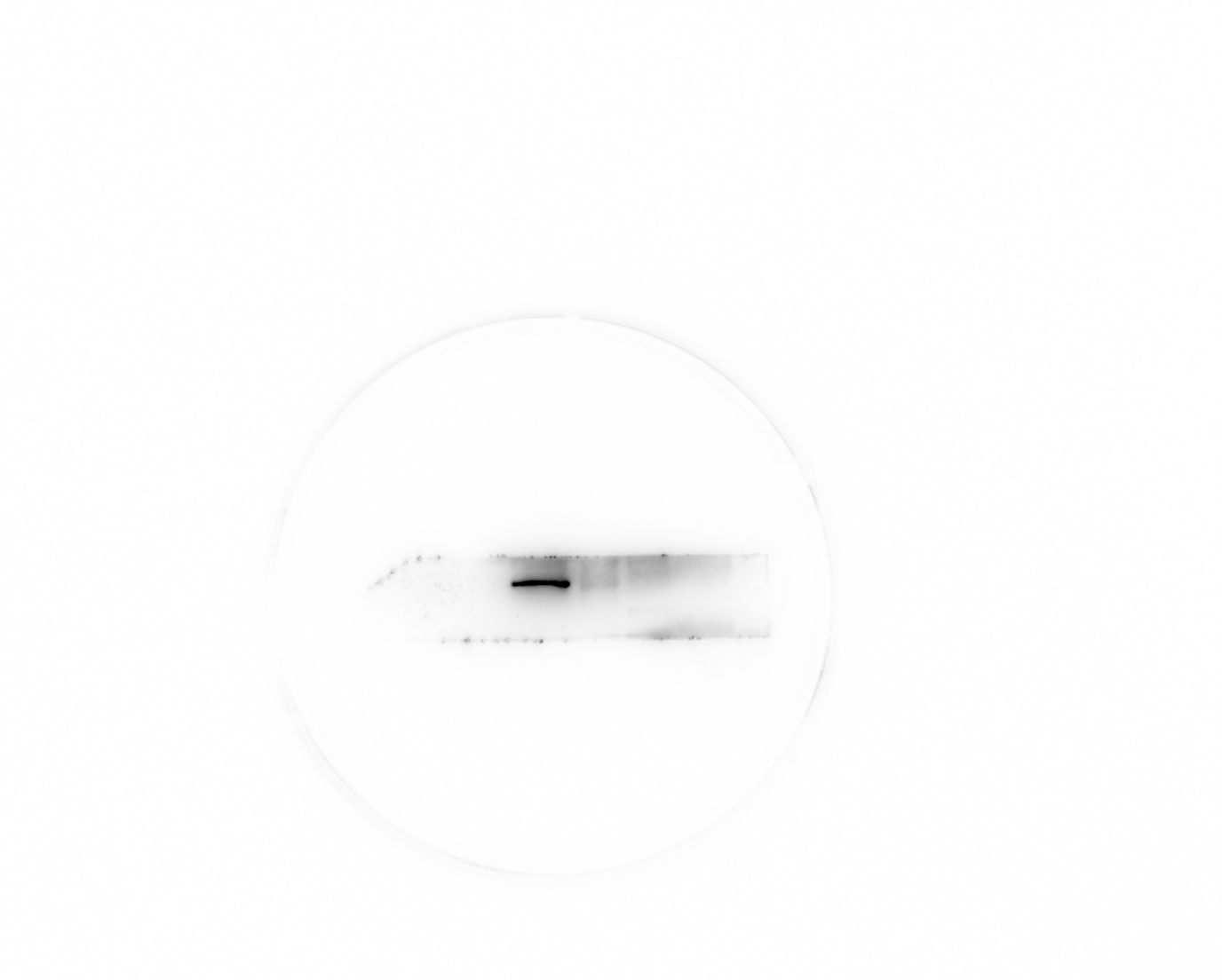

Supplement: Figure 5—figure supplement 1—source data 1. [file elife-100205-fig5-figsupp1-data1.zip › Figure 5-figure supplement 1-Source Data 1-Raw uncropped blots/Figure 5-figure supplement 1A/HIS.tif]

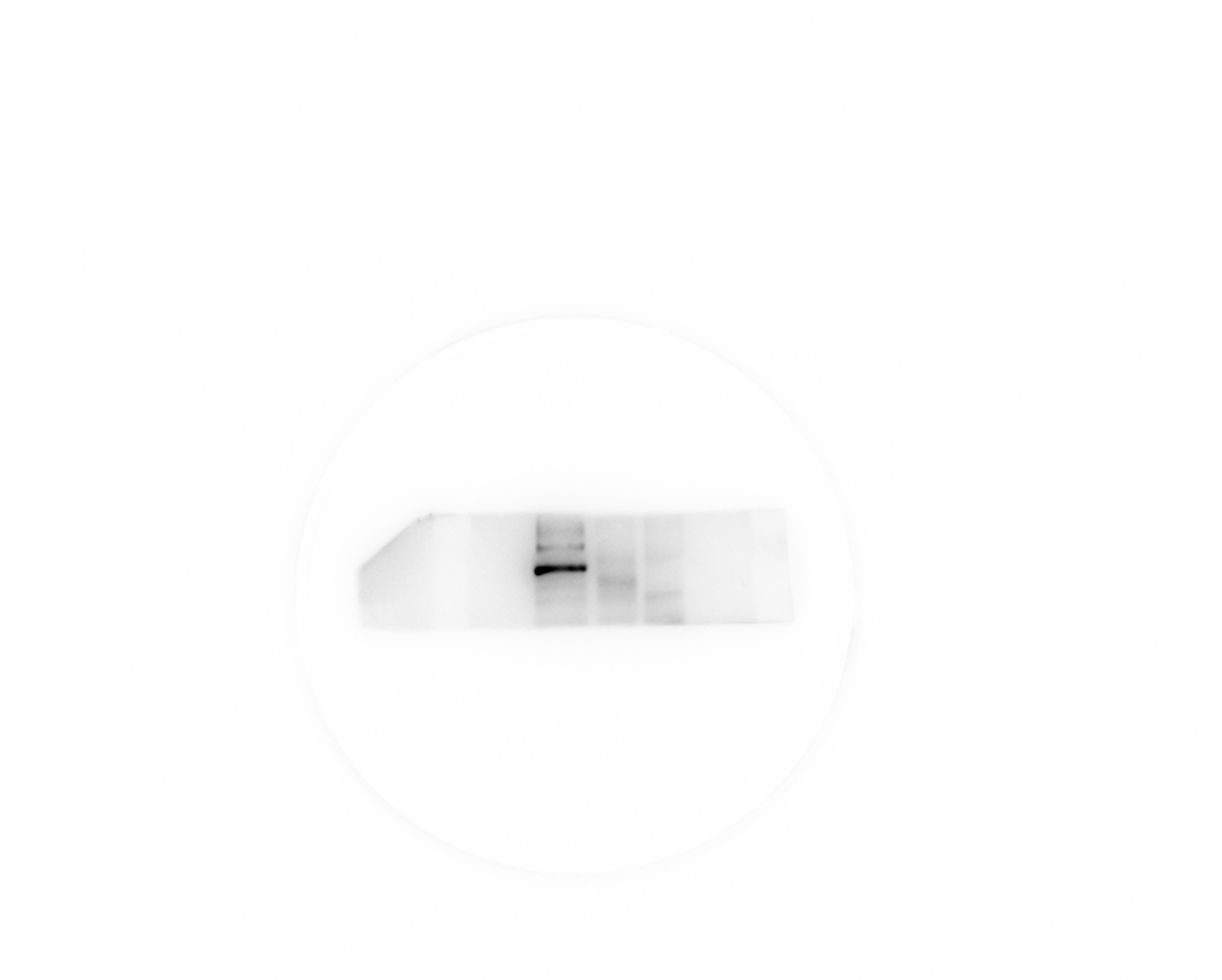

Supplement: Figure 5—figure supplement 1—source data 1. [file elife-100205-fig5-figsupp1-data1.zip › Figure 5-figure supplement 1-Source Data 1-Raw uncropped blots/Figure 5-figure supplement 1A/HSP90.tif]

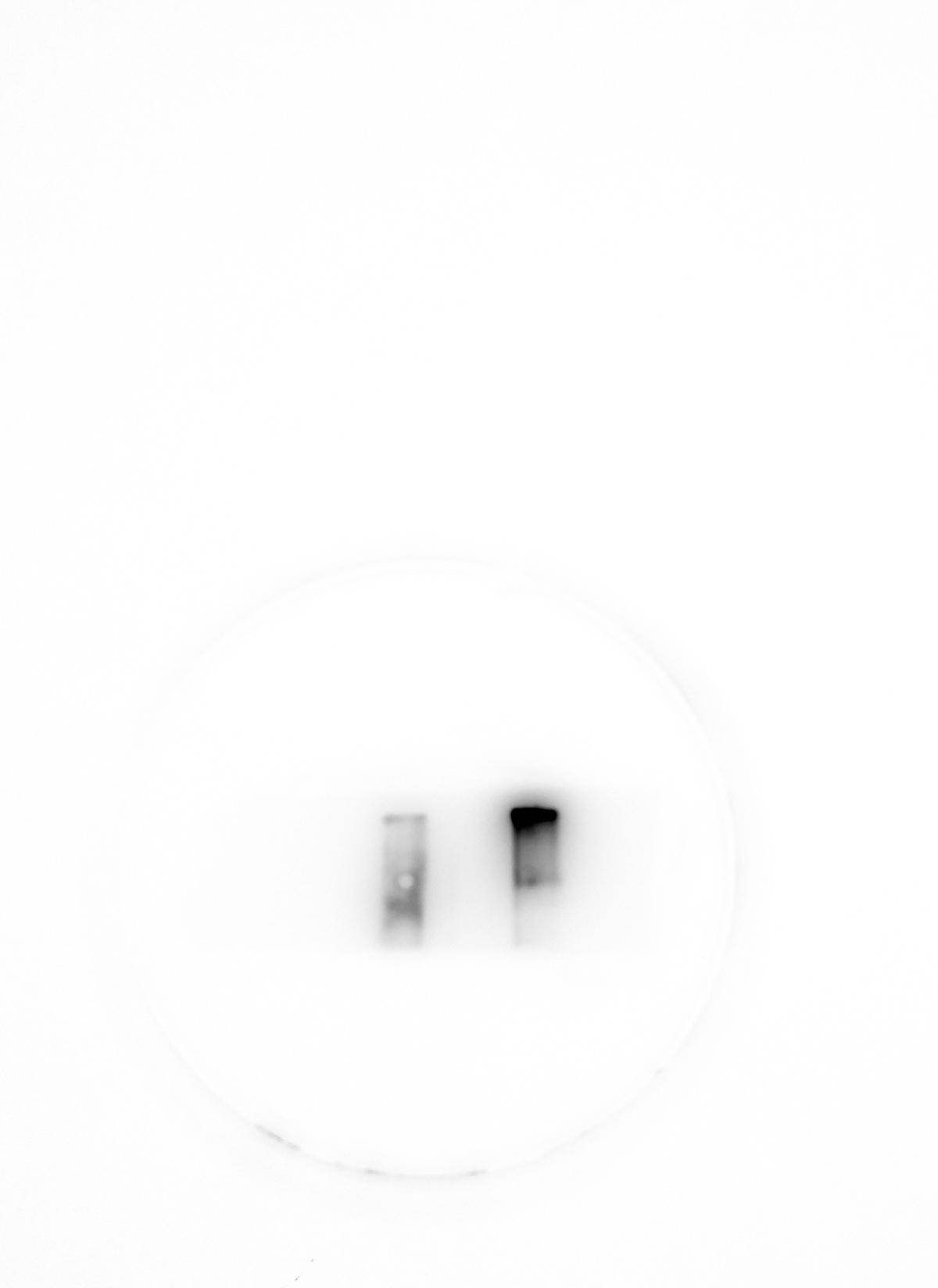

Supplement: Figure 5—figure supplement 1—source data 1. [file elife-100205-fig5-figsupp1-data1.zip › Figure 5-figure supplement 1-Source Data 1-Raw uncropped blots/Figure 5-figure supplement 1B/FLAG.jpg]

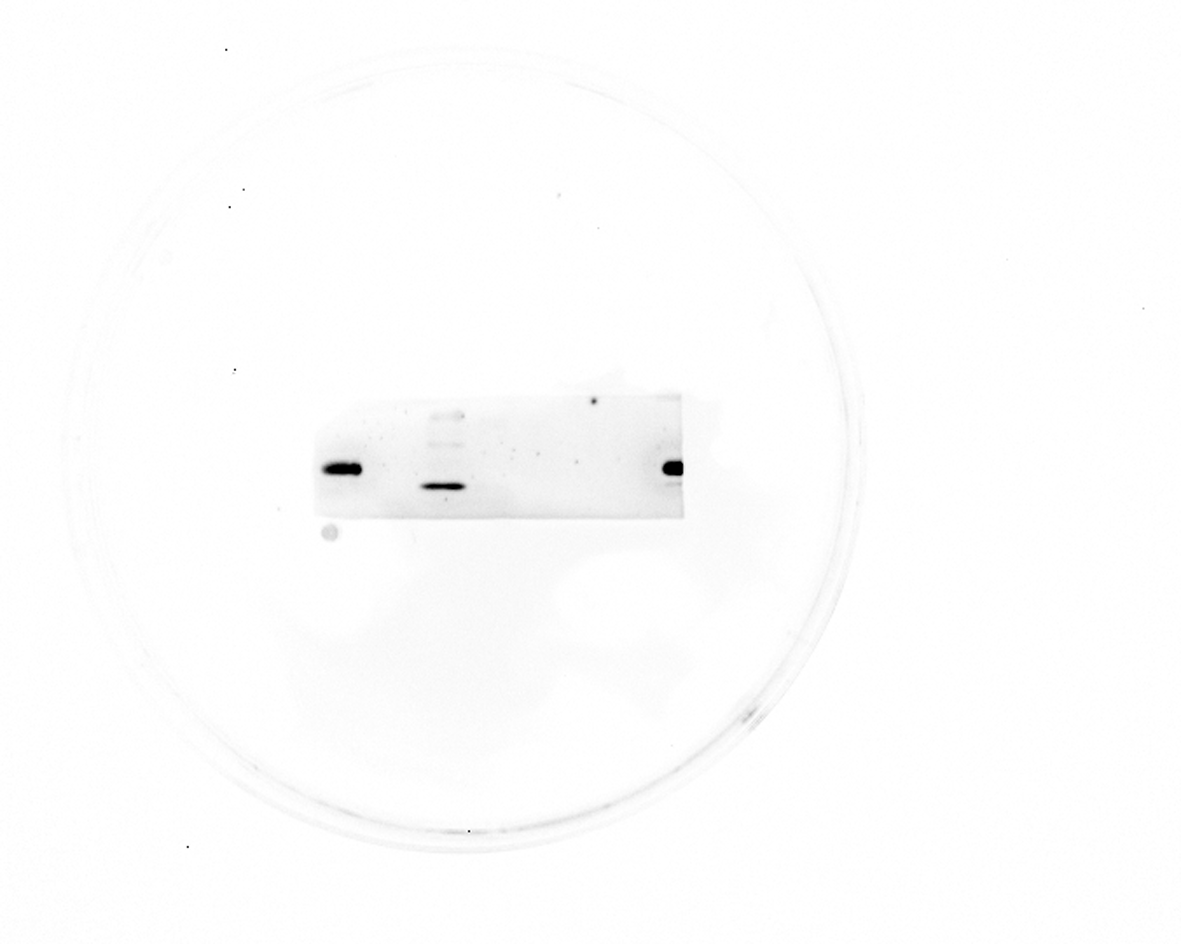

Supplement: Figure 5—figure supplement 1—source data 1. [file elife-100205-fig5-figsupp1-data1.zip › Figure 5-figure supplement 1-Source Data 1-Raw uncropped blots/Figure 5-figure supplement 1B/HIS.tif]

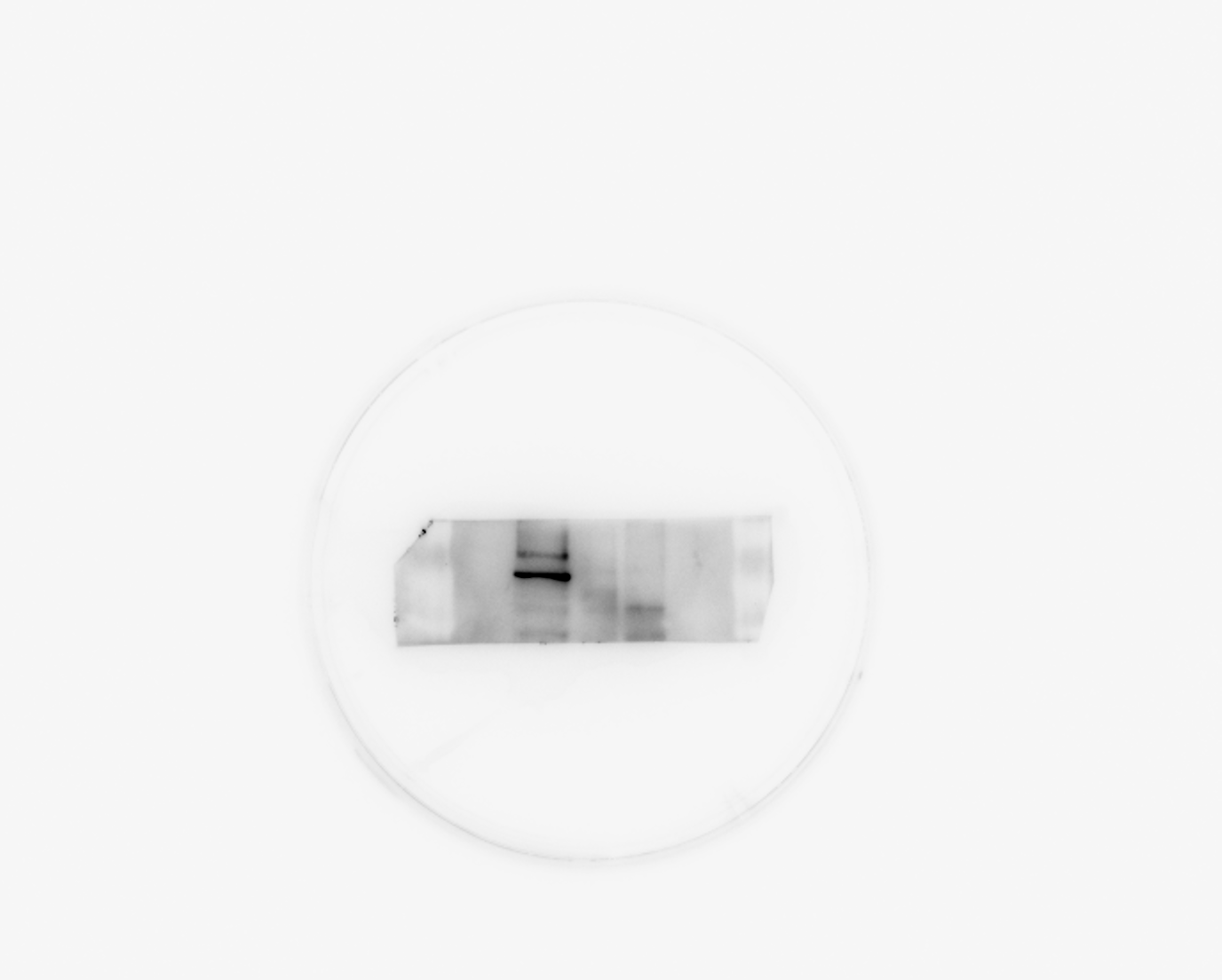

Supplement: Figure 5—figure supplement 1—source data 1. [file elife-100205-fig5-figsupp1-data1.zip › Figure 5-figure supplement 1-Source Data 1-Raw uncropped blots/Figure 5-figure supplement 1B/HSP90.tif]

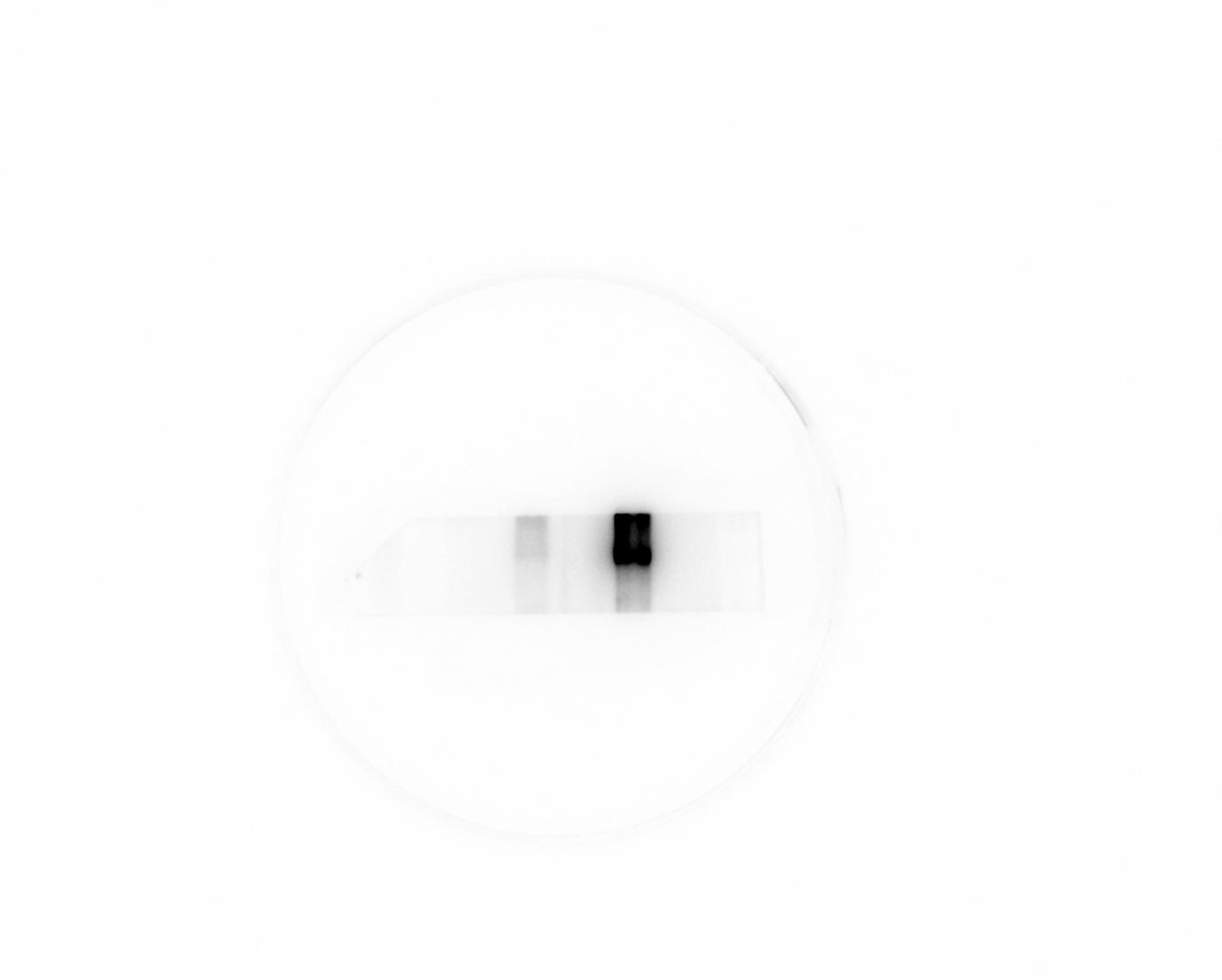

Supplement: Figure 5—figure supplement 1—source data 1. [file elife-100205-fig5-figsupp1-data1.zip › Figure 5-figure supplement 1-Source Data 1-Raw uncropped blots/Figure 5-figure supplement 1C/FLAG.tif]

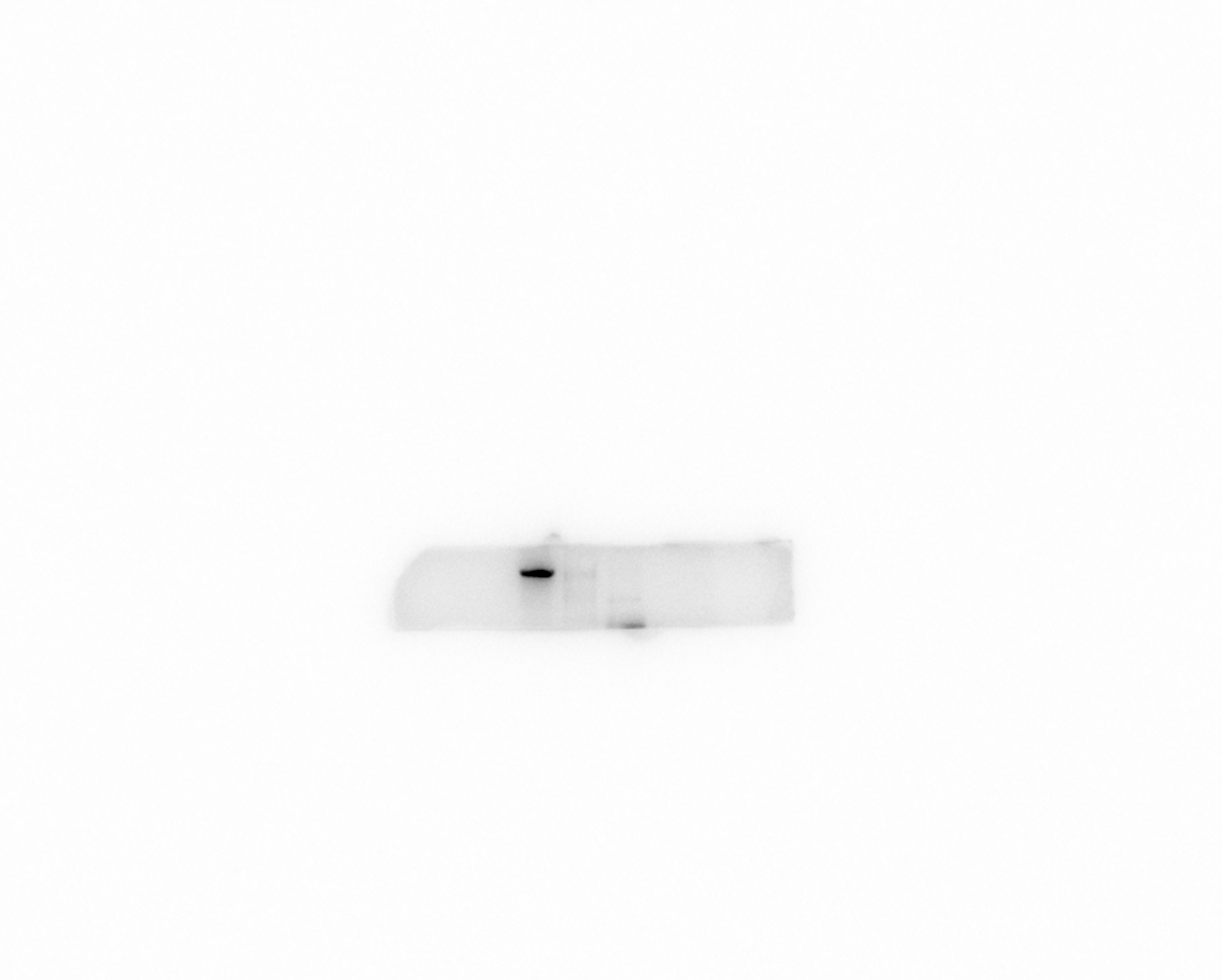

Supplement: Figure 5—figure supplement 1—source data 1. [file elife-100205-fig5-figsupp1-data1.zip › Figure 5-figure supplement 1-Source Data 1-Raw uncropped blots/Figure 5-figure supplement 1C/HIS.tif]

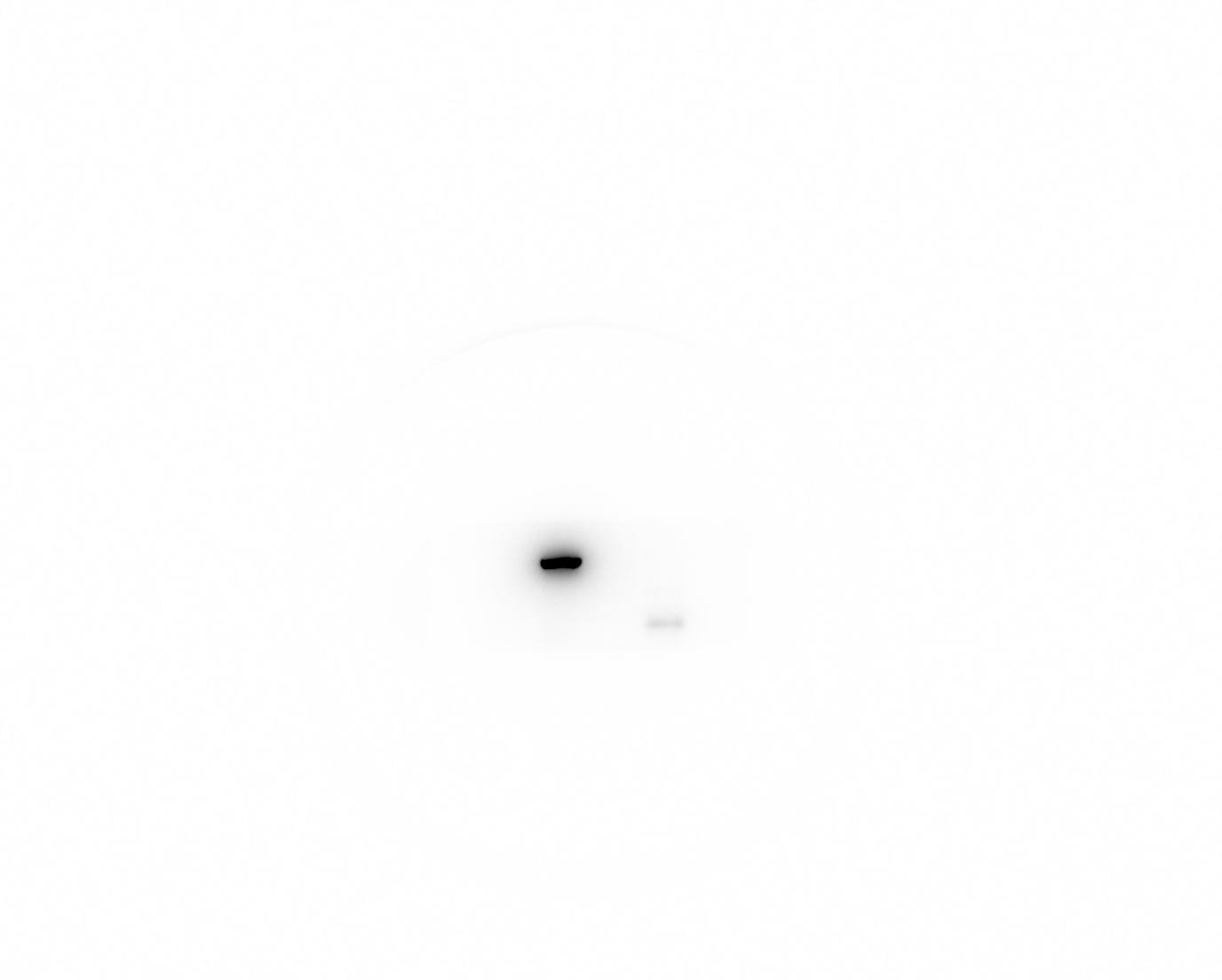

Supplement: Figure 5—figure supplement 1—source data 1. [file elife-100205-fig5-figsupp1-data1.zip › Figure 5-figure supplement 1-Source Data 1-Raw uncropped blots/Figure 5-figure supplement 1C/HSP90.tif]

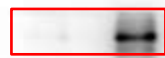

FLAG

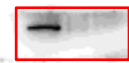

HIS

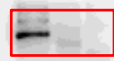

HSP90

Supplement: Figure 5—figure supplement 1—source data 2. [file elife-100205-fig5-figsupp1-data2.zip › Figure 5-figure supplement 1-Source Data 2 -Uncropped and labeled blots/Figure 5-figure supplement 1A.pdf]

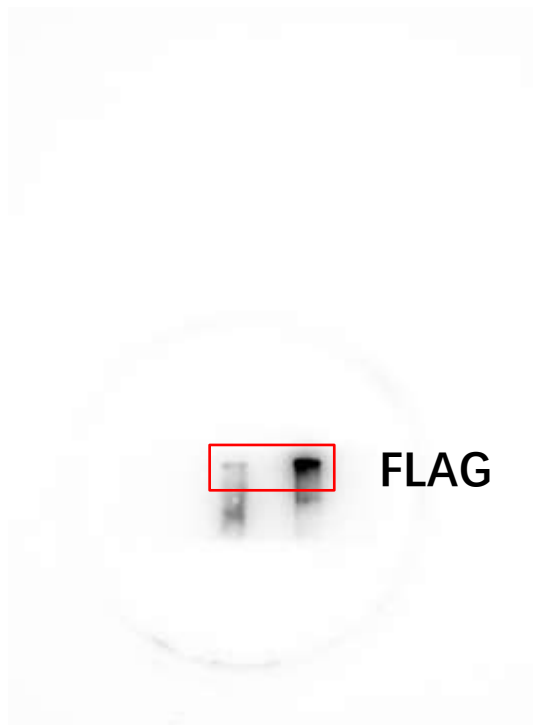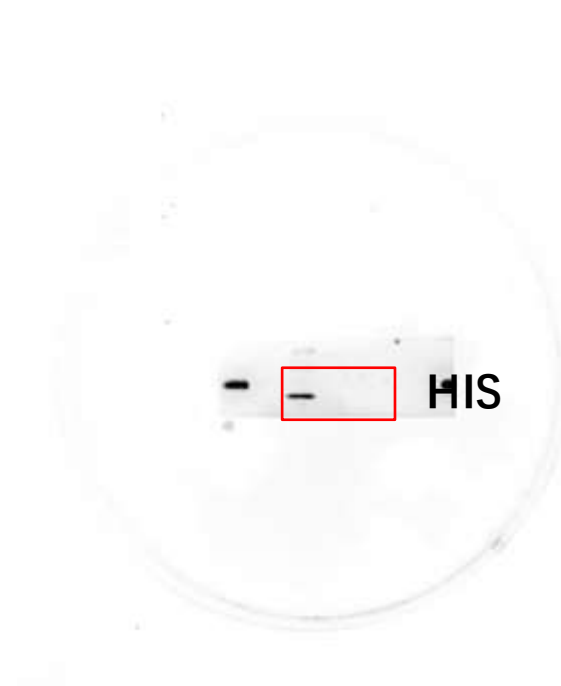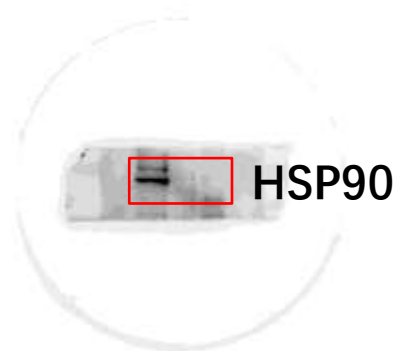

Supplement: Figure 5—figure supplement 1—source data 2. [file elife-100205-fig5-figsupp1-data2.zip › Figure 5-figure supplement 1-Source Data 2 -Uncropped and labeled blots/Figure 5-figure supplement 1B.pdf]

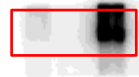

FLAG

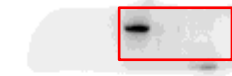

HIS

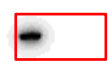

HSP90

Supplement: Figure 5—figure supplement 1—source data 2. [file elife-100205-fig5-figsupp1-data2.zip › Figure 5-figure supplement 1-Source Data 2 -Uncropped and labeled blots/Figure 5-figure supplement 1C.pdf]

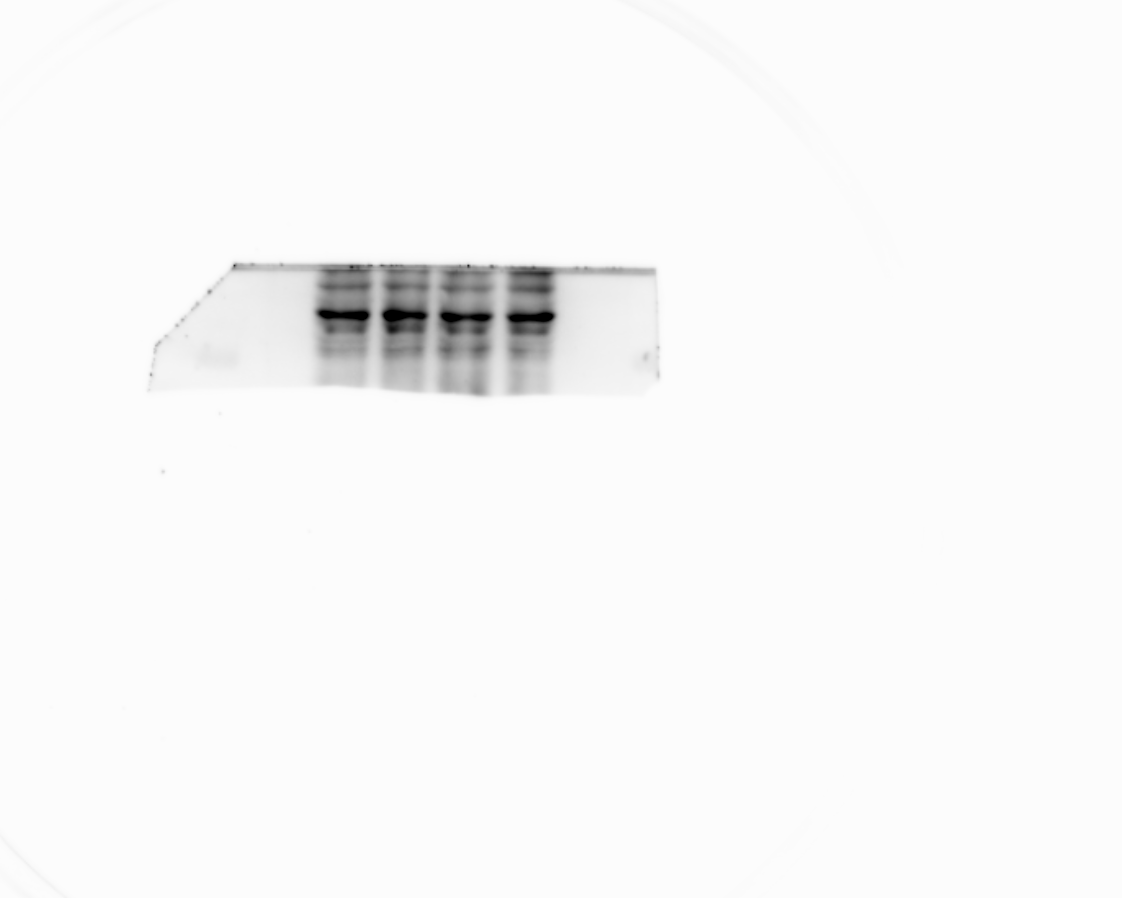

Supplement: Figure 6—source data 1. [file elife-100205-fig6-data1.zip › Figure 6-Source Data 1-Raw uncropped blots/Figure 6D/CREB.tif]

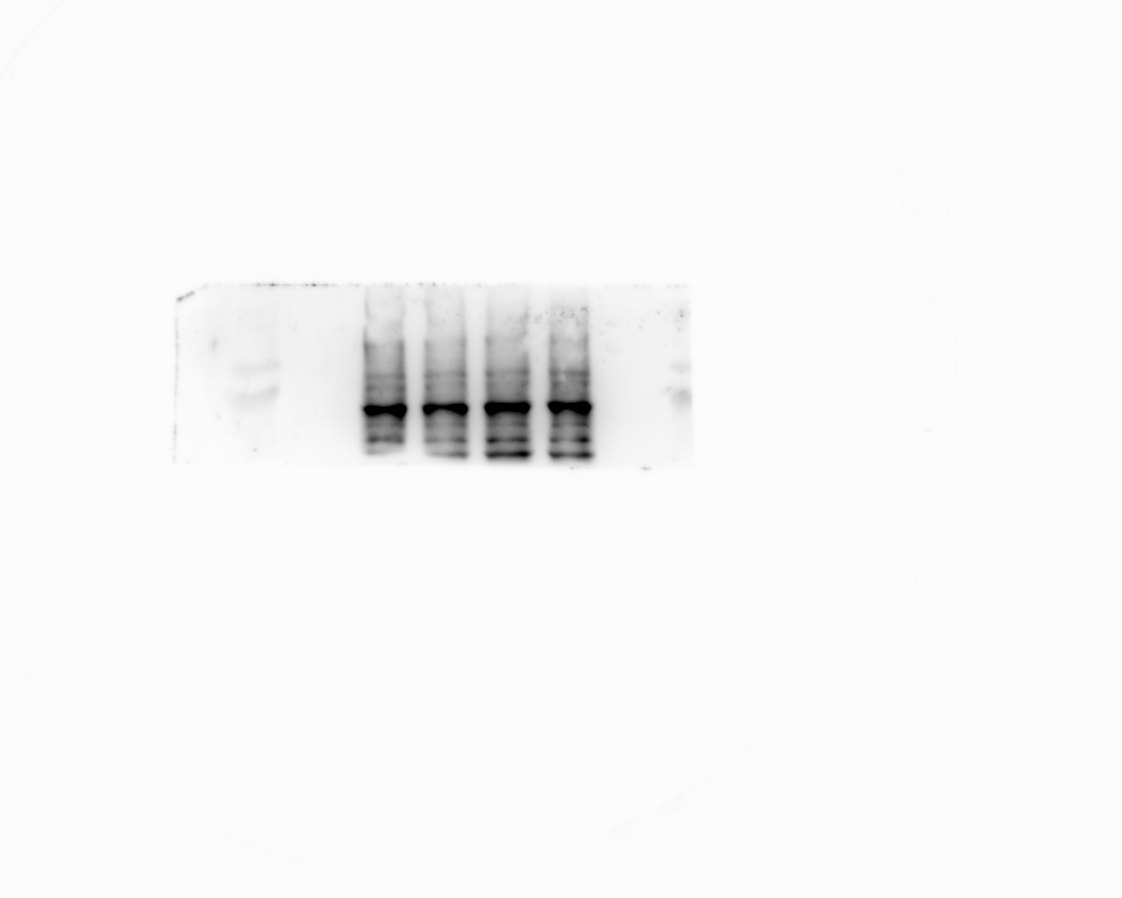

Supplement: Figure 6—source data 1. [file elife-100205-fig6-data1.zip › Figure 6-Source Data 1-Raw uncropped blots/Figure 6D/HSP90.tif]

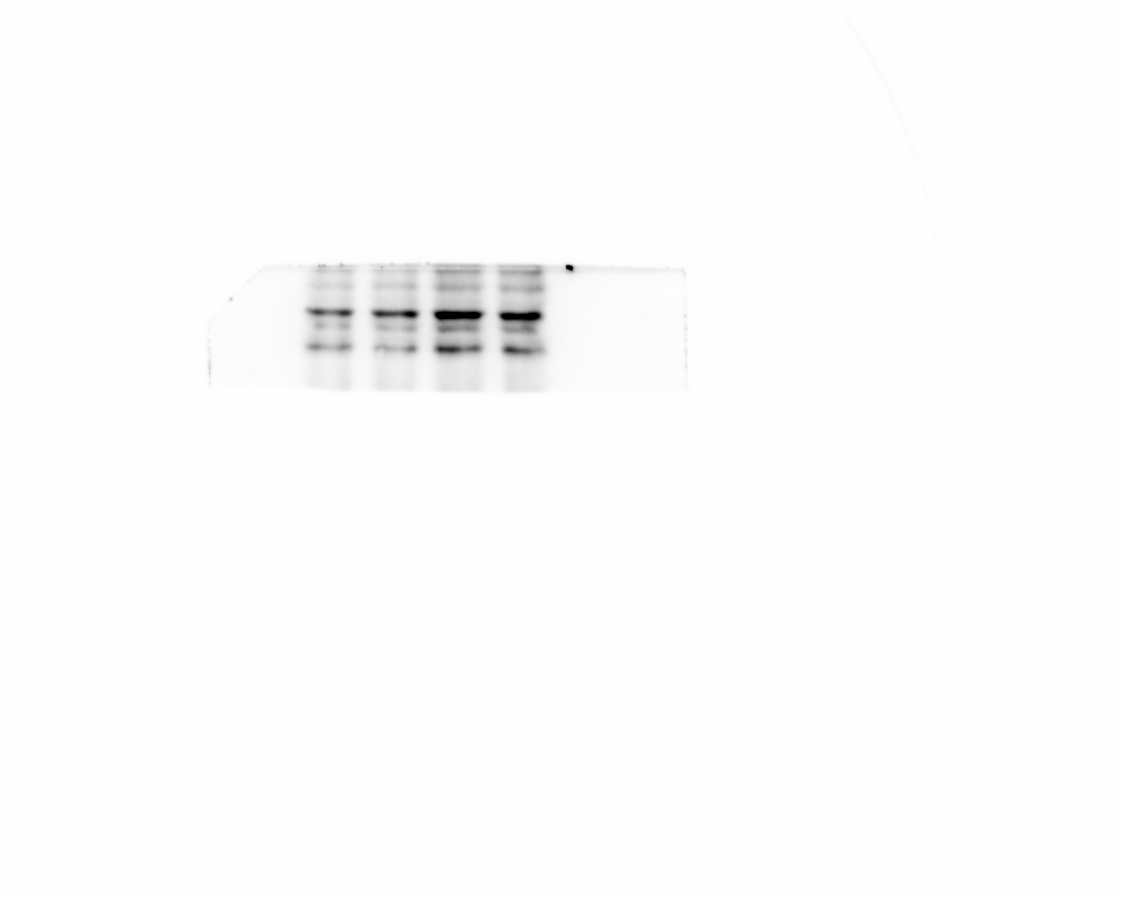

Supplement: Figure 6—source data 1. [file elife-100205-fig6-data1.zip › Figure 6-Source Data 1-Raw uncropped blots/Figure 6D/pCREB.tif]

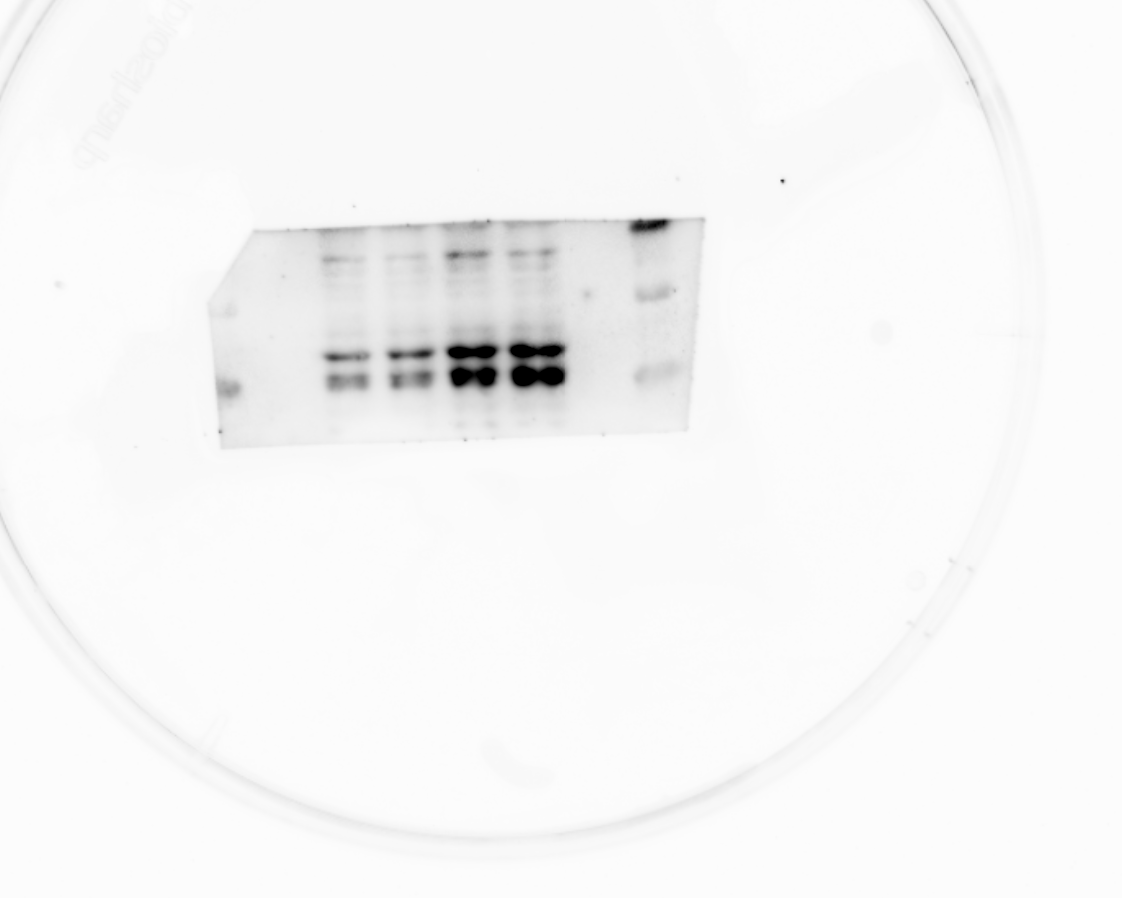

Supplement: Figure 6—source data 1. [file elife-100205-fig6-data1.zip › Figure 6-Source Data 1-Raw uncropped blots/Figure 6D/UCP1.tif]

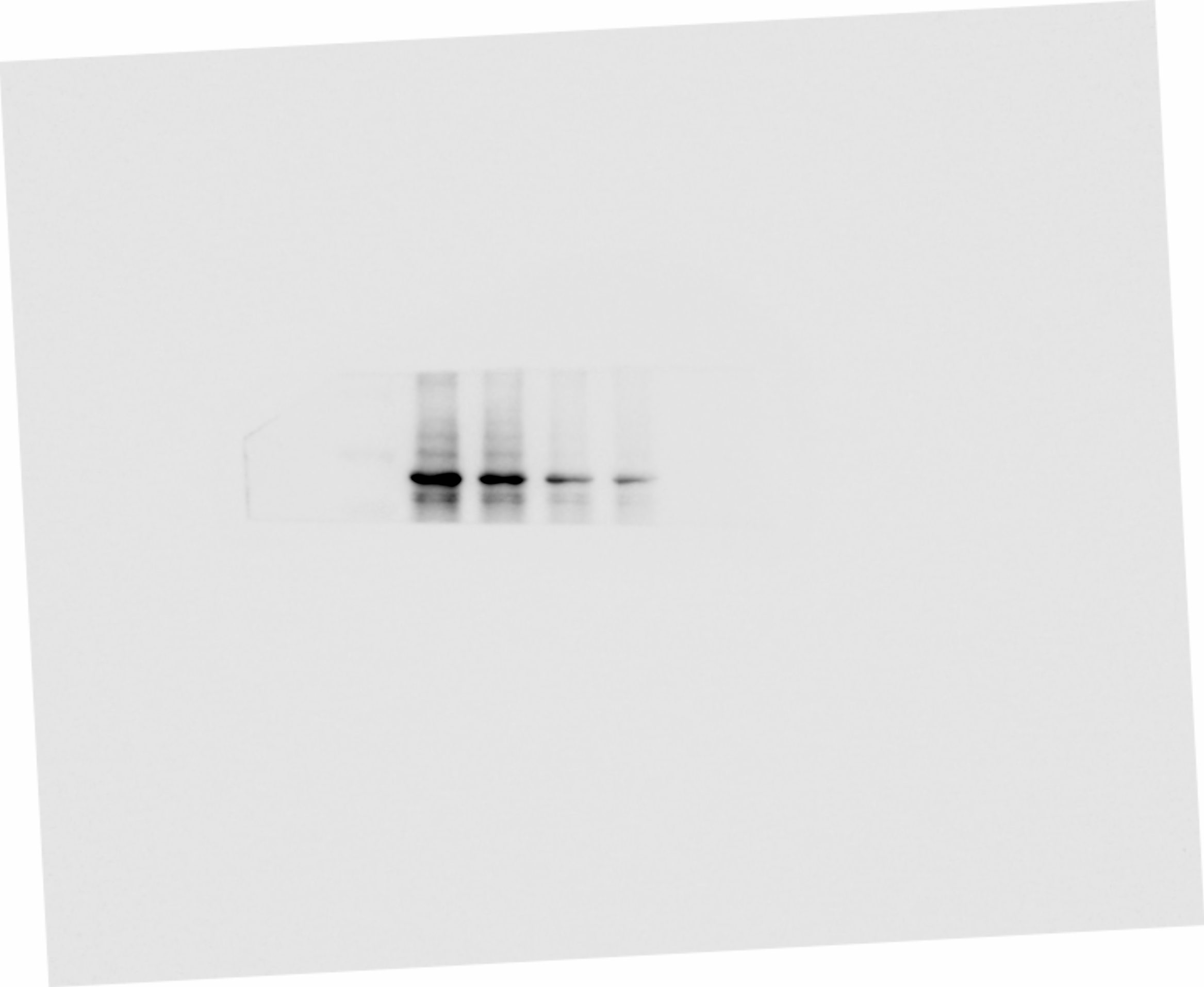

Supplement: Figure 6—source data 1. [file elife-100205-fig6-data1.zip › Figure 6-Source Data 1-Raw uncropped blots/Figure 6F/ADGRA3.tif]

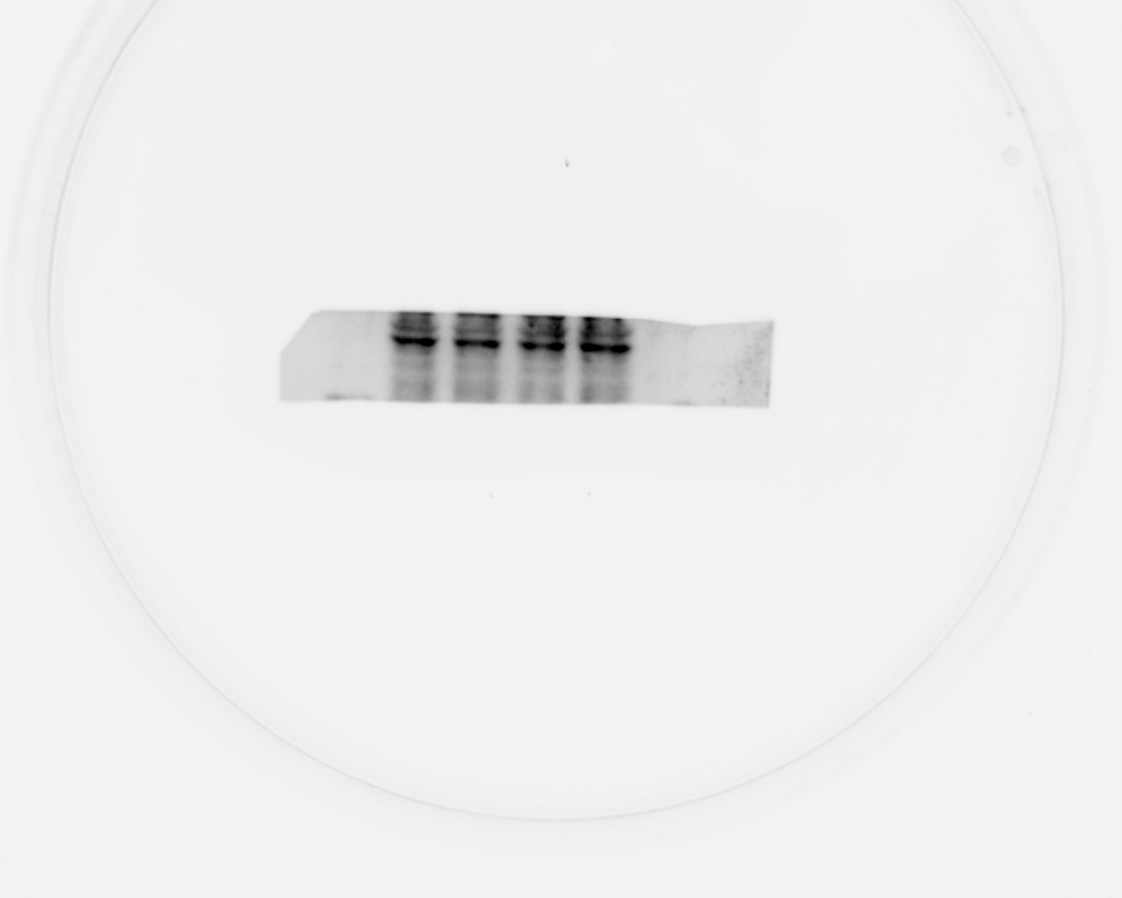

Supplement: Figure 6—source data 1. [file elife-100205-fig6-data1.zip › Figure 6-Source Data 1-Raw uncropped blots/Figure 6F/CREB.tif]

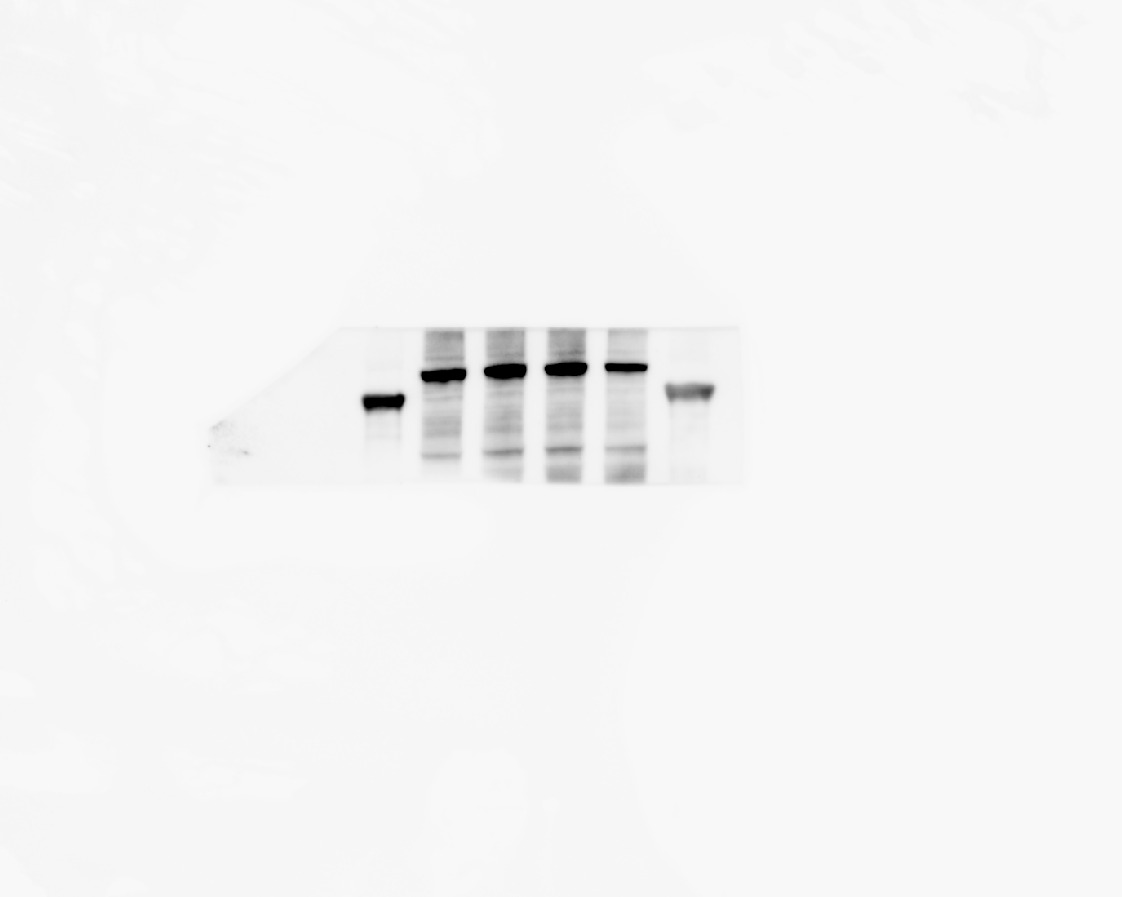

Supplement: Figure 6—source data 1. [file elife-100205-fig6-data1.zip › Figure 6-Source Data 1-Raw uncropped blots/Figure 6F/HSP90.tif]

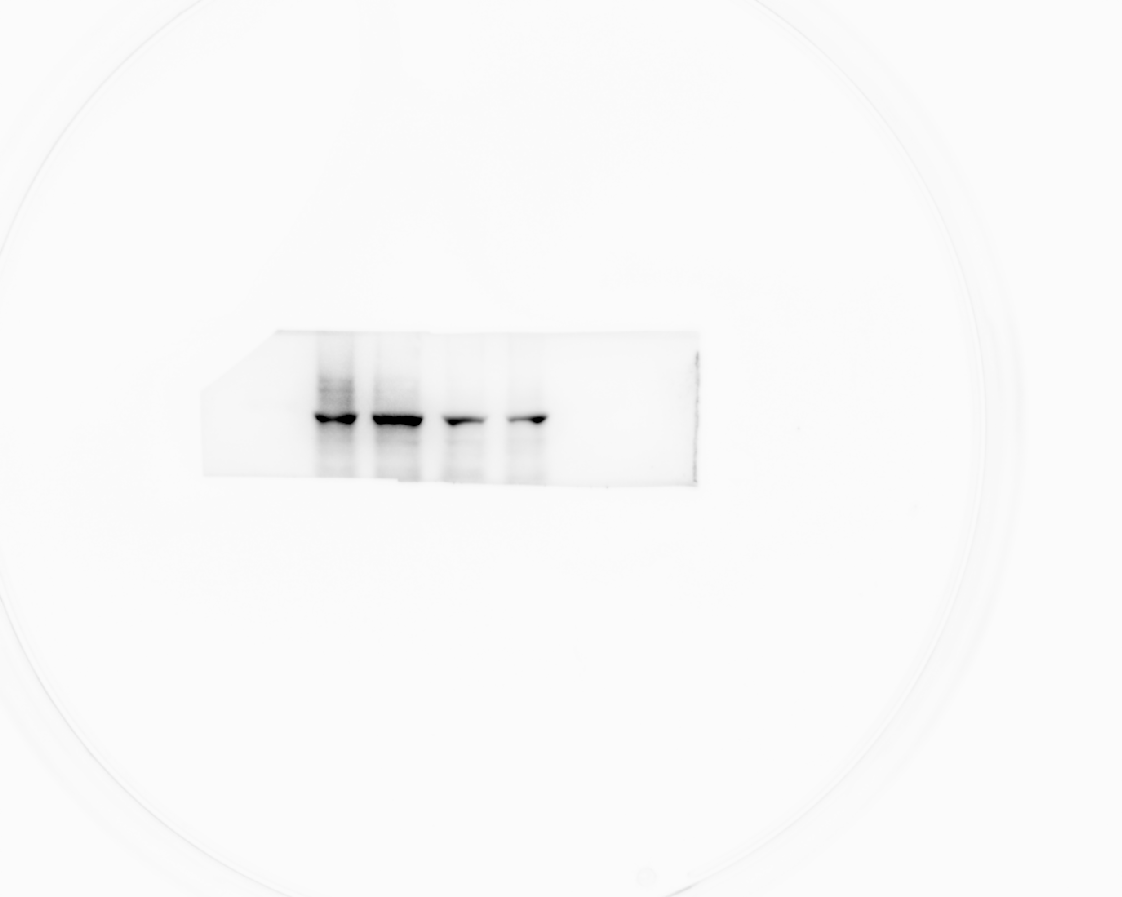

Supplement: Figure 6—source data 1. [file elife-100205-fig6-data1.zip › Figure 6-Source Data 1-Raw uncropped blots/Figure 6F/pCREB.tif]

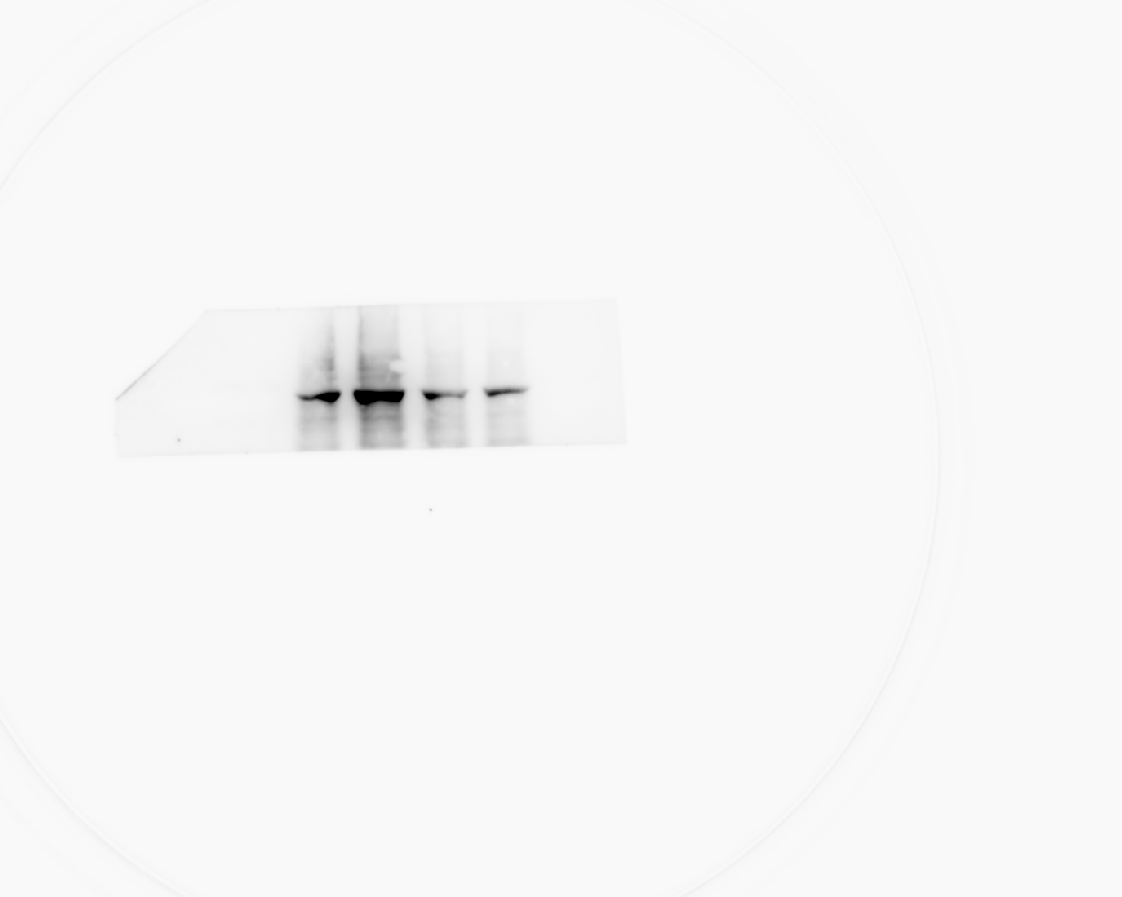

Supplement: Figure 6—source data 1. [file elife-100205-fig6-data1.zip › Figure 6-Source Data 1-Raw uncropped blots/Figure 6F/UCP1.tif]

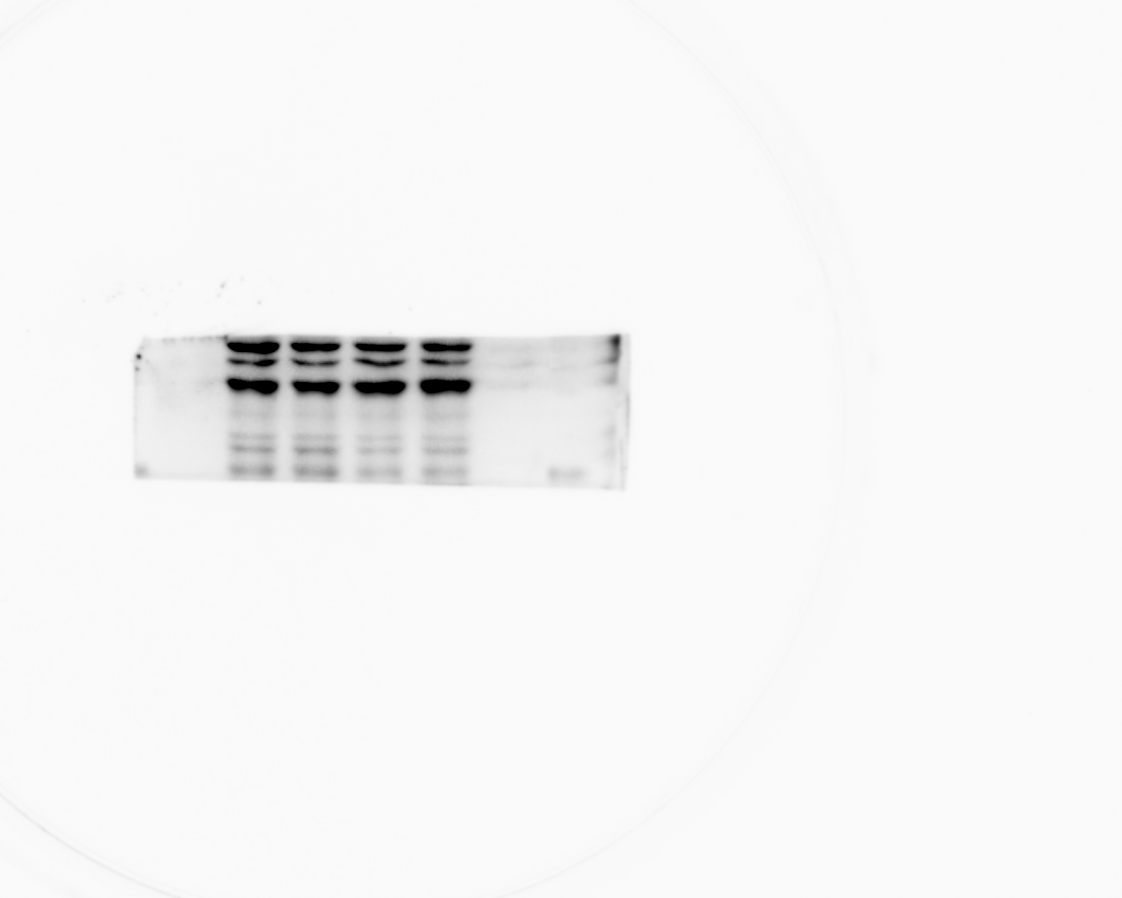

Supplement: Figure 6—source data 1. [file elife-100205-fig6-data1.zip › Figure 6-Source Data 1-Raw uncropped blots/Figure 6J/CREB.tif]

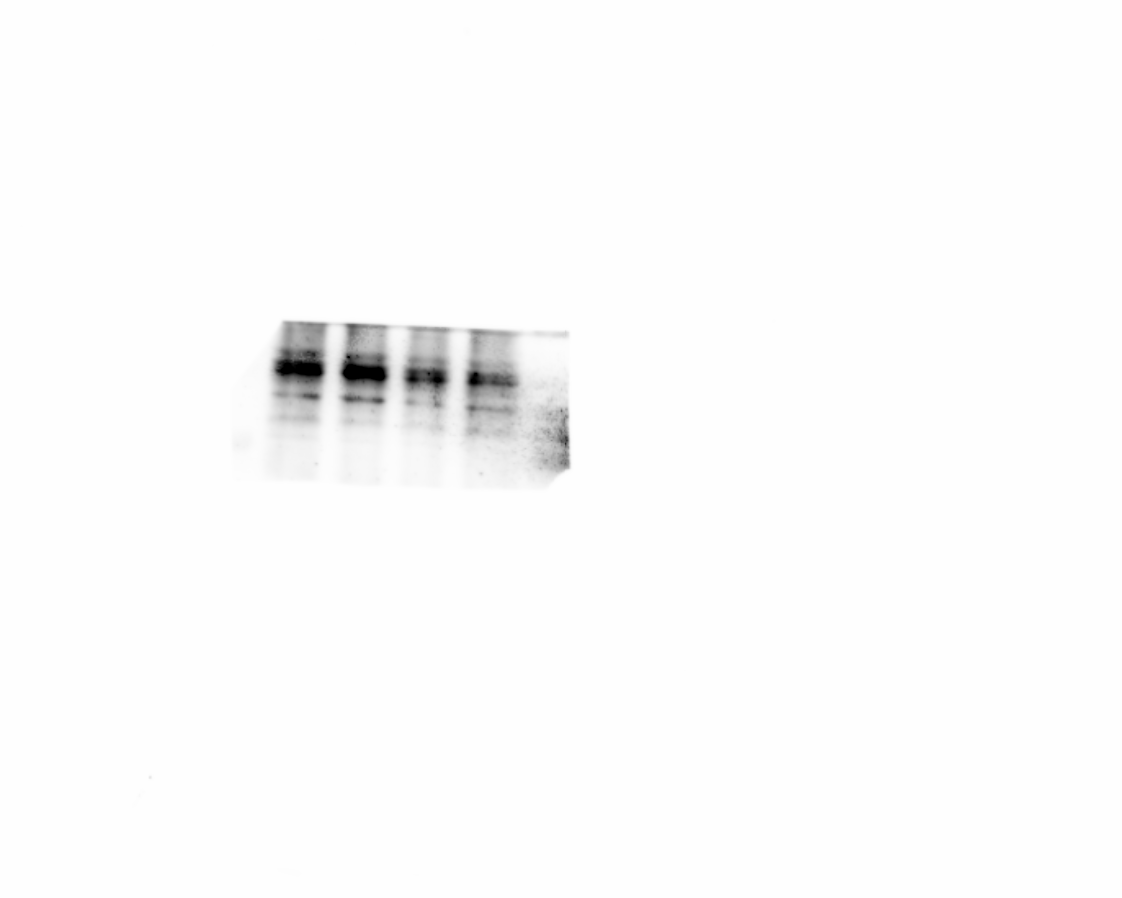

Supplement: Figure 6—source data 1. [file elife-100205-fig6-data1.zip › Figure 6-Source Data 1-Raw uncropped blots/Figure 6J/GNAS.tif]

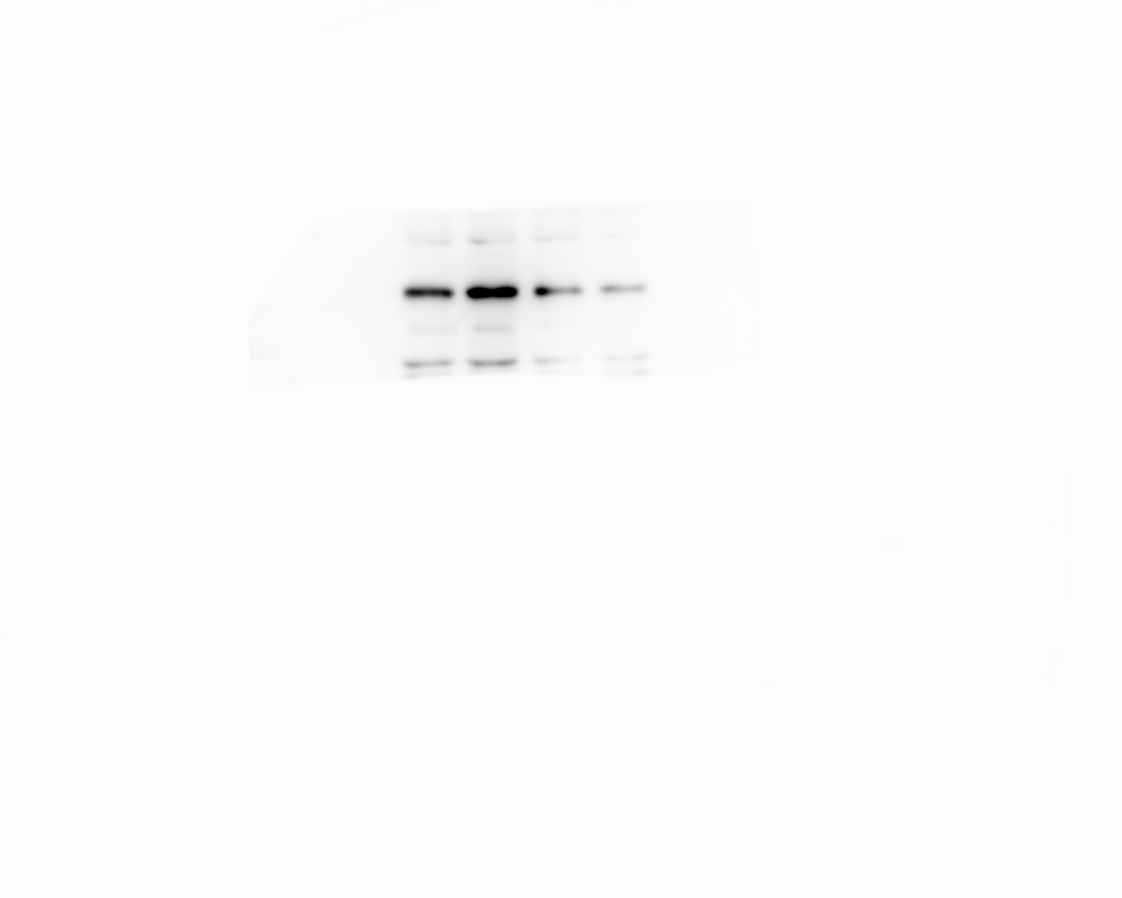

Supplement: Figure 6—source data 1. [file elife-100205-fig6-data1.zip › Figure 6-Source Data 1-Raw uncropped blots/Figure 6J/pCREB.tif]

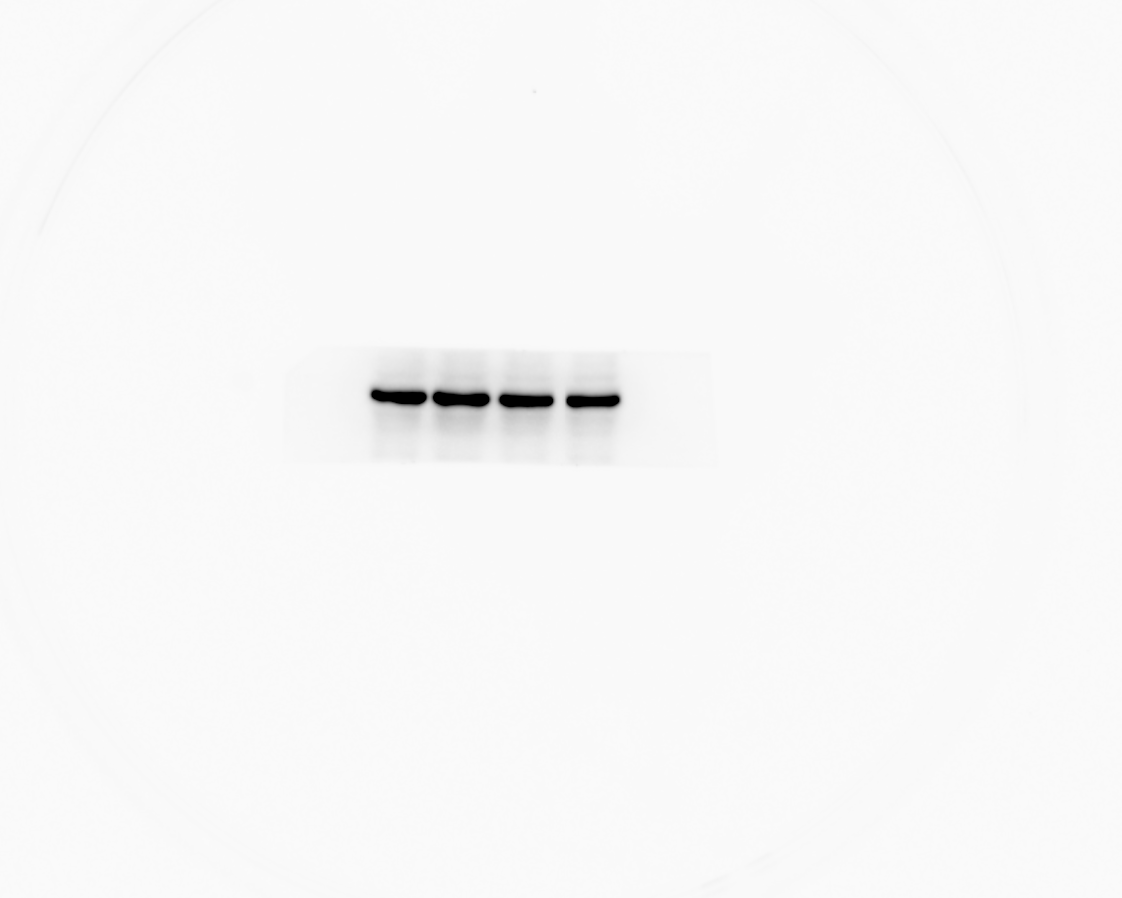

Supplement: Figure 6—source data 1. [file elife-100205-fig6-data1.zip › Figure 6-Source Data 1-Raw uncropped blots/Figure 6J/Tubulin.tif]

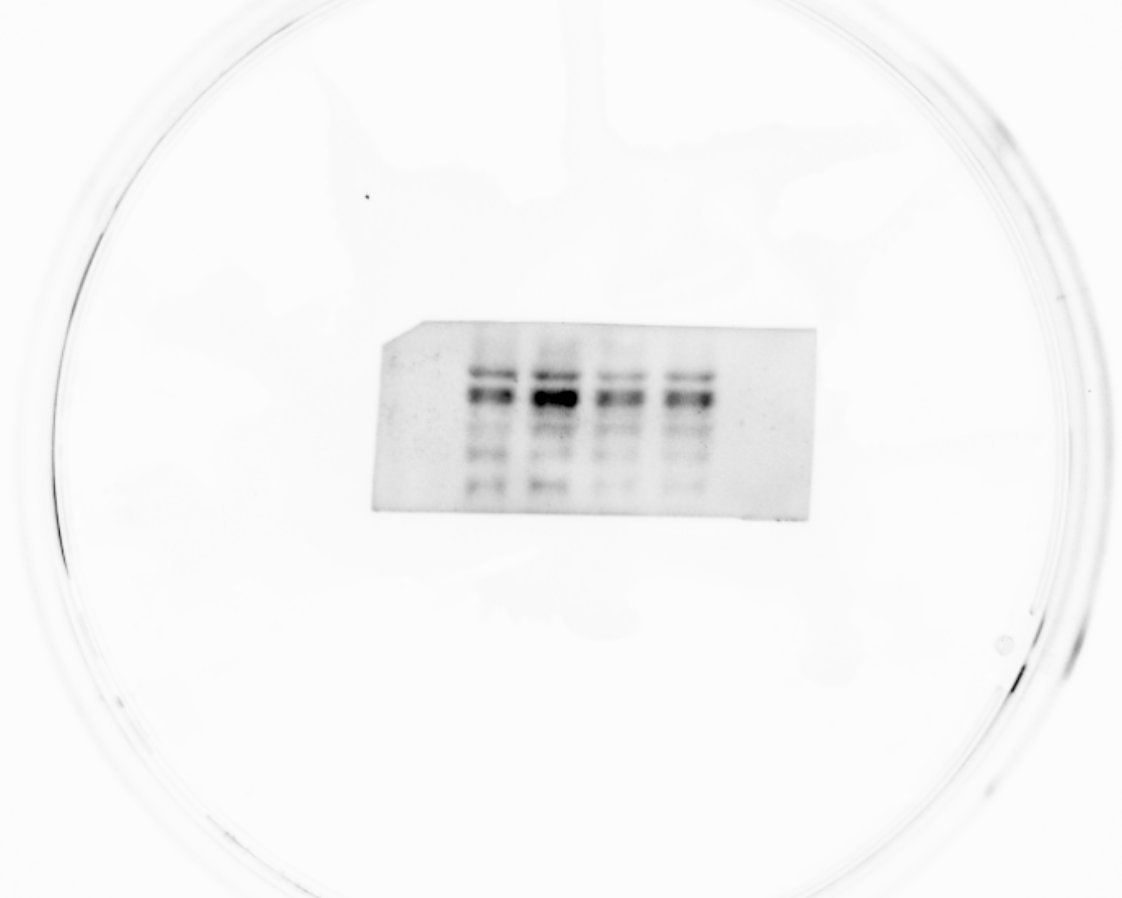

Supplement: Figure 6—source data 1. [file elife-100205-fig6-data1.zip › Figure 6-Source Data 1-Raw uncropped blots/Figure 6J/UCP1.tif]

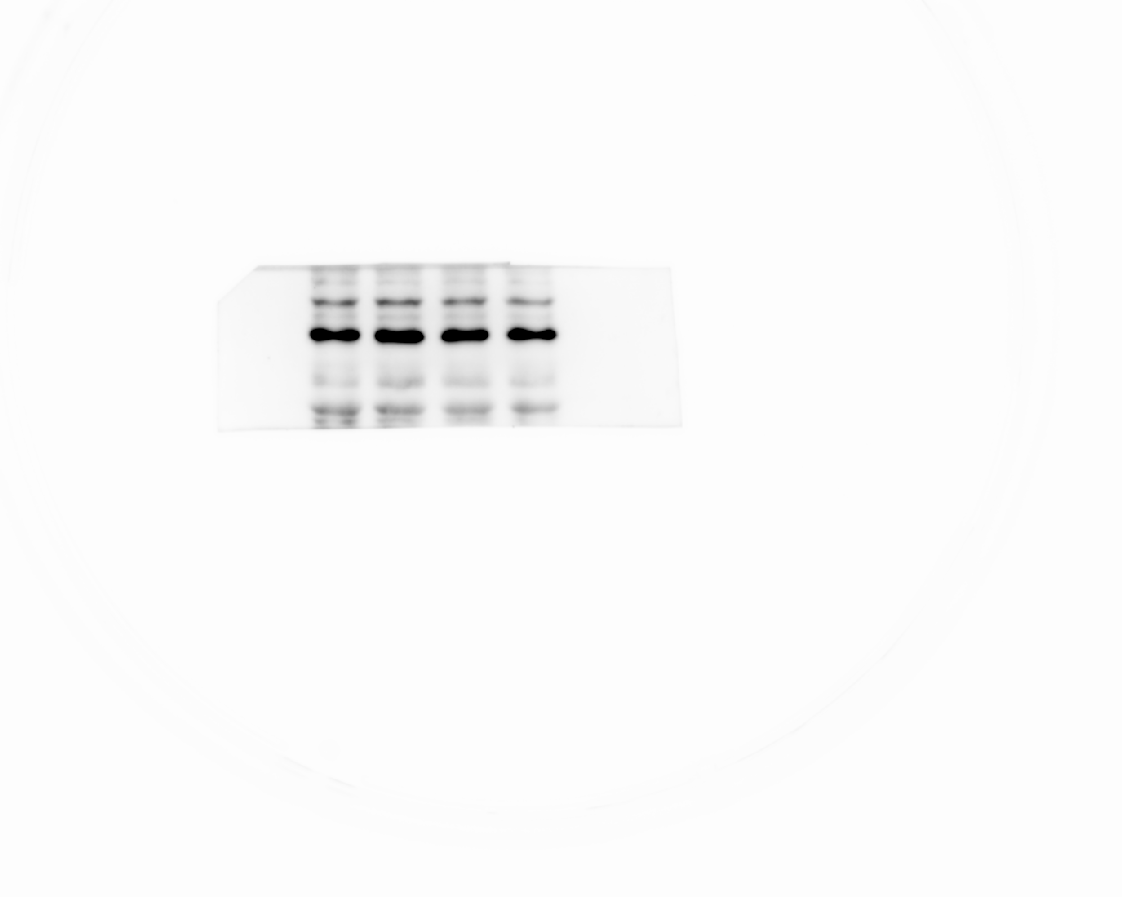

Supplement: Figure 6—source data 1. [file elife-100205-fig6-data1.zip › Figure 6-Source Data 1-Raw uncropped blots/Figure 6L/CREB.tif]
