# Supplementary material for: Constitutively active receptor ADGRA3 signaling induces adipose thermogenesis
Source: eLife. 2024 Dec 24;13:RP100205. doi: 10.7554/eLife.100205 (PMC11668527; doi:10.7554/eLife.100205)

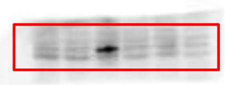
UCP1

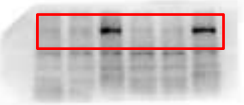
FLAG

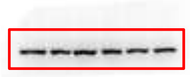
HSP90

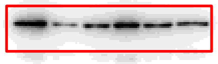
pCREB

CREB
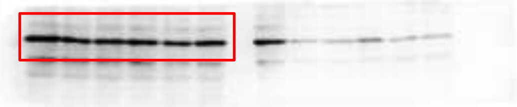

Supplement: Figure 5—source data 2. [file elife-100205-fig5-data2.zip › Figure 5-Source Data 2 -Uncropped and labeled blots/Figure 5J.pdf]
