# Supplementary figures and images for: Constitutively active receptor ADGRA3 signaling induces adipose thermogenesis (part 2 of 2)
Source: eLife. 2024 Dec 24;13:RP100205. doi: 10.7554/eLife.100205 (PMC11668527; doi:10.7554/eLife.100205)

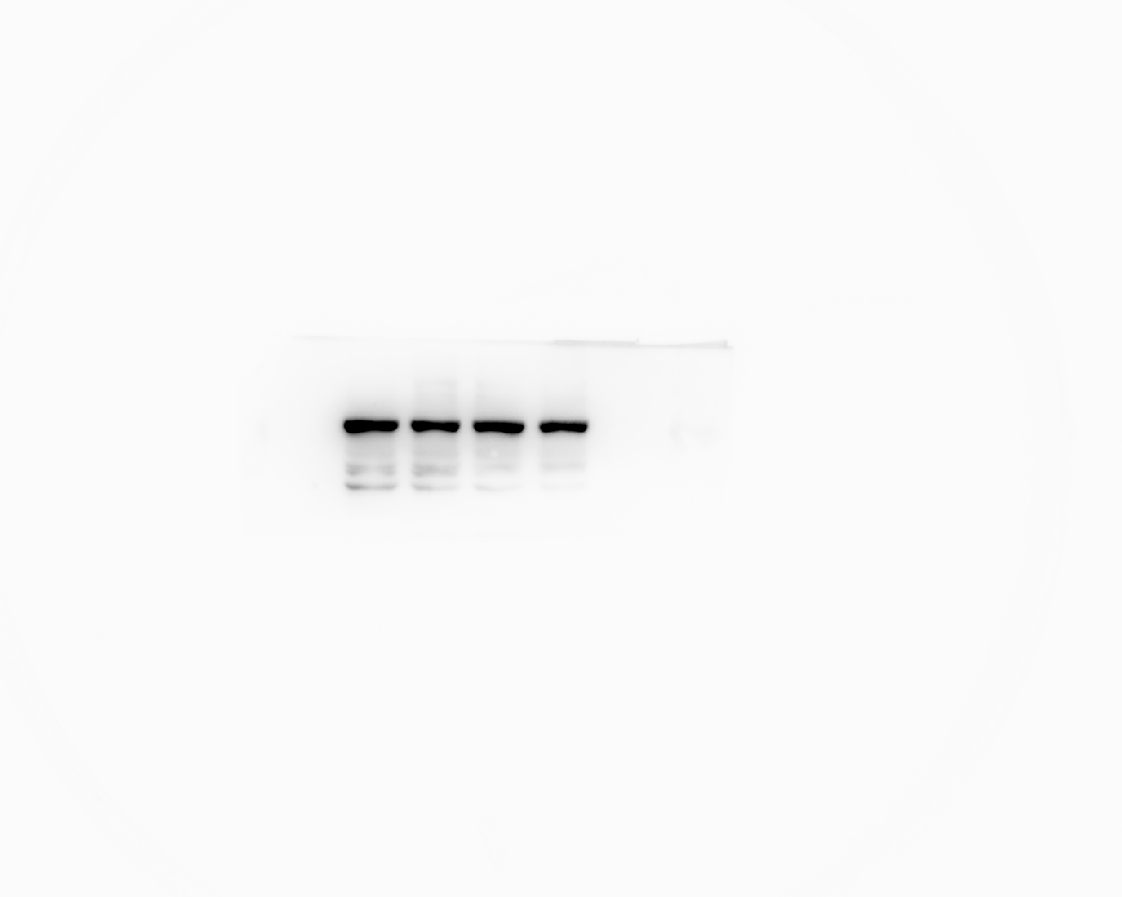

Supplement: Figure 6—source data 1. [file elife-100205-fig6-data1.zip › Figure 6-Source Data 1-Raw uncropped blots/Figure 6L/HSP90.tif]

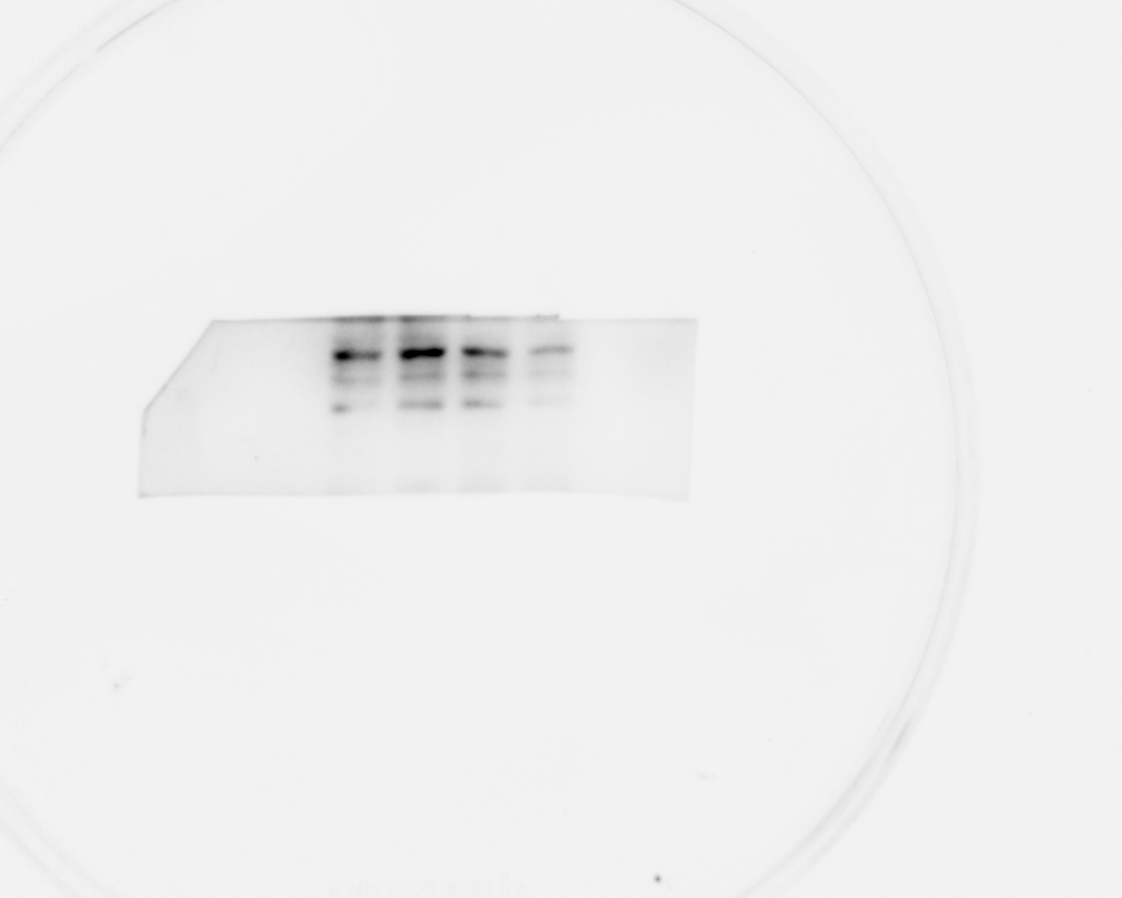

Supplement: Figure 6—source data 1. [file elife-100205-fig6-data1.zip › Figure 6-Source Data 1-Raw uncropped blots/Figure 6L/pCREB.tif]

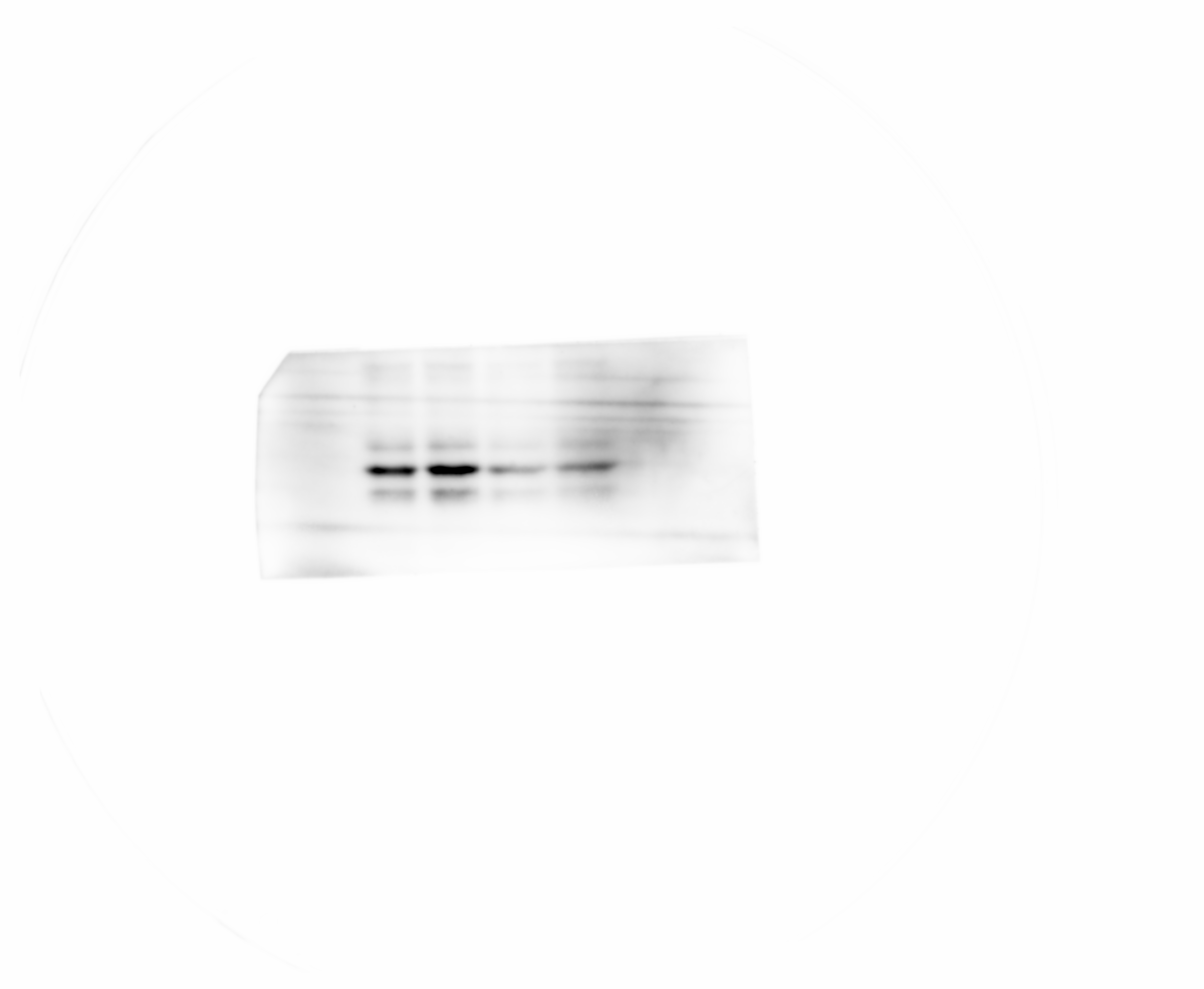

Supplement: Figure 6—source data 1. [file elife-100205-fig6-data1.zip › Figure 6-Source Data 1-Raw uncropped blots/Figure 6L/UCP1.tif]

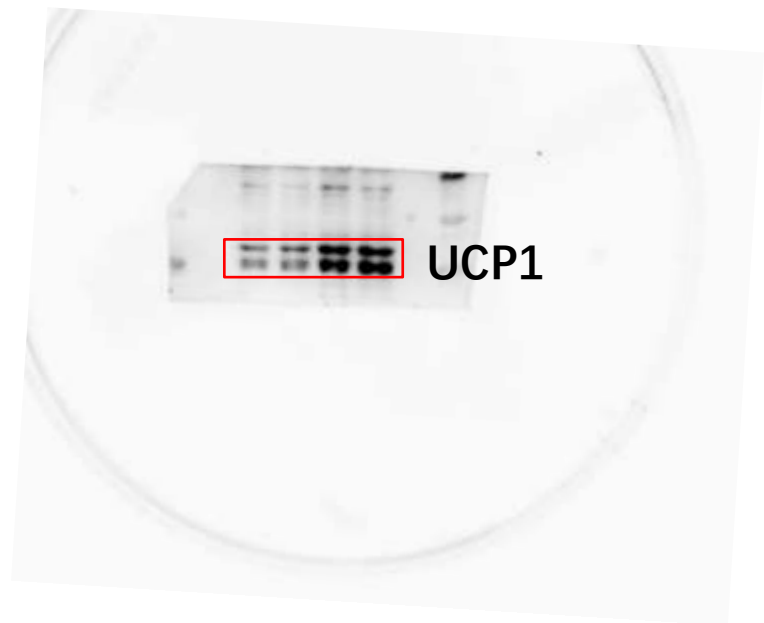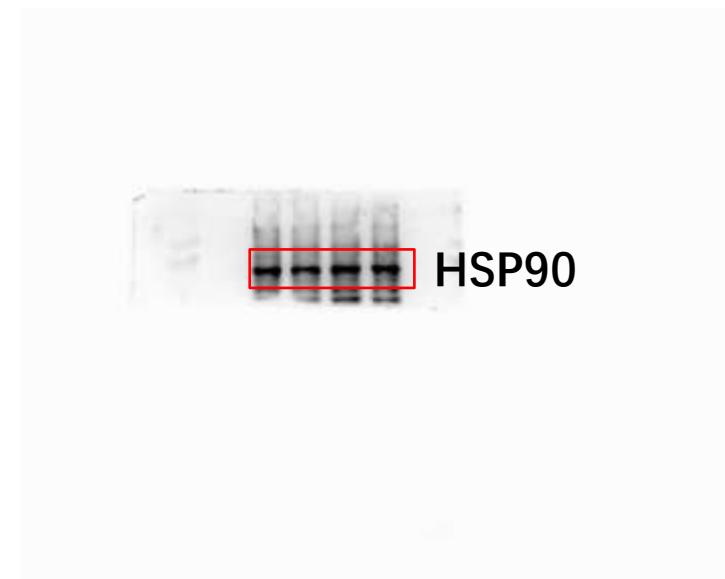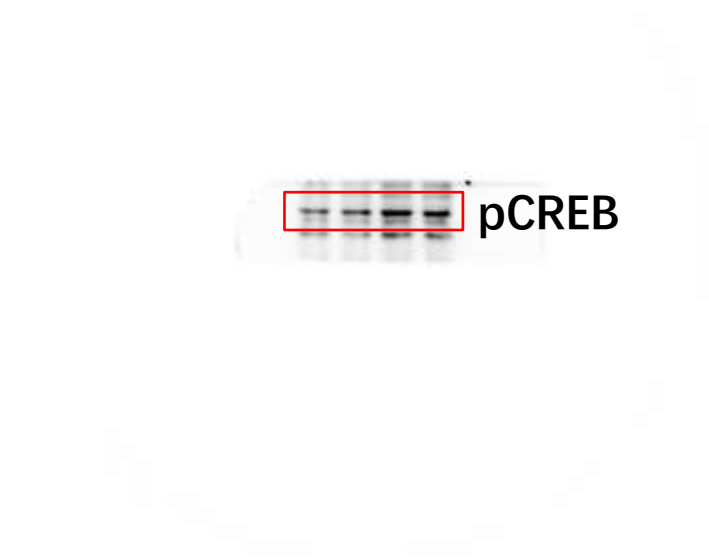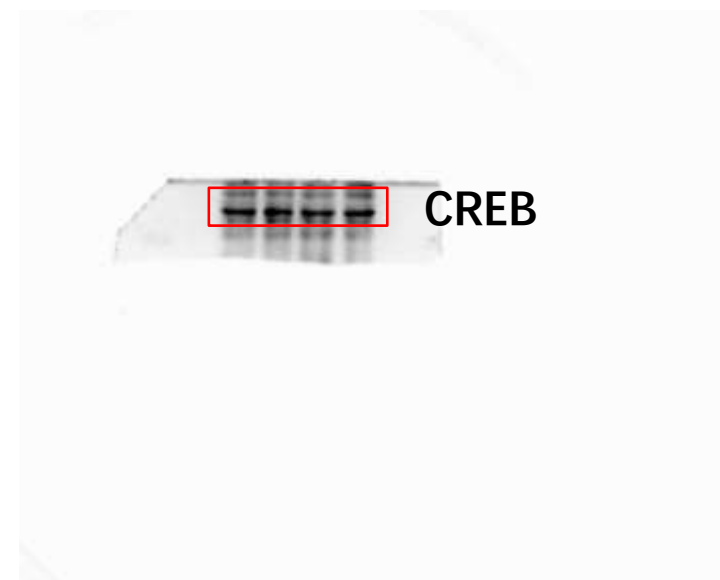

Supplement: Figure 6—source data 2. [file elife-100205-fig6-data2.zip › Figure 6-Source Data 2 -Uncropped and labeled blots/Figure 6D.pdf]

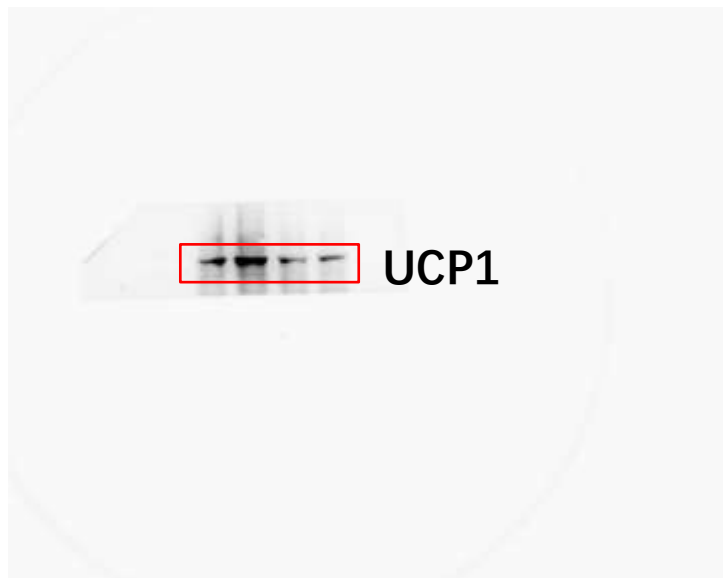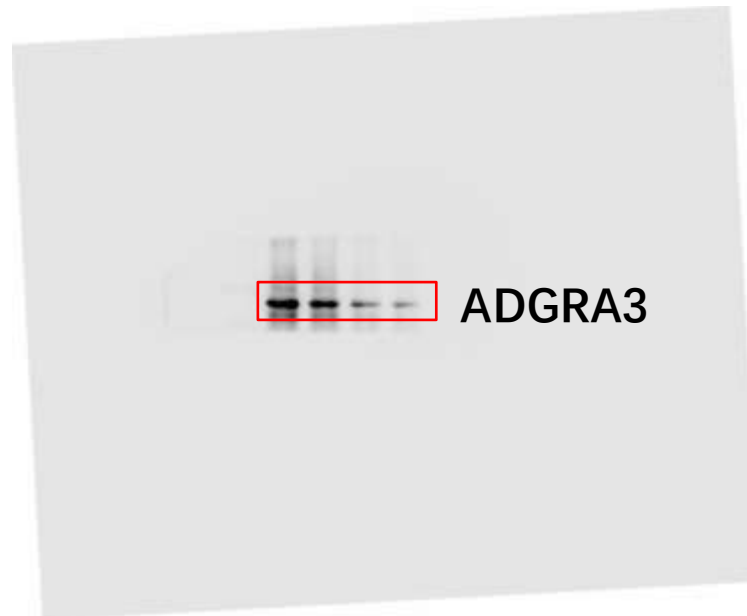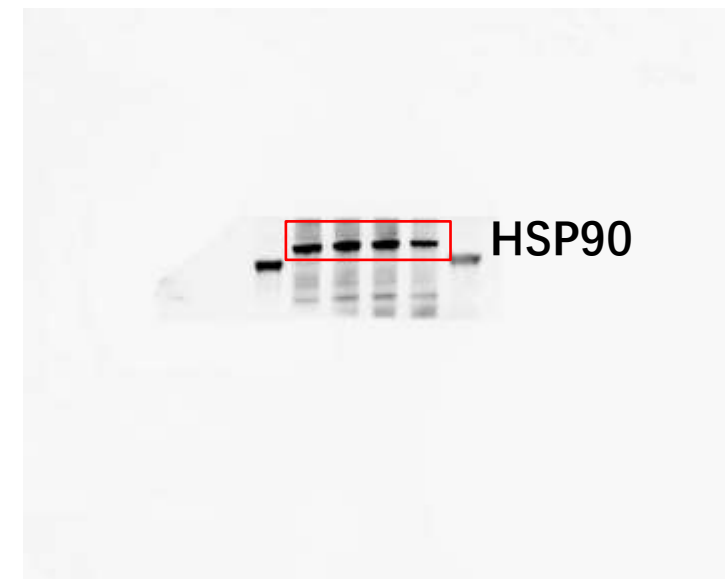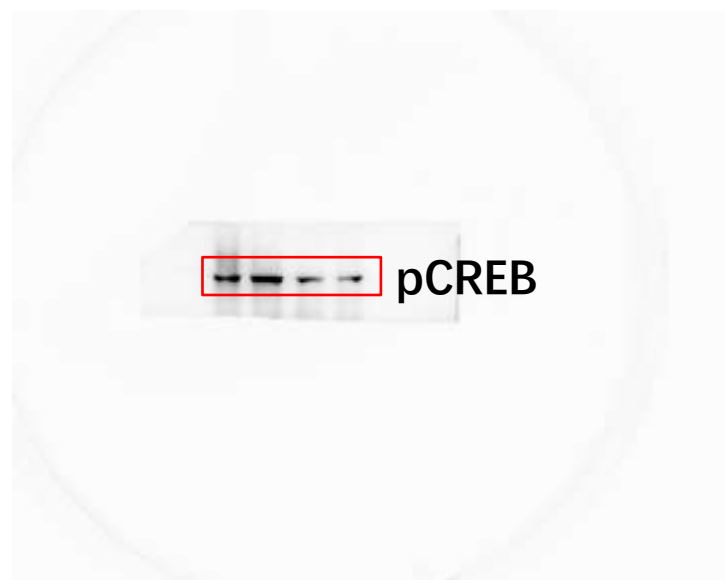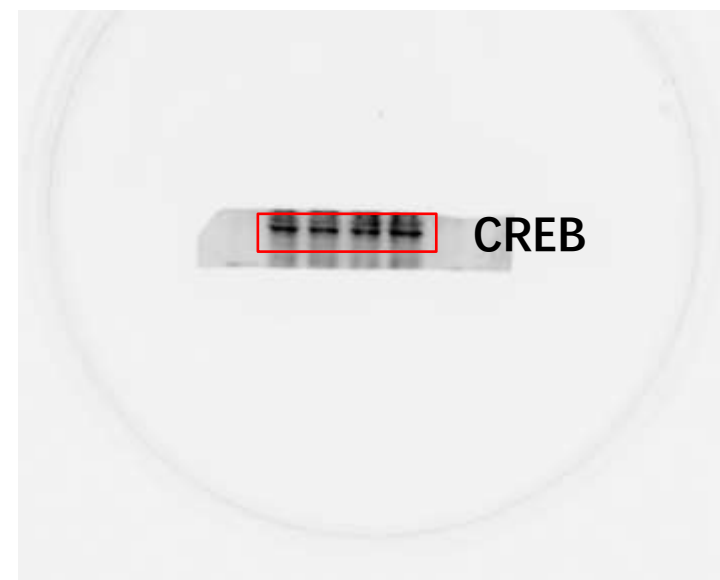

Supplement: Figure 6—source data 2. [file elife-100205-fig6-data2.zip › Figure 6-Source Data 2 -Uncropped and labeled blots/Figure 6F.pdf]

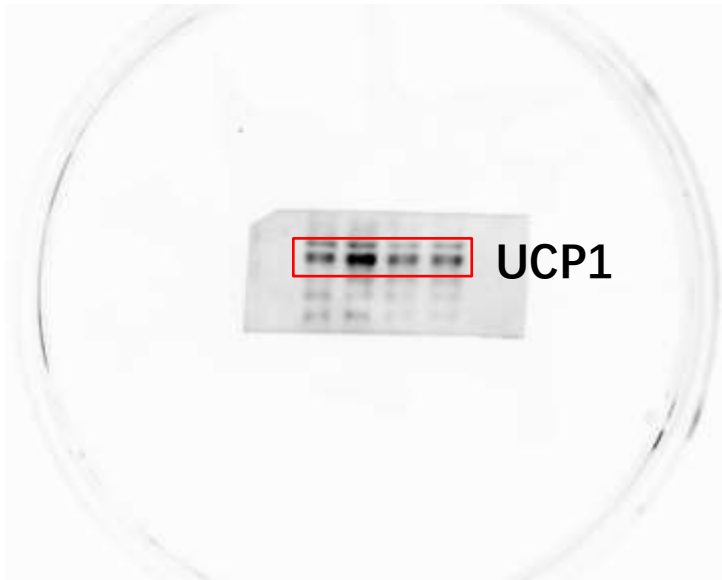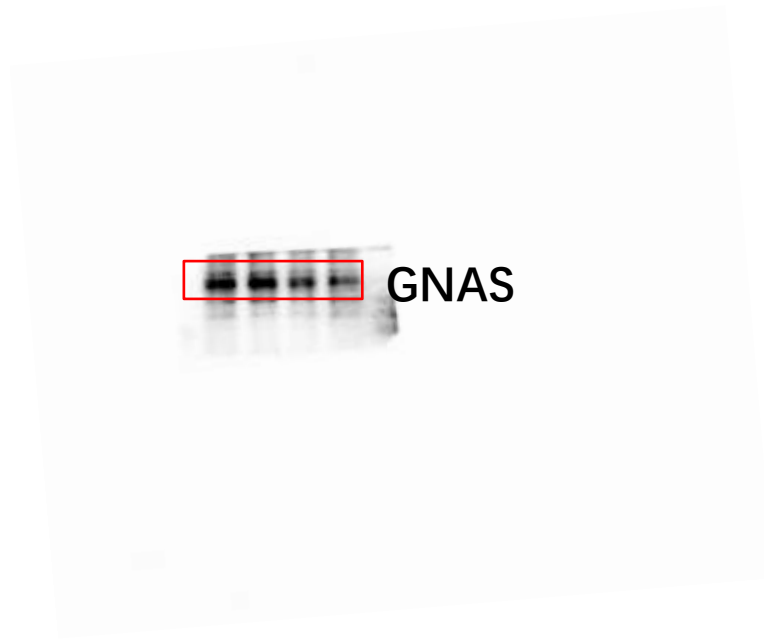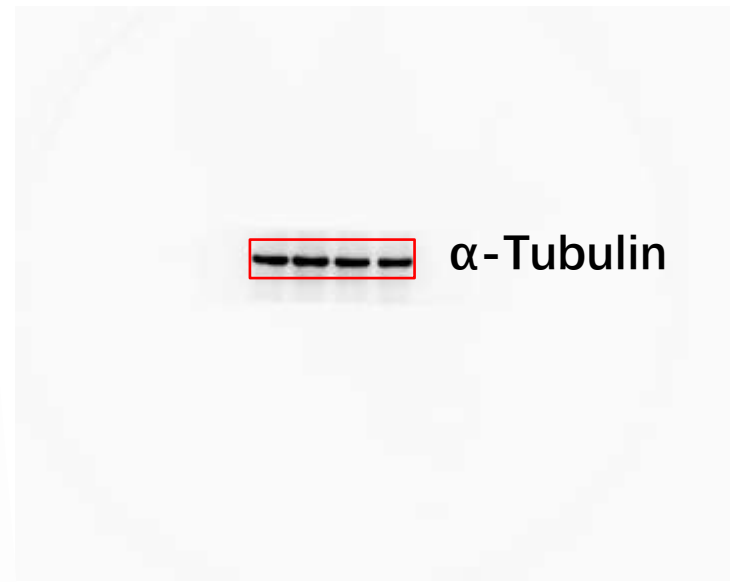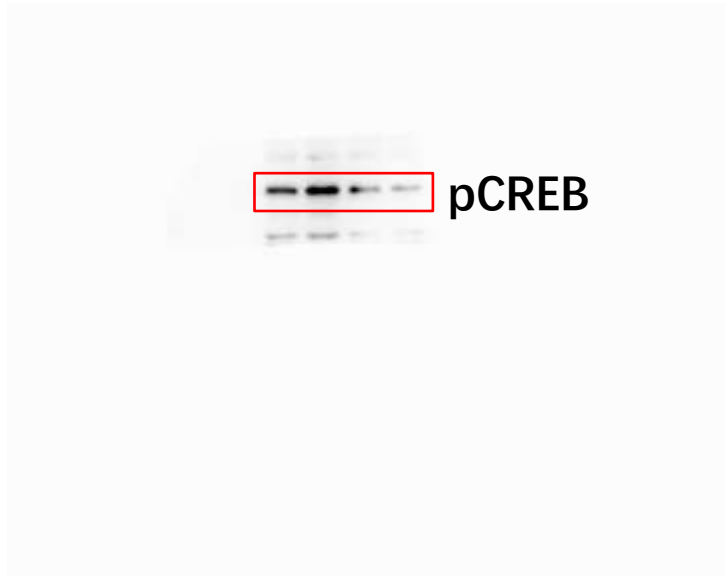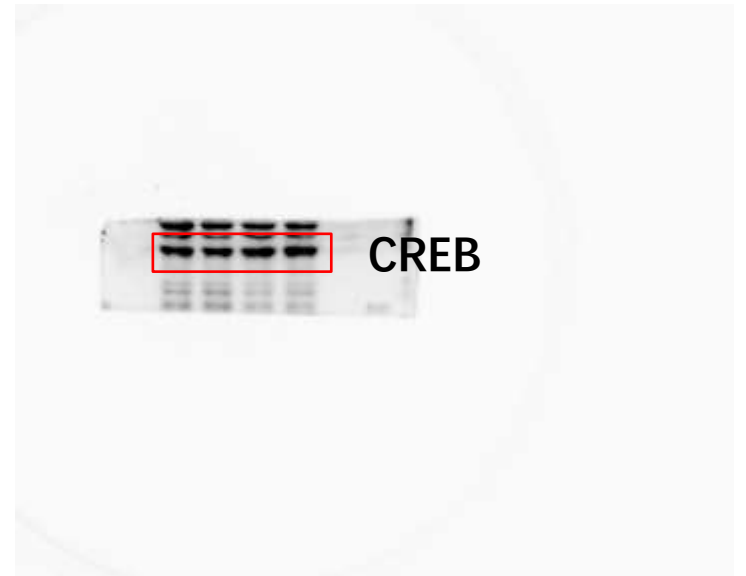

Supplement: Figure 6—source data 2. [file elife-100205-fig6-data2.zip › Figure 6-Source Data 2 -Uncropped and labeled blots/Figure 6J.pdf]

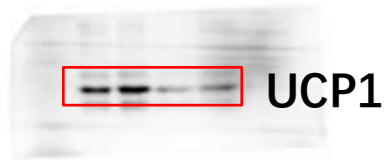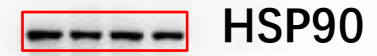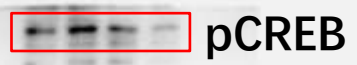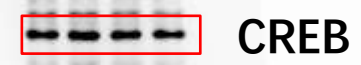

Supplement: Figure 6—source data 2. [file elife-100205-fig6-data2.zip › Figure 6-Source Data 2 -Uncropped and labeled blots/Figure 6L.pdf]
