# Supplementary material for: Constitutively active receptor ADGRA3 signaling induces adipose thermogenesis
Source: eLife. 2024 Dec 24;13:RP100205. doi: 10.7554/eLife.100205 (PMC11668527; doi:10.7554/eLife.100205)
Supplement: Supplementary file 2. — The 1134 screened genes were annotated by David database and 27 of these genes were identified as G-protein coupled receptor encoding gene. [file elife-100205-supp2.docx]

| \| **Gene ID** \| **Gene Name** \| **GO Annotation** \| \| --- \| --- \| --- \| \| 14748 \| ***Gpr3*** \| GO0004930: G-protein coupled receptor activity \| \| 70693 \| ***Adgra3*** \| GO0004930: G-protein coupled receptor activity \| \| 224792 \| ***Adgrf5*** \| GO0004930: G-protein coupled receptor activity \| \| 170757 \| ***Adgrl4*** \| GO0004930: G-protein coupled receptor activity \| \| 11549 \| ***Adra1a*** \| GO0004930: G-protein coupled receptor activity \| \| 11554 \| ***Adrb1*** \| GO0004930: G-protein coupled receptor activity \| \| 12778 \| ***Ackr3*** \| GO0004930: G-protein coupled receptor activity \| \| 12922 \| ***Crhr2*** \| GO0004930: G-protein coupled receptor activity \| \| 13618 \| ***Ednrb*** \| GO0004930: G-protein coupled receptor activity \| \| 14368 \| ***Fzd6*** \| GO0004930: G-protein coupled receptor activity \| \| 14427 \| ***Galr1*** \| GO0004930: G-protein coupled receptor activity \| \| 242425 \| ***Gabbr2*** \| GO0004930: G-protein coupled receptor activity \| \| 329252 \| ***Lgr6*** \| GO0004930: G-protein coupled receptor activity \| \| 78134 \| ***Lpar4*** \| GO0004930: G-protein coupled receptor activity \| \| 258218 \| ***Olfr102*** \| GO0004930: G-protein coupled receptor activity \| \| 258027 \| ***Olfr1385*** \| GO0004930: G-protein coupled receptor activity \| \| 258334 \| ***Olfr1396*** \| GO0004930: G-protein coupled receptor activity \| \| 634104 \| ***Olfr287*** \| GO0004930: G-protein coupled receptor activity \| \| 259010 \| ***Olfr393*** \| GO0004930: G-protein coupled receptor activity \| \| 258816 \| ***Olfr466*** \| GO0004930: G-protein coupled receptor activity \| \| 259097 \| ***Olfr558*** \| GO0004930: G-protein coupled receptor activity \| \| 259062 \| ***Olfr667*** \| GO0004930: G-protein coupled receptor activity \| \| 258469 \| ***Olfr90*** \| GO0004930: G-protein coupled receptor activity \| \| 258825 \| ***Olfr975*** \| GO0004930: G-protein coupled receptor activity \| \| 18441 \| ***P2ry1*** \| GO0004930: G-protein coupled receptor activity \| \| 94226 \| ***S1pr5*** \| GO0004930: G-protein coupled receptor activity \| \| 21337 \| ***Tacr2*** \| GO0004930: G-protein coupled receptor activity \|   **Supplementary File 2. Gene annotation for screened genes.** The 1134 screened genes were annotated by David database and 27 of these genes were identified as G-protein coupled receptor encoding gene. |
| --- | --- | --- | --- | --- | --- | --- | --- | --- | --- | --- | --- | --- | --- | --- | --- | --- | --- | --- | --- | --- | --- | --- | --- | --- | --- | --- | --- | --- | --- | --- | --- | --- | --- | --- | --- | --- | --- | --- | --- | --- | --- | --- | --- | --- | --- | --- | --- | --- | --- | --- | --- | --- | --- | --- | --- | --- | --- | --- | --- | --- | --- | --- | --- | --- | --- | --- | --- | --- | --- | --- | --- | --- | --- | --- | --- | --- | --- | --- | --- | --- | --- | --- | --- | --- |
